# Machine Learning-Based Diabetic Neuropathy and Previous Foot Ulceration Patients Detection Using Electromyography and Ground Reaction Forces during Gait

**DOI:** 10.3390/s22093507

**Published:** 2022-05-05

**Authors:** Fahmida Haque, Mamun Bin Ibne Reaz, Muhammad Enamul Hoque Chowdhury, Maymouna Ezeddin, Serkan Kiranyaz, Mohammed Alhatou, Sawal Hamid Md Ali, Ahmad Ashrif A Bakar, Geetika Srivastava

**Affiliations:** 1 Department of Electrical, Electronic and System Engineering, Universiti Kebangsaan Malaysia, Bangi 43600, Malaysia; P97694@siswa.ukm.edu.my (F.H.); sawal@ukm.edu.my (S.H.M.A.); ashrif@ukm.edu.my (A.A.A.B.); 2 Department of Electrical Engineering, Qatar University, Doha 2713, Qatar; me1511677@qu.edu.qa (M.E.); mkiranyaz@qu.edu.qa (S.K.); 3 Neuromuscular Division, Hamad General Hospital, Doha 3050, Qatar; malhatou@hamad.qa; 4 Department of Neurology, Al khor Hospital, Doha 3050, Qatar; 5 Department of Physics and Electronics, Dr. Ram Manohar Lohia Avadh University, Faizabad, Uttar Pradesh 224001, India; gsrivastava@rmlau.ac.in

**Keywords:** diabetic neuropathy, diabetic foot ulceration, biomechanical parameters, electromyography, EMG, gait, ground reaction force, machine learning, feature selection

## Abstract

Diabetic neuropathy (DN) is one of the prevalent forms of neuropathy that involves alterations in biomechanical changes in the human gait. Diabetic foot ulceration (DFU) is one of the pervasive types of complications that arise due to DN. In the literature, for the last 50 years, researchers have been trying to observe the biomechanical changes due to DN and DFU by studying muscle electromyography (EMG) and ground reaction forces (GRF). However, the literature is contradictory. In such a scenario, we propose using Machine learning techniques to identify DN and DFU patients by using EMG and GRF data. We collected a dataset from the literature which involves three patient groups: Control (n = 6), DN (n = 6), and previous history of DFU (n = 9) and collected three lower limb muscles EMG (tibialis anterior (TA), vastus lateralis (VL), gastrocnemius lateralis (GL)), and three GRF components (GRFx, GRFy, and GRFz). Raw EMG and GRF signals were preprocessed, and different feature extraction techniques were applied to extract the best features from the signals. The extracted feature list was ranked using four different feature ranking techniques, and highly correlated features were removed. In this study, we considered different combinations of muscles and GRF components to find the best performing feature list for the identification of DN and DFU. We trained eight different conventional ML models: Discriminant analysis classifier (DAC), Ensemble classification model (ECM), Kernel classification model (KCM), k-nearest neighbor model (KNN), Linear classification model (LCM), Naive Bayes classifier (NBC), Support vector machine classifier (SVM), and Binary decision classification tree (BDC), to find the best-performing algorithm and optimized that model. We trained the optimized the ML algorithm for different combinations of muscles and GRF component features, and the performance matrix was evaluated. Our study found the KNN algorithm performed well in identifying DN and DFU, and we optimized it before training. We found the best accuracy of 96.18% for EMG analysis using the top 22 features from the chi-square feature ranking technique for features from GL and VL muscles combined. In the GRF analysis, the model showed 98.68% accuracy using the top 7 features from the Feature selection using neighborhood component analysis for the feature combinations from the GRFx-GRFz signal. In conclusion, our study has shown a potential solution for ML application in DN and DFU patient identification using EMG and GRF parameters. With careful signal preprocessing with strategic feature extraction from the biomechanical parameters, optimization of the ML model can provide a potential solution in the diagnosis and stratification of DN and DFU patients from the EMG and GRF signals.

## 1. Introduction

Diabetic neuropathy (DN) is one of the prevalent forms of neuropathy observed in diabetic patients. It exhibits a deleterious effect on the biomechanical system of the patients, especially showing disorder in the walking cycle, known as the gait cycle [1,2,3,4]. Diabetic neuropathy intensifies the sensitivity loss in somatosensory nerves and dysfunctionality in distal muscles, especially in the lower limbs, which are the prominent reason for the alteration in the electrophysiological and biomechanical system during gait [5,6,7,8,9,10]. Due to the changes in biomechanics during gait, a patient suffers from alteration to plantar pressure, kinematic patterns, ground reaction forces, and muscle activities. Long-term alteration to the biomechanics leads to foot ulceration and, in worsening cases, amputation of the lower limb [5,6,7,11,12,13,14,15,16]. Foot ulceration is one of the pervasive types of long-term chronic complications in DN patients, which indicates worsening DN [17,18].

From the literature, it can be found that due to DN, patients exhibited delayed muscle activation peak of the tibialis anterior (TA), vastus lateralis (VL), gastrocnemius lateralis (GL), and gastrocnemius medialis (GM) muscles in different phases of the gait cycle [4,5,8,9,11,12,14,19,20,21]. The changes in lower limb muscle activities during gait of DN patients are also related to other alterations, such as higher plantar pressure distribution, greater stance phase, modified ground reaction forces (GRF), and moments of force [6,12,13,22,23,24] In Akashi et al.’s study [5], they showed that DN patients with and without a history of plantar ulceration had a delayed activation peak and a decrease in the second peak of vertical ground reaction force comparing with the control group in VL and GL muscles during gait. Another study by Sacco et al. [6] and Abboud et al. [22] showed delayed muscle activation and decreased muscle amplitude of TA muscle for DN patients. Sawacha et al. [15] found an alteration in gait in DN patients and suggested that this can be a predictive indicator for the risk of ulceration.

Although these studies claim the relationship between the alteration of lower limb muscles and DN, few studies reported prolonged activity in VL, GL, GM, and rectus femoris muscles and GRF in patients without DN [5,14,20,22,24]. With these controversies in studies regarding the biomechanical changes in lower limb muscles for patients with and without DN, such claims are not reliable just by analyzing EMG and GRF signals. Depending on the variability of the population recruited for these studies, the biomechanical changes in muscle EMG and GRF during gait can be varied among the same experimental groups, depending on the patient’s ability to adapt to the changes in gait. Patients did not exhibit any patterned delay in the muscle activation functions, which can cause unreliable diagnosis [6,12]. With the help of Machine Learning (ML), EMG and GRF signal analysis can be made more robust and overcome the variability in the research regarding the patient’s identification.

Foot ulceration is the main precursor to lower limb amputation in patients with diabetes worldwide [24]. Most foot ulcers are triggered by diabetes-associated peripheral neuropathy (DPN) [25,26]. A study by Allen et al. [27] showed that 23% of those with a diabetic foot ulcer had DPN, compared to only 6% of those without a foot ulcer. It can be hypothesized that due to DPN, patients with active plantar DFUs would continue to demonstrate similar abnormal lower limb biomechanical characteristics as displayed before the DFU formation [17,28]. A nonhealing diabetic foot pressure ulcer could eventually lead to amputation of the foot [29].

For the past 50 years, researchers have been using EMG signals to identify neuromuscular diseases by collecting the signals from the diagnosed region of interest in the human body and analyzing them manually [30]. However, manual analysis of bio-signals such as EMG can be challenging. As previously discussed, EMG signals in DN patients might not always follow any specific pattern. In such cases, the raw signal analysis does not provide much information from the EMG signals. In such cases, from the EMG and GRF signals, with the help of machine learning, many important features can be extracted using feature extraction techniques [31]. In addition, the classification of different disease classes with the help of ML by using EMG is becoming popular.

In recent literature, the use of EMG-based pattern recognition systems [32,33], human-machine interaction [34,35], and myoelectric controller [36] with ML is becoming popular. However, its application in disease identification is very limited, especially in DN diagnosis. Fahmida et al. [9] proposed an adaptive neuro-fuzzy inference system-based classifier for diabetic sensorimotor polyneuropathy (DSPN) severity classification by using features from three lower limb muscles’ (TA, VL, GM) EMGs during gait. Their study used only two EMG features based on the current literature time for activation peak and peak magnitude during gait with the progression of DSPN severity. They trained two different models; in one model, they used only the peak magnitudes from three different lower limb muscles, and in another model, they considered both the features of all three muscles. They reported 96% and 80% accuracy for both models, respectively.

Taking this into account, the objective of this work is to contribute to the state-of-the-art focus on the ML-based classification technique of DN and diabetic foot ulceration (DFU) patients by using EMG and GRF. As per our knowledge, this is the first ML-based work to classify DN and DUF patients using EMG and GRF data during gait. In this study, we collected data from the Akashi et al. [5] study, where they took three patients’ classes, control, DN, and DFU, and observed the EMG and GRF characteristics during gait. After the raw data preprocessing, we used the feature extraction technique on the EMG and GRF data. For EMG data, we used the feature extraction technique previously proposed by our team [37], where two novel time–domain features with another 17 existing time–domain features were combined. For GRF data, we extracted 195-time, frequency, and time–frequency domain features. For both EMG and GRA, extracted features were ranked using different ML-based feature ranking techniques, and highly correlated data were removed. We trained different conventional machine learning algorithms to find the best performing algorithm. After finding the best-performing algorithm, we tuned the hyperparameters of the ML algorithm and trained them for different cases, and their performance was evaluated. Our main objective was to find the best performing feature combination from EMG and GRF with the best performing ML model to accurately classify DN and DFU patients.

This research helps to find the best performing optimized ML model with best performing EMG and GRF features for identifying DN and DFU patients. As per our knowledge, this is the first ML-based work to classify DN and DUF patients using EMG and GRF data during gait. This study helps to work as a secondary decision system to analyze EMG and GRF data and accurately identify DN and DFU patients.

## 2. Materials and Methods

### 2.1. Dataset Description

**Figure 1:**
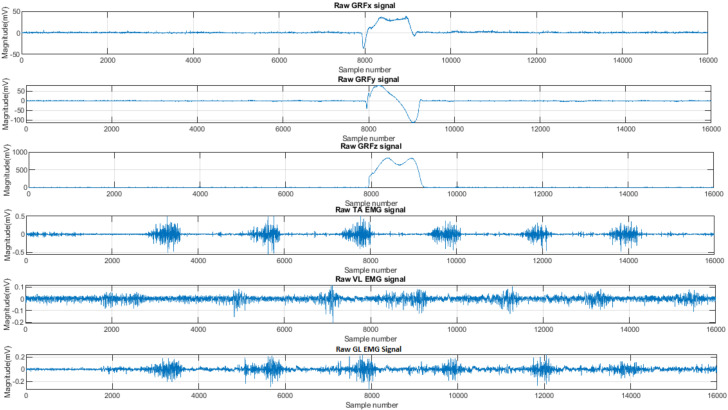
Sample of raw EMG signals from three lower limb muscles and 3-dimensional GRF signals Reprinted with permission from ref. [5]. Copyright Year 2022, Isabel C.N. Sacco, (Physical Therapy, Speech, and Occupational Therapy Department, School of Medicine).

The database of EMG and corresponding GRF data was collected from the Akashi et al. [5] study. The study involved 45 adults with at least 5 years post-onset of Type 2 diabetes, divided into three groups: A control group (n = 16), a diabetic neuropathic group (n = 19), and a diabetic neuropathic group with a previous history of plantar ulceration (n = 10). However, the available dataset consisted of a total of 21 subjects from three different groups: control (n = 6), diabetic neuropathy (n = 6), and diabetic neuropathy with ulceration (n = 9). The data consisted of EMG of the right vastus lateralis (VL), gastrocnemius lateral (GL), and tibialis anterior (TA), and the 3-dimensional components of ground reaction force (GRFx, GRFy, GRFz) raw signals. In Figure 1, a sample of these EMG and GRF signals collected from the study [5] is shown. The data were sampled at 1000 Hz and collected according to the experimental procedure in [5]. The patients were examined for neuropathy using the Michigan neuropathy screening instrument (MNSI). The detailed protocol of the study has been explained by the Akashi et al. [5] study. Supplementary Table S1 summarizes the total data samples after segmentation and quality evaluation, where some examples refer to the segmented signals.

### 2.2. Signal Processing

The raw data were segmented using the Vertical ground reaction force (vGRF) and evaluated semi-manually at the preprocessing stage. The EMG signals were passed through a full-wave rectifier and band-pass Butterworth fourth-order Infinite Impulse Response (IIR) Zero-Phase filter. To meet the Nyquist certation, the filter cutoff frequency was selected between 25 and 499 Hz [38]. After that, the signal was passed through a digital notch filter to remove 60 Hz noise and its harmonics. The filters were designed using MATLAB R2020a software (MathWorks, Natick, MA, USA). After filtration, the EMG signals were segmented using vGRF to obtain the signal during the gait cycle and normalized by the mean of the EMG signal. The GRF signals had high-frequency noises, which could hamper the feature extraction process. Therefore, the GRF signals were filtered using a low pass Butterworth fourth-order Infinite Impulse Response (IIR) Zero-Phase Filter, implemented in MATLAB. The filter cutoff frequency was chosen to be 100 Hz. Unfitted data were removed from the 3 dimensions of the GRF signals after segmentation and normalized by signal mean.

### 2.3. Feature Extraction

#### 2.3.1. EMG Feature Extraction

Preprocessed EMG signals were used for feature extraction. In our previous study, we proposed two new time–domain features [37]: the log of the mean absolute value (LMAV) and the nonlinear scaled value (NSV). The details regarding these two features can be found in [37]. In this work, we showed that these time–domain features incorporated with another 17-time domain features from the existing literature exhibited better performance in the pattern recognition problem. So, we have used the proposed feature extraction scheme to identify diabetic neuropathic patients with and without a previous history of foot ulceration from EMG and GRF signals. In this research, we have used nineteen time–domain features previously reported by Islam et al. [37]. The extracted time–domain features were the log of the mean absolute value (LMAV), nonlinear scaled value (NSV), waveform length (WL), Wilson amplitude (WAMP), slope sign changes (SSC), number of zero crossings (ZC), mobility (MOB), complexity (COM), and skewness (SKW), and four autoregressive coefficients (AR1, AR2, AR3, and AR4), Moment (0th, 2nd, 4th, and 6th order) (m0-m6), Amplitude Change (1 and 2) (AC1, AC2).

#### 2.3.2. GRF Feature Extraction

A detailed investigation was done for extracting features from GRF signals. We extracted time (TD) [39], frequency (FD) [40,41], time–frequency (TFD) domain [42] features from the three GRF component signals. In this study, we extracted TFD features using discreet wavelet decomposition (DWT) [43,44] techniques. The DWT coefficient (detailed D1-D8, and their composed cD, Approximation A6-A8, and cA8) was then used to obtain band different TFD features. In this study, we extracted 50 TA, 24 FD, and 121 TFD features. In total, 195 features were calculated. The details of these features are indexed in Supplementary Table S2.

### 2.4. Feature Selection

A large number of attributes may confuse the model. Feature selections allow further dimensionality reduction. In this study, the forward feature selection approach was followed by adding one feature at a time and then checking the performance. To ensure using the best feature, the feature matrix was reordered according to feature importance. In this study, we mainly used filter type feature selection algorithms provided by MATLAB, which were Chi-square [45], minimum redundant maximum relevant (mrmr) [46], neighborhood component analysis (fscnca) [47], and Relieff [45] algorithms.

The Chi-square feature ranking method was first introduced to measure the goodness of fit by British statistician Karl Pearson. This algorithm is generally used in statistics to check the independence between two events [45]. For feature selection/feature ranking applications, we used it to test whether the occurrence of a specific term and the occurrence of a specific class were independent or not.

Minimum redundant maximum relevant (mrmr) is a method for feature selection [46]. The algorithm consists of two components: minimal redundancy and maximum relevance. The minimum redundancy selects the feature which has the least redundancy in the residual of features, while the maximal relevance selects the feature which has the strongest relevance to the target class.

Feature selection using neighborhood component analysis (fscnca) [47] learns feature weights with regularization to measure the average leave-one-out classification loss over training data. Based on the classification loss, the features’ importance was identified.

The Relieff feature ranking method uses a filter-based method, sensitive to feature interaction [45]. It calculates the score for each feature which is used to rank the features based on the score in ascending order. Relieff uses feature value difference between two nearest neighbors to generate those scores. If the difference is higher for the two-neighbor pair of the same class, the feature score decreases, indicating a less important feature.

### 2.5. Dimensionality Reduction

Higher feature dimensions can add computational complexity and time to the ML models. Dimension reduction is the process of finding independent and relevant dimensions or degrees of freedom in data [31]. There are many dimensionality reductions, such as Principal component analysis and locally linear embedding [47]. In this study, high correlation feature elimination was performed before feature ranking not to impact the feature ranking algorithm. The correlation matrix between features was calculated using pairwise linear correlation. A threshold of 0.9 was considered a highly collated feature, and one of them was dropped from the feature vector. This helped to reduce the dimension of the features. After removing highly correlated features, a dataset was prepared for three individual lower limb muscles (GL, VL, and TA) and three-dimensional GRF (GRFx, GRFy, and GRFz).

### 2.6. Machine Learning Models and Hyperparameter Tuning

For classification of the control, diabetic neuropathic (DN), diabetic neuropathic patients with a previous history of ulceration (DFU), we aimed to use machine learning algorithms. To develop the ML models, extracted features from EMG and GRF signals were used as input for the model. The output of the models was three classes: control, DN, and DFU. To find the best performing algorithm, we trained 8 different algorithms: Discriminant analysis classifier (DAC), Ensemble classification model (ECM), Kernel classification model (KCM), k-nearest neighbor model (KNN), Linear classification model (LCM), Naive Bayes classifier (NBC), Support vector machine classifier (SVM), and Binary decision classification tree (BDC) using the EMG, and GRF features. After identifying the best-performing algorithm, we optimized the algorithm to tune the hyperparameters of the algorithm by using Bayesian optimization. In the Bayesian optimization technique, a prediction model was developed to calculate the classification error by selecting hyperparameters and continue updating the values of the hyperparameters based on the error values until the minimum loss was achieved. That minimum classification error point was considered the best-tuned hyperparameters for that feature set. However, hyperparameter tuning is computationally complex and time-consuming. As our objective was to analyze the performance of ML models in different feature combinations for both EMG and GRF features, all 8 ML algorithms were not optimized. Only the best-performing algorithm was optimized. ML model development and tuning of the hyperparameters of the ML algorithm was implemented in MATLAB version 2020b (MathWorks, Natick, MA, USA).

### 2.7. ML Model Development

After finding the best performing model, it was used to classify the patients into three classes, control, DN, and DFU, using the EMG and GRF features’ data. ML models were trained in three different ways. The detailed training process is described below:

1. Single Channel: Initially, the ML model was trained for individual muscle or GRF component data. So, we trained ML models for three individual muscle features (GL, VL, and TA) and three GRF component features (GRFx, GRFy, and GRFz) separately and observed the classification performance.
2. Two Channel: Second, the ML model was trained with features combined from two muscles (GL and VL; GL and TA; and TA and VL) or two GRF components (GRFx and GRFy; GRFx and GRFz; and GRFy and GRFz).
3. Three Channel: Last, we observed the performance of the ML models with all three muscles (GL, VL, and TA) or three GRF (GRFx, GRFy, and GRFz) components combined.

The ML model was trained and validated with 5-fold cross-validation. In subject-wise validation, all the records of each subject were randomly assigned as a group to either the training set or the test set. A previous study showed that splitting the data into training and test sets in a record-wise fashion can lead to a massive underestimation of prediction error achieved by the machine learning algorithm [48]. Neto et al. [49] conducted a study by training different ML models using the various clinical datasets and showed a high degree of identity confounding for classifiers using record-wise data splits (splitting data into train and test set) and suggested the subject-wise data splits (where all records of a given participant are assigned either to the training or to the test set, but not to both) should be used in machine learning diagnostic applications.

**Figure 2:**
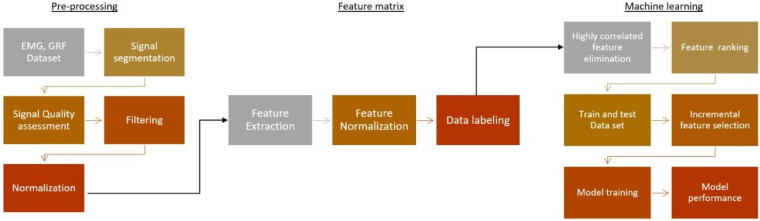
Flow chart of the data processing and ML model performance analysis for DN and DFU patients’ identification using EMG and GRF features.

So based on this observation, we used subject-wise data splits to train our ML model for classification. As the dataset was imbalanced, the training dataset was augmented using the Synthetic Minority Oversampling Technique (SMOTE) [50] technique to balance the dataset. Figure 2 illustrates the flow chart of the data processing and ML model performance analysis.

### 2.8. Performance Evaluation of ML Models

The performance of the ML algorithms was evaluated using different evaluation parameters, such as accuracy, sensitivity, specificity, precision, F score, Receiver operating characteristics curve (ROC), and Area under the curve (AUC). The model performance in classifying three experimental groups (control, DN, and DFU) was analyzed using the incremental feature search technique for all the ranked features in an ascending manner, starting from only the top 1 feature and then going on from the top 2 features and up. Here, we wanted to investigate the combined number of top-ranked features needed to obtain the best performance for respective cases. All processes were conducted for EMG and GRF data separately.

**Figure 3:**
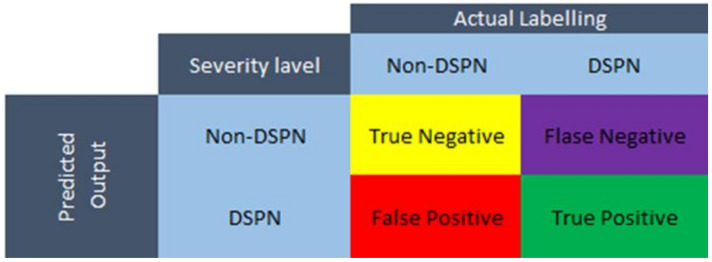
Confusion Matrix for two-class classification problem.

The target of this work was to find the best ML models for both EMG and GRF signals, respectively, based on the overall accuracy of the model. These parameters were calculated from a confusion matrix. A confusion matrix (CM) is one of the most used measures in classification problems since it offers a simple and intuitive visual representation of the performance of a given algorithm, in addition to being applicable to both binary and multi-class classification problems. It showcases the predicted class by a ML model versus the actual class of all the samples in the dataset and the number of accurately classified or misclassified samples. Figure 3 illustrates an example of a confusion matrix for a binary classification problem. Ideally, and in the case of 100% accuracy, all the off-diagonal elements must be zeros since diagonal blocks of the confusion matrix are considered true predictions. The confusion matrix has four matrices such as [51]:

True positive (TP): True DN patients
True negative (TN): True Non-DN
False-positive (FP): Non-DN patients, classified as DN patients.
False-negative (FN): DN patients, classified as non-DN patients.

Accuracy can be defined as the ratio between the correctly classified subjects to the total number of subjects, which provides an estimation of the overall performance of the model regardless of the class. This metric can be easily calculated using the previously described confusion matrix in Figure 3 by [51]:

(1) $$\mathrm{Accuracy}=\frac{\mathrm{Number}\;\mathrm{of}\;\mathrm{correct}\;\mathrm{prediction}}{\mathrm{Total}\;\mathrm{number}\;\mathrm{of}\;\mathrm{predicted}\;\mathrm{data}}\;=\frac{\mathrm{TP}+\mathrm{TN}}{\mathrm{TP}+\mathrm{TN}+\mathrm{FP}+\mathrm{FN}}$$

However, accuracy can be a misleading measure, especially when dealing with class-imbalanced datasets. For that reason, other performance metrics must be used to ensure reliability.

Sensitivity or recall is one of the most important measures, especially in these types of projects, since it describes the number of subjects that are correctly classified as positive among all the subjects that are labeled as positive. Typically, in multi-class classification problems, sensitivity is calculated for each class individually, and then they are combined either using macro- or micro-averaging techniques. For example, by referring to the previous confusion matrix, the sensitivity can be obtained as follows [51]:

(2) $$\mathrm{Sensitivity}=\frac{\mathrm{no}\;\mathrm{of}\;\mathrm{subject}\;\mathrm{accurately}\;\mathrm{classified}\;\mathrm{as}\;\mathrm{positive}}{\mathrm{total}\;\mathrm{no}\;\mathrm{of}\;\mathrm{subject}\;\mathrm{labeled}\;\mathrm{as}\;\mathrm{positive}}\;=\frac{\mathrm{TP}}{\mathrm{TP}+\mathrm{FN}}$$

Furthermore, precision is another measure of performance used when evaluating the performance of a ML model, which is the ratio of patients who were correctly identified as positive to the total number of positive predicted patients. It can be maximized by an impractical classifier, where they never predict the DSPN severity level to avoid false alarms. For example, Equation (3) can be used to calculate the precision [51].

(3) $$\;\mathrm{Precision}=\frac{\mathrm{no}\;\mathrm{of}\;\mathrm{subject}\;\mathrm{classified}\;\mathrm{correctly}\;\mathrm{as}\;\mathrm{Positive}}{\mathrm{no}\;\mathrm{of}\;\mathrm{subject}\;\mathrm{classified}\;\mathrm{as}\;\mathrm{Postive}\;}\;=\frac{\mathrm{TP}}{\mathrm{TP}+\mathrm{FP}}$$

Using the previous definitions of precision and recall, a new measure can be defined by combining both the values in one numeric metric called the F-measure, which is the harmonic mean of the sensitivity and precision. The F1 score considers both recall and precision as equally important measures. Usually, there is a trade-off between precision and recall; thus, the F1 score clarifies how balanced the model is between these two metrics. A higher score will show a balance between the two measures, while a significant difference between precision and recall will be penalized by the F-1 score. To calculate the F1 measure, the following formula is used [51]:

(4) $$F_{1}\;\mathrm{Score}=\frac{2\;\mathrm{TP}}{2\;\mathrm{TP}+\mathrm{FP}+\mathrm{FN}}\;$$

For the three-class problem, all the performance metrics were evaluated for each class, and the final value was obtained using macro averaging of all classes.

## 3. Results

The sociodemographic variables of the recruited subjects have been tabulated in Table 1.

### 3.1. Performance Evaluation

After training eight different ML algorithms, we found that the KNN algorithm outperformed the other algorithms using EMG and GRF data. The hyperparameters of the KNN algorithm were tuned using the Bayesian optimization technique. The optimized KNN model had a set Euclidean distance, number of neighbors set to 1, distance weighting function set to ‘squaredinverse’, which means the weight is equal to $\frac{1}{distance^{2}}$. We trained this optimized KNN algorithm for using different feature section techniques for EMG and GRF data, respectively. In the next section, we will highlight the best-performing results by the EMG and GRF feature sets.

### 3.2. EMG Signal Analysis

**Figure 4:**
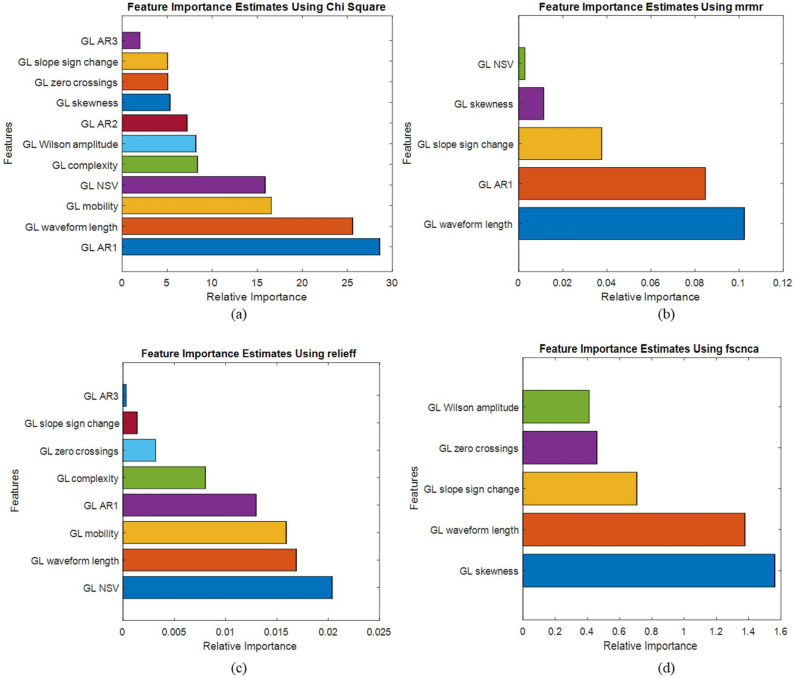
Top-ranked features from the GL muscle EMG signal by (**a**) Chi-Square, (**b**) mrmr, (**c**) Relieff, (**d**) fscnca feature selection techniques.

The EMG dataset initially consisted of 19 TD features for each muscle. Here, we implemented the study channel-wise, assuming each signal feature as channel data. So, we considered three lower limb muscles, GL, VL, and TA, in one channel analysis, with 19 features proposed by our previous work [37] individually. In two-channel analysis, we took a combination of two muscles features at a time. For three muscles, we have six different combinations of features, each having 38 features in each case, and for three channels, we considered all three muscle features with 57 features. The details of each channel analysis are added in Table 2. The EMG feature dataset was ranked based on its importance in identifying DN and DFU. Four different feature selection techniques were studied. After the features were ranked, the highly correlated features were removed in all cases. After removing highly correlated features from each channel, we had 11, 22, and 32 features in one, two, and three-channel analyses. The feature list after feature ranking and highly correlated feature removal can be found in Supplementary Tables S3–S9 for all channel analyses. The number of features before and after removing highly correlated features are listed in Table 2. In Figure 4, the ranked features by four different feature ranking techniques for GL muscles are shown. The dataset consisted of top-ranked features after removing highly correlated features, which were used to train the ML models, and their performance was analyzed. The ROC curve, as well as other evaluation matrices, were generated using top feature combinations starting from top 1 features, and so on, incrementally, to observe the performance of the model. In Table 3, Table 4 and Table 5, we summarized the best performance by the KNN classifier model in identifying control, DN, and DFU patients using EMG for one, two, and three-channel analysis, respectively.

**Figure 5:**
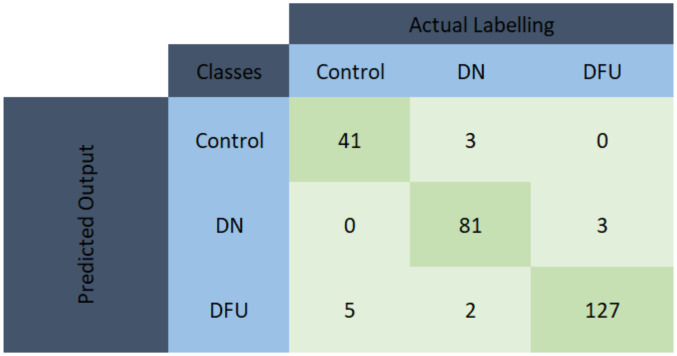
Confusion matrix for KNN model using the top 12 features extracted from GL and VL muscles EMG and feature ranked using the chi-square feature ranking technique.

**Figure 6:**
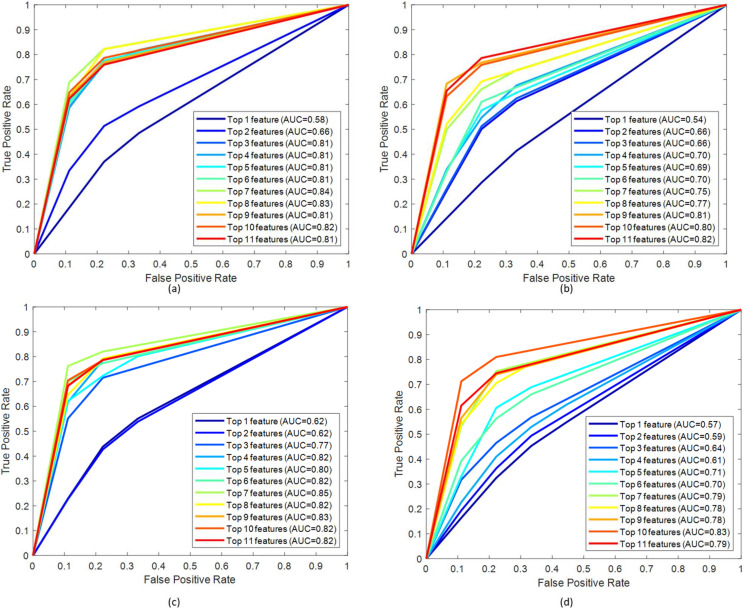
ROC curve for top-ranked features from the GL muscle EMG signal by (**a**) Chi-Square, (**b**) mrmr, (**c**) Relieff, (**d**) fscnca feature selection techniques.

From Table 3, it can be observed that the top 7 features from GL muscle EMG using the Relieff feature ranking technique exhibited better performance with an accuracy of 84.73% in comparison with the other two muscles. For the two-channel, in Table 4, the top 12 features from the combination of GL and VL muscle EMG using the Chi-Square feature ranking technique showed a 96.18% accuracy, outperforming the results using features from the other two muscle combinations. However, in the three-channel, the best performing model had an accuracy of 95.8% accuracy using the top 22 feature combination from all three lower limb muscles (Table 5). Among this three-channel analysis, the best performance was 96.18% using the features extracted from GL and VL muscles EMG and features ranked using the chi-square feature ranking technique. The related confusion matrix for this best-performing model is shown in Figure 5. The detailed performance of the three channels using EMG data is listed in Supplementary Tables S10–S16. Figure 6 illustrates the ROC curves using features from different feature selection techniques for GL muscle.

### 3.3. GRF Signal Analysis

From the GRF signal, 195 TD, FD, and TFD features were extracted for three-dimensional components. Similar to the EMG signal analysis, GRF signal analysis was also subdivided into three channels. In one-channel analysis, features for individual GRF components (GRFx, GRFy, and GRFz) were taken into consideration. For the two-channel, features from two GRF components were used. So, for three components, we had three combinations (GRFx-GRFy, GRFx-GRFz, GRFy-GRFz), and for the three-channel analysis, we considered features from all the GRF (GRFx-GRFy-GRFz) combined. The details of the channel analysis can be found in Table 6. The ranked features after removing highly correlated features for all channel analyses are being tabulated in Supplementary Tables S17–S23.

Similar to the analysis using EMG, four feature ranking techniques discussed above were also applied for GRF features for all channel analyses. After ranking the features, highly correlated features were removed, and the remaining features were used to train our optimized KNN model. In Table 6, the number of features before and after removing highly correlated features is shown.

**Figure 7:**
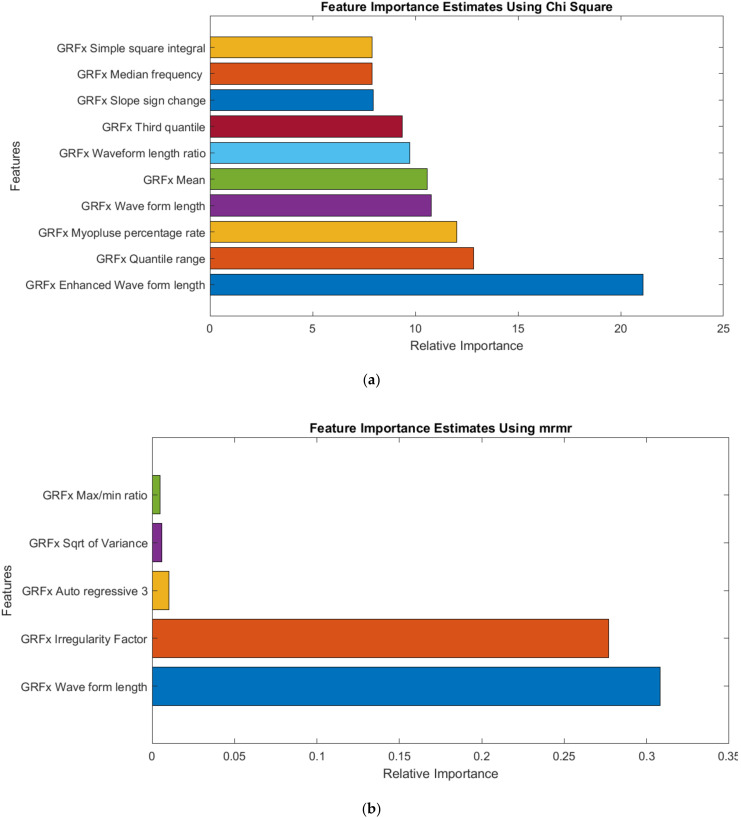

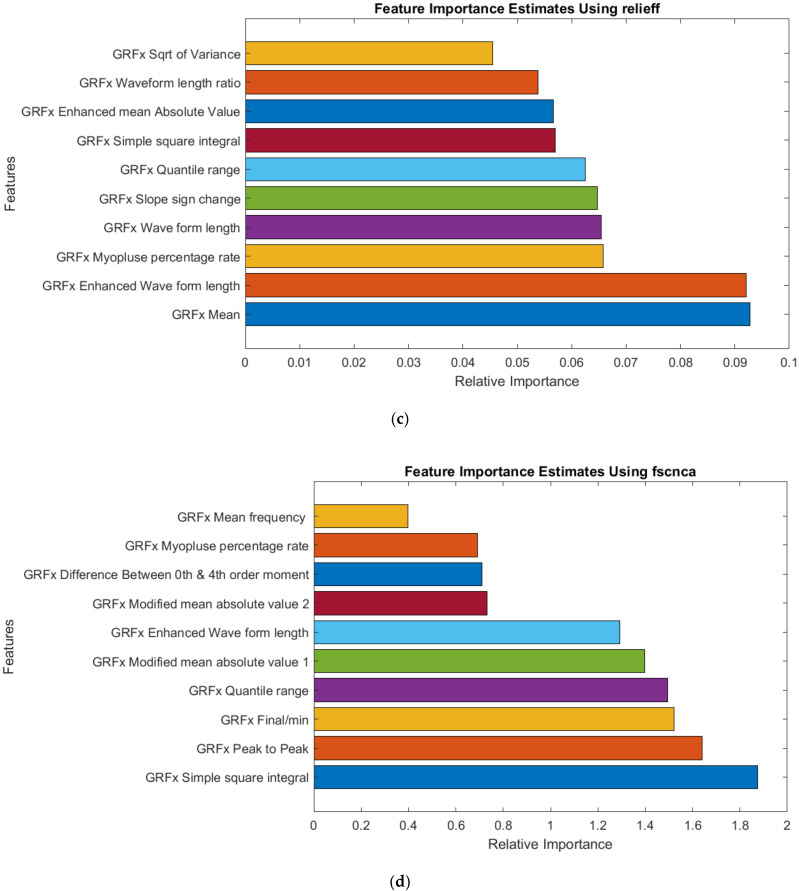
Top-ranked features from GRFx signal by (**a**) Chi-Square, (**b**) mrmr, (**c**) Relieff, (**d**) fscnca feature selection techniques.

Figure 7 illustrates the ranked features extracted from GRFx signals for the four different feature ranking techniques. The dataset consisted of top-ranked features after removing highly correlated features, which were used to train the ML models, and their performance was analyzed.

**Figure 8:**
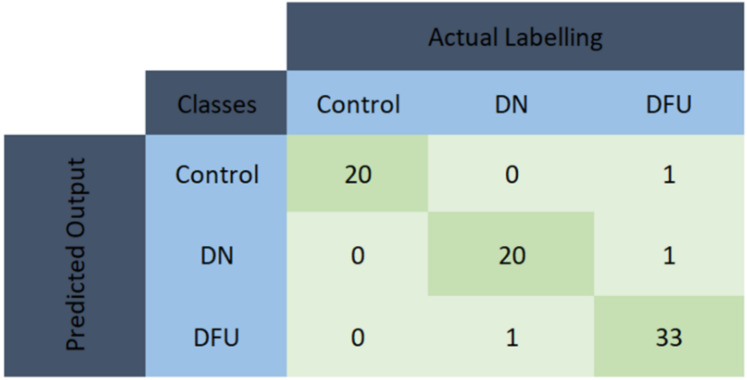
Confusion matrix for KNN model using the top 12 features extracted from GRFx and GRFz signals and feature ranked using the fscnca feature ranking technique.

**Figure 9:**
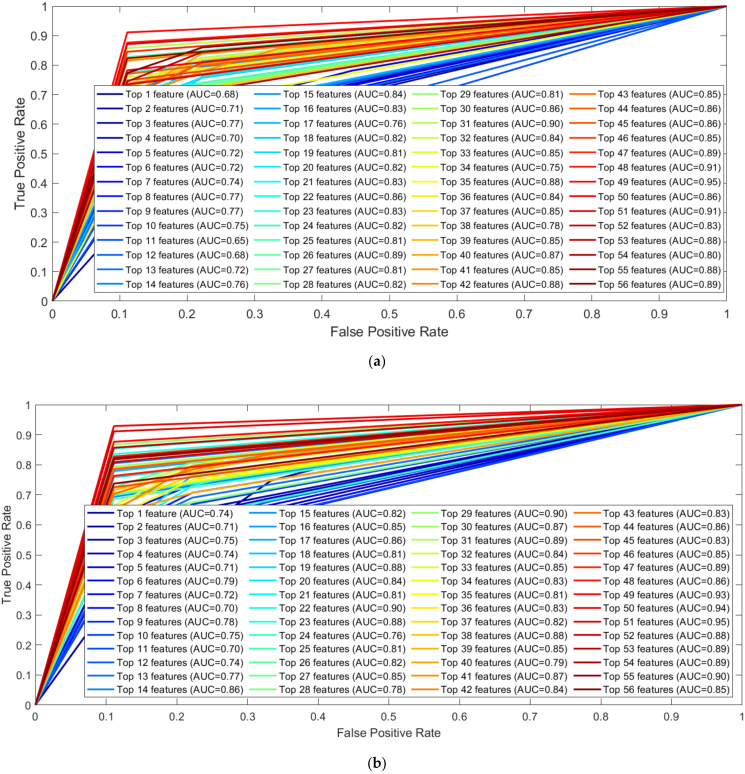

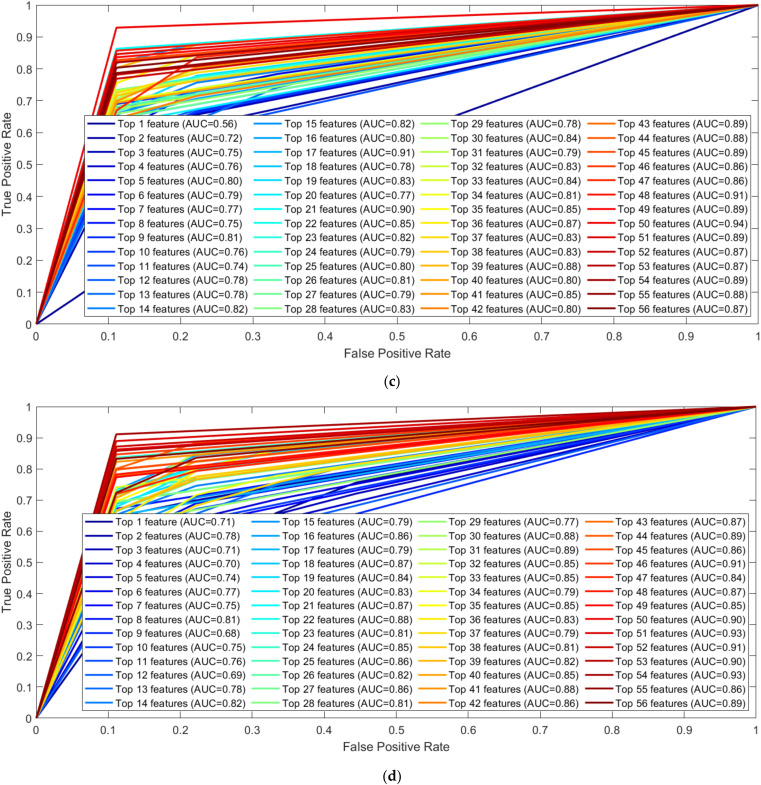
ROC curve for top-ranked features from the GRFx signal by (**a**) Chi-Square, (**b**) mrmr, (**c**) Relieff, (**d**) fscnca feature selection techniques.

From Table 7, it can be observed that the top 19 features from GRFx using the fscnca feature ranking technique, exhibited better performance with an accuracy of 98.68% in comparison with the other two muscles. For the two-channel, in Table 8, the top 7 features from a combination of GRFx and GRFz signals, using the fscnca feature ranking technique showed a 98.68% accuracy, outperforming the results using features from the other two muscle combinations. However, in three-channel, the best performing model had an accuracy of 98.68% using the top 24 features combination from all three lower limb muscles using the fscnca feature ranking technique (Table 9). Among these three-channel analyses, the best performance was achieved with 98.68% accuracy by using the top 7 features from the fscnca feature ranking technique, extracted from the combination of the GRFx-GRFz signal. The related confusion matrix for this best performing model is shown in Figure 8. The detailed performance of all channels using GRF data is listed in Supplementary Tables S24–S30. Figure 9 illustrates the ROC curves using features from different feature selection techniques for GRFx.

## 4. Discussion

Diabetic neuropathy (DN) has received the attention of researchers as one of the major complications for DM patients [52]. It is a long-term chronic complication in diabetic patients, which involves disfunction of electrophysiological activities in the human body, starting from the lower limb. Literature showed that DN patients exhibited biomechanical changes in the gait cycle, effecting an alteration in plantar pressure, kinematic patterns, ground reaction forces, muscle activation, sensory loss, slower walking speed, changes in gait velocity, increased risk of falling, and many other lower limb and gait complications [6,13,17,21,22,53]. With long-term DN, other serious foot complications start to arise. Planter foot ulceration is one of the major complications of long-term DN, an indication of worsening DN [5,15,22,23,54], which is one of the major causes of lower limb amputations [54,55,56]. Thus, prediction and on-time treatment of diabetic foot ulcers (DFU) are of great importance for improving and maintaining patients’ quality of life and avoiding the consequent socioeconomic burden of amputation [57].

Akashi et al. [5] showed that DN patients with (DFU) and without a history of plantar ulceration had a delayed activation peak occurrence in VL and GL muscles and a decrease in the second peak of vertical ground reaction force compared with the control group during gait. However, they did not find any difference in TA muscle time for activation peak of magnitude. Sacco and Amadio [20] reported delayed muscle activation in VL muscles during treadmill gait in DN patients, in agreement with the Akashi et al. study [5], where they suggested that a delayed muscle activation peak can be an indicator of worsening DN, hence, DFU. However, another study by Sacco and Amadio [20] and Abboud et al. [22] reported that TA muscles also exhibited decreased muscle magnitude and delayed activation peak, which contradicts the results from Akashi et al. [5]. Similar to this, many studies have been conducted, which have reported contradictory results [5,6,7,9,11,12,13,14,15,16,22,23,25,57]. 

In such a scenario, only relying on EMG and GRF signal analysis to find biomechanical changes in DN patients cannot be reliable. Depending on the variability of the recruited population for these studies, the biomechanical changes in muscle EMG and GRF during gait can be varied among the same experimental groups, depending on the patient’s ability to adapt to the changes in gait [6]. Patients did not exhibit any patterned delay in the muscle activation functions, which can cause unreliable diagnoses. With the help of Machine Learning (ML), EMG and GRF signal analysis can be made more robust and overcome the variability in the research regarding the patient’s identification.

Even though many researchers reported finding a relationship in the biomechanical changes in DN and DFU during gait, the use of machine learning techniques in this domain is quite new. ML learning-based applications using EMG have received the attention of researchers for various applications, such as disease diagnosis, prosthetics design, myoelectric controllers, and security systems. Despite having so much potential, ML has not been widely studied in clinical biomechanics related to DN and DFU. In the literature, very few studies have been conducted to identify DN and DFU patients with the help of ML using biomechanics parameters, such as EMG and GRF. In Sawacha et al.’s [23] study, the researchers used a K-means clustering classification system to classify the subjects’ gait patterns among DN and Non-DN groups through the analysis of their ground reaction forces, joints, and segments (trunk, hip, knee, ankle) angles, and moments. However, as the K-means cluster technique is sensitive to the variation in the gait trails, the reproducibility of this study is questionable. Fahmida et al. [9] proposed an adaptive neuro-fuzzy inference system-based classifier for diabetic sensorimotor polyneuropathy (DSPN) severity classification by using features from three lower limb muscles (TA, VL, GM) EMG during gait. In their study, they used only two EMG features based on the existing literature time for activation peak and peak magnitude during gait, with the progression of DSPN severity. They trained two different models. In one model, they used only the peak magnitudes from three different lower limb muscles, and in another model, they considered both the features of all three muscles. They reported 96% and 80% accuracy for both models, respectively. From this aspect, we investigated a ML-based classification system for identifying DN and DFU patients by using biomechanical parameters from EMG and GRF during gait.

As per our knowledge, this is the first ML-based work to classify DN and DUF patients using EMG and GRF data during gait. In this study, we collected data from the Akashi et al. [5] study, where we had a total of 21 patients’ data from three experimental classes: control (n = 6), DN (n = 6), and DFU (n = 9), and observed the EMG and GRF characteristics during gait. This study discussed detailed preprocessing of the signals, extracting and identifying important features from EMG and GRF signals. Eight different conventional ML algorithms were trained and the KNN algorithm outperformed for identifying DN and DFU for both features from EMG and GRF signals. Hyperparameters of the KNN algorithm were optimized using the Bayesian Optimization technique.

This study was conducted for three sub-studies for EMG and GRF, respectively. In one-channel analysis, we considered the features from only one lower limb muscle or GRF component. In the two-channel, we considered the feature combinations of two muscles or GRF components, and in the three-channel, all the muscles or GRF component features were considered. Depending on the channel, the number of features varied, fed to the ML model for training. In this study, we trained our ML model in all possible ways to find the best-performing features.

This study dealt with a three-class problem, where we considered three classes: control, DN, and DFU. The evaluation matrixes were calculated depending on the accurate identification of the correct class. For EMG analysis, we found the best accuracy of 96.18% using the top 22 features from the chi-square feature ranking technique in the two-channel analysis of GL-VL. In the GRF analysis, the model showed 98.68% accuracy by using the top 7 features from the fscnca feature ranking technique from the combination of the GRFx-GRFz signal. In this study, before training our ML models, we preprocessed the dataset in a very detailed way involving removing noise and proper filtration, normalization, and segmentation of the signals, which created this dataset without bias or overfitting issues. Because of our proper data preprocessing, our work obtained very good performance with EMG and GRF data analysis in identifying DN and DFU. This indicates that analysis for sensitive biomechanical data required very detailed preprocessing before being used for training ML models. With the help of proper preprocessing, higher accuracy can be achieved. This is one of the major contributions of this study. Another major contribution of this study is that using the new feature extraction scheme for EMG signals, it helped us extract important features.

In this research, our goal was to find the best-performing ML models using features from EMG and GRF signals individually during gate. In this research, we tried to identify DN and DFU patients from the biomechanical signals such as EMG and GRF with the help of ML. As per our knowledge, this is the first study where ML application has been studied for DN and DFU identification from biomechanical signals during gait. This study demonstrated that both EMG and GRF signals can be used to identify the patient’s class with higher accuracy, with the help of proper preprocessing of the signals, extracting and identifying important features, and optimizing the ML algorithm. However, a few limitations need to be mentioned. One is the small sample size of the study. As the data were collected from the Akashi et al. study [5], there were only 21 patients’ data. Even though we used the subject-wise data split technique and SMOTE data augmentation technique to overcome the imbalance and small dataset for ML model training, there is still a chance of variability in the results when a large number of variations are being exposed. Another limitation of this study is that we only considered the control, DN, and DFU patient groups as available in the study [5]. We had no diabetes patients without DN in this study. In the future, the effect on diabetes patients without DN should be included in our analysis.

Apart from the mentioned limitation, this study showed a potential use of ML in the biomechanical domain for DN and DFU identification. If proper preprocessing with strategic techniques is applied to extract important features from biomechanics parameters such as EMG and GRF, these can be a potential diagnostic tool for DN and DFU. With valid data, an early indication of DFU can be made from the EMG and GRF with the help of machine learning. This is a very initial study in this domain. For years researchers have had contradictory results with biomechanical changes related to DN. Our work highlights the potential of ML for DN-related complications by using biomechanical parameters such as EMG and GRF. In the future, we can add a prediction system in ML-based DFU prediction from EMG and GRF data which will be able to predict patients’ future conditions based on their biomechanical changes and will help health professionals enhance the healthcare of diabetic patients in relation to identifying high-risk individuals and provide the proper treatment and rehabilitation.

## 5. Conclusions

In this paper, we applied ML models for identifying DN and DFU using EMG and GRF signals, which is the first work, as per our knowledge, in this domain. In this work, we discussed detailed signal processing, feature extraction, feature ranking, and feature dimension reduction techniques to identify the most important features for DN and DFU identification. This research considered EMG signals from three different lower limb muscles and corresponding 3-dimensional GRF signals. Previously in the literature, all the biomechanical changes due to DN and DFU have been analyzed manually. This is the first work where the authors have shown that, with the help of ML, DN and DFU patients can be identified with higher accuracy using features from the EMG and GRF signals individually. This research proposed the two best models for EMG and GRF, respectively. For EMG analysis, we found the best accuracy of 96.18% using the top 22 features from the chi-square feature ranking technique in the two-channel analysis of GL-VL. In the GRF analysis, the model showed 98.68% accuracy by using the top 7 features from the fscnca feature ranking technique from the combination of the GRFx-GRFz signal. In conclusion, our study has shown a potential solution for ML application in DN and DFU patient identification using EMG and GRF parameters. With careful signal preprocessing with strategic feature extraction from the biomechanical parameters, optimization of the ML model can be a potential solution in the diagnosis and stratification of DN and DFU patients from the EMG and GRF signals.

## Figures and Tables

**Table 1:** Sociodemographic characteristics of the patients. Reprinted with permission from ref. [5]. Copyright Year 2022, ‪Isabel C.N. Sacco, (Physical Therapy, Speech, and Occupational Therapy Department, School of Medicine).

	Control Group (n = 6)	Diabetic Group (n = 6)	Ulcer Diabetic Group (n = 9)	*p*
Age(years)	52.2 ± 6.9	56.0 ± 8.5	55.6 ± 6.0	0.7 ^a^
Gender(male/female)	3/3	2/4	1/8	-
Weight (Kg)	61.7 ± 5.3	66.7 ± 12.2	83.4 ± 10.5	<0.001 ^a^
Height (m)	1.7 ± 0.09	1.6 ± 0.07	1.7 ± 0.07	0.008 ^a^
BMI (kg/m^2^)	22.1 ± 1.7 ^c^	25.1 ± 4.0	27.8 ± 3.4	0.029 ^a^
Time of DM (years)	-	15.2 ± 6.8	14.3 ± 5.8	0.80 ^a^
Glycemia(mg/dL)	-	142 ± 61.8	188.6 ± 61.2	0.17 ^a^
MNSI Questionnaire ^d^	-	7	7.5	0.72 ^b^
MNSI Clinical Examination ^d^	-	2.5	4	0.12 ^b^

^a^ One-Way ANOVA; ^b^ Mann–Whitney test; ^c^ The significantly different group; ^d^ Median.

**Table 2:** Number of features extracted from lower limb muscle EMG for a different combination.

Combination	Muscles	No. of Features	After Removing Highly Correlated Features
One Channel	GL	19	11
	VL		
	TA		
Two Channel	GL-VL	38	24
	GL-TA		22
	TA-VL		22
Three Channel	GL-VL-Ta	57	34

**Table 3:** Evaluation matrix for one-channel analysis for EMG data using different feature selection techniques.

Muscle	Feature Ranking Technique	Features	Accuracy (%)	Precision (%)	Sensitivity (%)	F1 Score (%)	AUC
GL	Chi-Square	Top 8	81.30	82.22	81.30	81.54	0.83
	mrmr	Top 11	80.92	81.59	80.92	81.11	0.82
	Relieff	Top 7	84.73	84.89	84.73	84.76	0.85
	fscnca	Top 10	81.30	82.27	81.30	81.54	0.83
TA	Chi-Square	Top 8	80.53	80.91	80.53	80.59	0.82
	mrmr	Top 9	84.35	85.06	84.35	84.48	0.87
	Relieff	Top 9	81.30	81.58	81.30	81.40	0.82
	fscnca	Top 11	83.59	83.93	83.59	83.67	0.86
VL	Chi-Square	Top 10	74.81	75.55	74.81	75.09	0.80
	mrmr	Top 11	75.95	76.77	75.95	76.24	0.82
	Relieff	Top 8	77.10	77.96	77.10	77.39	0.83
	fscnca	Top 11	76.72	77.25	76.72	76.84	0.83

**Table 4:** Evaluation matrix for two-channel analysis for EMG data using different feature selection techniques.

Muscles	Feature Ranking Technique	Features	Accuracy (%)	Precision (%)	Sensitivity (%)	F1 Score (%)	AUC
GL-TA	Chi-Square	Top 15	95.80	96.04	95.80	95.84	0.96
	mrmr	Top 17	94.27	94.38	94.27	94.30	0.95
	Relieff	Top 17	95.04	95.16	95.04	95.05	0.96
	fscnca	Top 22	93.51	93.67	93.51	93.55	0.94
TA-VL	Chi-Square	Top 18	92.37	92.71	92.37	92.44	0.94
	mrmr	Top 13	91.98	92.07	91.98	92.01	0.91
	Relieff	Top 17	92.75	93.03	92.75	92.82	0.93
	fscnca	Top 20	93.51	93.80	93.51	93.58	0.94
GL-VL	Chi-Square	Top 12	96.18	96.25	96.18	96.20	0.97
	mrmr	Top 14	93.51	93.59	93.51	93.52	0.95
	Relieff	Top 16	95.80	95.84	95.80	95.81	0.97
	fscnca	Top 21	92.75	93.12	92.75	92.82	0.96

**Table 5:** Evaluation matrix for three-channel analysis for EMG data using different feature selection techniques.

Muscles	Feature Ranking Technique	Features	Accuracy (%)	Precision (%)	Sensitivity (%)	F1 Score (%)	AUC
**GL-TA-VL**	Chi-Square	Top 27	95.80	95.86	95.80	95.78	0.99
	mrmr	Top 38	95.42	95.68	95.42	95.41	0.98
	Relieff	Top 23	95.80	95.86	95.80	95.80	0.97
	fscnca	Top 36	95.04	95.04	95.04	95.03	0.98

**Table 6:** Number of features extracted from GRF for a different combination.

Combination	Muscles	No. of Features	After Removing Highly Correlated Features
One Channel	GRFx	195	56
	GRFy		50
	GRFz		37
Two Channel	GRFx-GRFy	390	102
	GRFx-GRFz		87
	GRFy-GRFz		86
Three Channel	GRFx-GRFy-GRFz	585	129

**Table 7:** Evaluation matrix for one-channel analysis for GRF data using different feature selection techniques.

GRF Component	Feature Ranking Technique	Features	Accuracy (%)	Precision (%)	Sensitivity (%)	F1 Score (%)	AUC
GRFx	Chi-Square	Top 38	97.37	97.41	97.37	97.36	0.98
	mrmr	Top 50	96.05	96.28	96.05	96.05	0.98
	Relieff	Top 14	97.37	97.41	97.37	97.36	0.98
	fscnca	Top 19	98.68	98.72	98.68	98.68	1.00
GRFy	Chi-Square	Top 15	92.11	92.28	92.11	92.11	0.95
	mrmr	Top 34	86.84	87.26	86.84	86.78	0.89
	Relieff	Top 35	92.11	92.27	92.11	92.12	0.95
	fscnca	Top 21	93.42	94.32	93.42	93.40	0.96
GRFz	Chi-Square	Top 24	94.74	94.75	94.74	94.72	0.95
	mrmr	Top 18	93.42	93.47	93.42	93.42	0.94
	Relieff	Top 32	93.42	93.71	93.42	93.41	0.93
	fscnca	Top 13	93.42	93.65	93.42	93.45	0.95

**Table 8:** Evaluation matrix for two-channel analysis for GRF data using different feature selection techniques.

GRF Component	Feature Ranking Technique	Features	Accuracy (%)	Precision (%)	Sensitivity (%)	F1 Score (%)	AUC
GRFy-GRFz	Chi-Square	Top 3	94.74	94.83	94.74	94.67	0.92
	mrmr	Top 42	96.05	96.05	96.05	96.05	0.97
	Relieff	Top 45	94.74	94.82	94.74	94.75	0.96
	fscnca	Top 20	98.68	98.72	98.68	98.68	1.00
GRFx-GRFz	Chi-Square	Top 24	94.74	94.93	94.74	94.75	0.96
	mrmr	Top 19	97.37	97.43	97.37	97.37	0.99
	Relieff	Top 41	98.68	98.72	98.68	98.68	1.00
	fscnca	Top 7	98.68	98.72	98.68	98.68	0.98
GRFx-GRFy	Chi-Square	Top 17	96.05	96.24	96.05	96.07	0.98
	mrmr	Top 87	97.37	97.43	97.37	97.37	0.97
	Relieff	Top 17	96.05	96.07	96.05	96.04	0.99
	fscnca	Top 24	98.68	98.72	98.68	98.68	1.00

**Table 9:** Evaluation matrix for three-channel analysis for GRF data using different feature selection techniques.

GRF Component	Feature Ranking Technique	Accuracy (%)	Precision (%)	Sensitivity (%)	F1 Score (%)	AUC	Accuracy (%)
GRFx-GRFy-GRFz	Chi-Square	Top 111	97.37	97.41	97.37	97.36	1.00
	mrmr	Top 64	98.68	98.72	98.68	98.68	1.00
	Relieff	Top 106	98.68	98.72	98.68	98.68	1.00
	fscnca	Top 21	98.68	98.72	98.68	98.68	1.00

## Data Availability

The dataset used in this study is not publicly available.

## Supplementary Materials

The following supporting information can be downloaded at: https://www.mdpi.com/article/10.3390/s22093507/s1, Table S1: Dataset summary after segmentation; Table S2: Feature vectors summary for GRF; Table S3: Feature list from different selection techniques from GL muscle EMG; Table S4: Feature list from different selection techniques from VL muscle EMG; Table S5: Feature list from different selection techniques from TA muscle EMG; Table S6: Feature list from different selection techniques from GL-VL muscles EMG; Table S7: Feature list from different selection techniques from GL-TA muscles EMG; Table S8: Feature list from different selection techniques from TA-VL muscles EMG; Table S9: Feature list from different selection techniques from GL-VL-TA muscles EMG; Table S10: Performance evaluation matrix using features from GL muscle EMG for different feature selection techniques; Table S11: Performance evaluation matrix using features from VL muscle EMG for different feature selection techniques; Table S12: Performance evaluation matrix using features from TA muscle EMG for different feature selection techniques; Table S13: Performance evaluation matrix using features from GL and TA muscles EMG for different feature selection techniques; Table S14: Performance evaluation matrix using features from GL and VL muscles EMG for different feature selection techniques; Table S15: Performance evaluation matrix using features from TA and VL muscles EMG for different feature selection techniques; Table S16: Performance evaluation matrix using features from GL, VL, and TA muscles EMG for different feature selection techniques; Table S17: Feature list from different selection techniques from GRFx; Table S18: Feature list from different selection techniques from GRFy; Table S19: Feature list from different selection techniques from GRFz; Table S20: Feature list from different selection techniques from GRFx-GRFy; Table S21: Feature list from different selection techniques from GRFx-GRFz; Table S22: Feature list from different Selection Techniques from GRFy-GRFz; Table S23: Feature list from different selection techniques from GRFx-GRFy-GRFz; Table S24: Performance evaluation matrix using features from GRFx for different feature selection techniques; Table S25: Performance evaluation matrix using features from GRFy muscle EMG for different feature selection techniques; Table S26: Performance evaluation matrix using features from GRFz muscle EMG for different feature selection techniques; Table S27: Performance evaluation matrix using features from GRFx and GRFy muscles EMG for different feature selection techniques; Table S28: Performance evaluation matrix using features from GRFx and GRFz muscles EMG for different feature selection techniques; Table S29: Performance evaluation matrix using features from GRFy and GRFz muscles EMG for different feature selection techniques; Table S30: Performance evaluation matrix using features from GRFx, GRFy and GRFz muscles EMG for different feature selection techniques.
