# Supplementary material for: Machine Learning-Based Diabetic Neuropathy and Previous Foot Ulceration Patients Detection Using Electromyography and Ground Reaction Forces during Gait
Source: Sensors (Basel). 2022 May 5;22(9):3507. doi: 10.3390/s22093507 (PMC9100406; doi:10.3390/s22093507)
Supplement: Supplementary file 1 [file sensors-22-03507-s001.zip › sensors-1650068-supplementary.pdf]

sensors

supplementary

Machine Learning-Based Diabetic Neuropathy and Previous Foot Ulceration Patients Detection Using Electromyography and Ground Reaction Forces during Gait

Fahmida Haque [1], Mamun Bin Ibne Reaz [1,*], Muhammad Enamul Hoque Chowdhury [2,*], Maymouna Ezeddin [2], Serkan Kiranyaz [2], Mohammed Alhatou [3,4], Sawal Hamid Md Ali [1], Ahmad Ashrif A Bakar [1] and Geetika Srivastava [5]

[1] Department of Electrical, Electronic and System Engineering, Universiti Kebangsaan Malaysia, Bangi 43600, Malaysia; P97694@siswa.ukm.edu.my (F.H.); sawal@ukm.edu.my (S.H.M.A.); ashrif@ukm.edu.my (A.A.A.B.)
[2] Department of Electrical Engineering, Qatar University, Doha 2713, Qatar; me1511677@qu.edu.qa (M.E.); mkiranyaz@qu.edu.qa (S.K.)
[3] Neuromuscular Division, Hamad General Hospital, Doha 3050, Qatar; malhatou@hamad.qa
[4] Department of Neurology, Al khor Hospital Doha 3050, Qatar
[5] Department of Physics and Electronics, Dr. Ram Manohar Lohia Avadh University, Faizabad, Uttar Pradesh 224001India; gsrivastava@rmlau.ac.in
* Correspondence: mamun@ukm.edu.my (M.B.I.R.); mchowdhury@qu.edu.qa (M.E.H.C.)

Table S1. Data set summary after segmentation.

Class name	Num. of subjects	Num. of trials	Num. GRF examples	Num. EMG examples		
				LG	TA	VL
**Control**	**6**	**21**	21	82	34	34
Diabetics	6	21	21	84	84	84
Ulcer	9	34	34	132	129	132
Total	21	76	76	298	247	250

Table S2. Feature vectors summary for GRF.

TD features (no of Feature 50)	1	'Enhanced mean Absolute Value'
	2	'Enhanced Wave form length'
	3	'Mean absolute Value'
	4	'Wave form length'
	5	'Zero crossing'
	6	‘Slope sign change'
	7	'RMS'
	8	'Average amplitude change'
	9	Difference absolute standard deviation '
	10	'Log detector'
	11	'Modified mean absolute value 1'
	12	'Modified mean absolute value 2'
	13	'Myopluse percentage rate'
	14	'Simple square integral'

	15	'Variance'
	16	'Willison amplitude'
	17	'Maximum Fractal length'
	18	'Average power'
	19	'Standard Deviation'
	20	'Peak to Peak'
	21	'Shape factor'
	22	'Total Energy'
	23	'Integrated absolute value'
	24	Sqrt of Variance
	25	auto regressive 1'
	26	'Auto regressive 2'
	27	'Auto regressive 3'
	28	'Auto regressive 4'
	29	'Estimate (signal entropy)'
	30	'Sigma(signal entropy)'
	31	Descriptor SE Lower Bound
	32	Descriptor SE Upper Bound
	33	Descriptor SE NCELL
	34	Zero order moment
	35	Difference Between 0th & 2nd order moment
	36	Difference Between 0th & 4th order moment
	37	Sparseness
	38	Irregularity Factor
	39	Waveform length ratio
	40	'Signal Kurtosis'
	41	'Coefficient of variation'
	42	'Signal skewness'
	43	'Mean'
	44	'First quantile'
	45	'Third quantile'
	46	'Quantile range'
	47	'Max/min ratio'
	48	'Final/min'
	49	'Time to peak'
	50	'Peak'
Power spectral FD features (no of Feature 24)	51	'Mean frequency '
	52	'Median frequency '
	53	'Spectral entropy'
	54	'Max frequency'
	55	'Max value '
	56	'Max ratio'
	57	'Powe spectral (PS) skewness'
	58	'PS kurtosis'

	59	'PS mean frequency'
	60	'PS median frequency'
	61	'Signal to motion artifact ratio'
	62	'Max to min drop of PS'
	63	'Signal to noise ratio '
	64	'Spectral deformation '
	65	'Spectral centroid'
	66	'Spectral crest'
	67	'Spectral decrease'
	68	'Spectral flatness'
	69	'Spectral flux'
	70	'Spectral kurtosis'
	71	'Spectral roll off point '
	72	'Spectral skewness'
	73	'Spectral slope'
	74	'Spectral spread'
TDF features using DWT techniques (no of Feature 121)	75	'Band power of cD1'
	76	'Band power of cD2'
	77	'Band power of cD3'
	78	'Band power of cD4'
	79	'Band power of cD5'
	80	'Band power of cD6'
	81	'Band power of cD7'
	82	'Band power of cD8'
	83	'Band power of cA8'
	84	'Band power of D1'
	85	'Band power of D2'
	86	'Band power of D3'
	87	'Band power of D4'
	88	'Band power of D5'
	89	'Band power of D6'
	90	'Band power of D7'
	91	'Band power of D8'
	92	'Band power of A6'
	93	'Band power of A8'
	94	'Mean absolute value cD1'
	95	'Mean absolute value cD2'
	96	'Mean absolute value cD3'
	97	'Mean absolute value cD4'
	98	'Mean absolute value cD5'
	99	'Mean absolute value cD6'
	100	'Mean absolute value cD7'
	101	'Mean absolute value cD8'
	102	'Mean absolute value cA8'

103	'Mean absolute value D1'
104	'Mean absolute value D2'
105	'Mean absolute value D3'
106	'Mean absolute value D4'
107	'Mean absolute value D5'
108	'Mean absolute value D6'
109	'Mean absolute Value D7'
110	'Mean absolute value D8'
111	'Mean absolute value A6'
112	'Mean absolute value A8'
113	'Waveform length cD1'
114	'Waveform length cD2'
115	'Waveform length cD3'
116	'Waveform length cD4'
117	'Waveform length cD5'
118	'Waveform length cD6'
119	'Waveform length cD7'
120	'Waveform length cD8'
121	'Waveform length cA8'
122	'Waveform length D1'
123	'Waveform length D2'
124	'Waveform length D3'
125	'Waveform length D4'
126	'Waveform length D5'
127	'Waveform length D6'
128	'Waveform length D7'
129	'Waveform length D8'
130	'Waveform length A6'
131	'Waveform length A8'
132	'RMS cD1'
133	'RMS cD2'
134	'RMS cD3'
135	'RMS cD4'
136	'RMS cD5'
137	'RMS cD6'
138	'RMS cD7'
139	'RMS cD8'
140	'RMS cA8'
141	'RMS D1'
142	'RMS D2'
143	'RMS D3'
144	'RMS D4'
145	'RMS D5'
146	'RMS D6'
147	'RMS D7'

148	'RMS D8'
149	'RMS A6'
150	'RMS A8'
151	'Standard Deviation cD1'
152	'Standard Deviation cD2'
153	'Standard Deviation cD3'
154	'Standard Deviation cD4'
155	'Standard Deviation cD5'
156	'Standard Deviation cD6'
157	'Standard Deviation cD7'
158	'Standard Deviation cD8'
159	'Standard Deviation cA8'
160	'Standard Deviation D1'
161	'Standard Deviation D2'
162	'Standard Deviation D3'
163	'Standard Deviation D4'
164	'Standard Deviation D5'
165	'Standard Deviation D6'
166	'Standard Deviation D7'
167	'Standard Deviation D8'
168	'Standard Deviation A6'
169	'Standard Deviation A8'
170	'Max Fractal length cD1'
171	'Max Fractal length cD2'
172	'Max Fractal length cD3'
173	'Max Fractal length cD4'
174	'Max Fractal length cD5'
175	'Max Fractal length cD6'
176	'Max Fractal length cD7'
177	'Max Fractal length cD8'
178	'Max Fractal length cA8'
179	'Max Fractal length D1'
180	'Max Fractal length D2'
181	'Max Fractal length D3'
182	'Max Fractal length D4'
183	'Max Fractal length D5'
184	'Max Fractal length D6'
185	'Max Fractal length D7'
186	'Max Fractal length D8'
187	'Max Fractal length A6'
188	'Max Fractal length A8'
189	'Wavelet entropy level 8'
190	'Wavelet entropy level 6'
191	'Wavelet entropy level 4'
192	First peak

193	Second peak
194	LOC of first peak
195	LOC of second peak

Table S3. Feature list from different Selection Techniques from GL muscle EMG.

Ranking	Chi Square	mrmr	relieff	fscnca
1	'GL AR1'	'GL waveform length'	'GL NSV'	'GL skewness'
2	'GL waveform length'	'GL AR1'	'GL waveform length'	'GL waveform length'
3	'GL mobility'	'GL slope sign change'	'GL mobility'	'GL slope sign change'
4	'GL NSV'	'GL skewness'	'GL AR1'	'GL zero crossings'
5	'GL complexity'	'GL NSV'	'GL complexity'	'GL Wilson amplitude'
6	'GL Wilson amplitude'	'GL zero crossings'	'GL zero crossings'	'GL AR2'
7	'GL AR2'	'GL Wilson amplitude'	'GL slope sign change'	'GL mobility'
8	'GL skewness'	'GL mobility'	'GL AR3'	'GL AR1'
9	'GL zero crossings'	'GL complexity'	'GL Wilson amplitude'	'GL AR3'
10	'GL slope sign change'	'GL AR2'	'GL AR2'	'GL complexity'
11	'GL AR3'	'GL AR3'	'GL skewness'	'GL NSV'

Table S4. Feature list from different Selection Techniques from VL muscle EMG.

Ranking	Chi Square	mrmr	relieff	fscnca
1	'TA NSV'	'TA NSV'	'TA NSV'	'TA waveform length'
2	'TA slope sign change'	'TA AR3'	'TA waveform length'	'TA zero crossings'
3	'TA waveform length'	'TA AR1'	'TA Wilson amplitude'	'TA Wilson amplitude'
4	'TA zero crossings'	'TA slope sign change'	'TA AR1'	'TA slope sign change'
5	'TA Wilson amplitude'	'TA waveform length'	'TA mobility'	'TA mobility'
6	'TA skewness'	'TA complexity'	'TA slope sign change'	'TA AR2'
7	'TA AR1'	'TA mobility'	'TA complexity'	'TA AR3'
8	'TA AR3'	'TA zero crossings'	'TA skewness'	'TA NSV'
9	'TA AR2'	'TA skewness'	'TA zero crossings'	'TA AR1'
10	'TA mobility'	'TA Wilson amplitude'	'TA AR2'	'TA complexity'
11	'TA complexity'	'TA AR2'	'TA AR3'	'TA skewness'

Table S5. Feature list from different Selection Techniques from TA muscle EMG.

Ranking	Chi Square	mrmr	relieff	fscnca
1	'VL waveform length'	'VL waveform length'	'VL complexity'	'VL zero crossings'
2	'VL NSV'	'VL slope sign change'	'VL waveform length'	'VL waveform length'
3	'VL Wilson amplitude'	'VL AR3'	'VL NSV'	'VL slope sign change'
4	'VL AR2'	'VL Wilson amplitude'	'VL mobility'	'VL Wilson amplitude'
5	'VL AR3'	'VL NSV'	'VL AR1'	'VL mobility'
6	'VL zero crossings'	'VL zero crossings'	'VL AR3'	'VL skewness'
7	'VL mobility'	'VL skewness'	'VL AR2'	'VL AR3'
8	'VL skewness'	'VL mobility'	'VL slope sign change'	'VL AR1'
9	'VL slope sign change'	'VL complexity'	'VL zero crossings'	'VL AR2'
10	'VL complexity'	'VL AR1'	'VL Wilson amplitude'	'VL NSV'
11	'VL AR1'	'VL AR2'	'VL skewness'	'VL complexity'

Table S6. Feature list from different Selection Techniques from GL-VL muscles EMG.

Ranking	Chi Square	mrmr	relieff	fscnca

1	'GL Zero Order Moment'	'VL Sixth Order Moment'	'GL AR2'	'GL waveform length'
2	'VL Sixth Order Moment'	'GL Amplitude change 2'	'GL AR1'	'VL waveform length'
3	'GL Amplitude change 2'	'GL AR2'	'VL complexity'	'GL Wilson amplitude'
4	'GL AR2'	'VL slope sign change'	'GL Amplitude change 2'	'GL slope sign change'
5	'GL waveform length'	'GL skewness'	'VL AR3'	'VL zero crossings'
6	'VL waveform length'	'GL slope sign change'	'GL NSV'	'GL zero crossings'
7	'GL mobility'	'GL LMAV'	'VL AR4'	'VL Wilson amplitude'
8	'VL LMAV'	'VL Zero Order Moment'	'VL AR1'	'VL skewness'
9	'VL Wilson amplitude'	'VL Wilson amplitude'	'GL complexity'	'GL Sixth Order Moment'
10	'VL AR3'	'VL AR4'	'VL Wilson amplitude'	'GL Zero Order Moment'
11	'VL AR4'	'GL waveform length'	'GL waveform length'	'VL Fourth Order Moment'
12	'GL complexity'	'VL waveform length'	'VL zero crossings'	'GL AR2'
13	'GL Wilson amplitude'	'GL AR1'	'VL Zero Order Moment'	'GL skewness'
14	'GL AR3'	'VL zero crossings'	'VL Sixth Order Moment'	'GL AR4'
15	'VL zero crossings'	'GL zero crossings'	'VL slope sign change'	'GL AR3'
16	'VL AR1'	'GL Wilson amplitude'	'GL zero crossings'	'VL NSV'
17	'GL skewness'	'GL complexity'	'GL AR4'	'VL complexity'
18	'GL zero crossings'	'GL AR3'	'VL AR2'	'VL AR2'
19	'GL slope sign change'	'GL AR4'	'GL Wilson amplitude'	'VL AR4'
20	'VL skewness'	'VL skewness'	'GL AR3'	'GL mobility'
21	'VL slope sign change'	'VL mobility'	'VL skewness'	'VL mobility'
22	'VL complexity'	'VL complexity'	'GL slope sign change'	'GL complexity'
23	'GL AR4'	'VL AR2'	'GL skewness'	'VL AR3'
24	'VL AR2'	'VL AR3'		'VL slope sign change'

Table S7. Feature list from different Selection Techniques from GL-TA muscles EMG.

Ranking	Chi Square	mrmr	relieff	fscnca
1	'TA Zero Order Moment'	'TA LMAV'	'TA Sixth Order Moment'	'GL waveform length'
2	'GL Zero Order Moment'	'GL Amplitude change 2'	'TA AR1'	'TA waveform length'
3	'GL Amplitude change 2'	'GL AR2'	'TA Wilson amplitude'	'TA slope sign change'
4	'GL AR2'	'TA AR4'	'TA complexity'	'TA zero crossings'
5	'TA slope sign change'	'GL skewness'	'TA AR2'	'TA Wilson amplitude'
6	'GL waveform length'	'TA complexity'	'TA slope sign change'	'GL slope sign change'
7	'TA zero crossings'	'GL slope sign change'	'GL Sixth Order Moment'	'GL skewness'
8	'TA Wilson amplitude'	'TA AR2'	'TA zero crossings'	'GL Sixth Order Moment'
9	'GL mobility'	'GL LMAV'	'GL waveform length'	'GL AR1'
10	'TA skewness'	'TA slope sign change'	'GL mobility'	'TA skewness'
11	'TA AR1'	'GL waveform length'	'TA AR4'	'GL AR2'
12	'TA AR2'	'TA mobility'	'TA AR3'	'GL AR3'
13	'GL complexity'	'GL AR1'	'GL AR2'	'GL complexity'
14	'GL Wilson amplitude'	'TA zero crossings'	'GL AR4'	'TA complexity'
15	'TA AR4'	'GL zero crossings'	'GL complexity'	'GL LMAV'
16	'TA AR3'	'GL Wilson amplitude'	'TA skewness'	'TA AR2'
17	'GL AR3'	'GL complexity'	'GL zero crossings'	'TA AR3'
18	'GL skewness'	'GL AR3'	'GL Wilson amplitude'	'TA mobility'
19	'TA complexity'	'GL AR4'	'GL slope sign change'	'TA AR4'
20	'GL zero crossings'	'TA skewness'	'GL AR3'	'GL AR4'
21	'GL slope sign change'	'TA Wilson amplitude'	'GL skewness'	'GL zero crossings'
22	'GL AR4'	'TA AR3'		'GL Wilson amplitude'

Table S8. Feature list from different Selection Techniques from TA-VL muscles EMG.

Ranking	Chi Square	mrmr	relieff	fscnca
1	'TA Zero Order Moment'	'TA LMAV'	'TA Wilson amplitude'	'VL waveform length'
2	'VL Sixth Order Moment'	'VL Sixth Order Moment'	'TA zero crossings'	'VL skewness'
3	'TA slope sign change'	'TA AR4'	'TA Sixth Order Moment'	'TA waveform length'
4	'TA zero crossings'	'TA AR2'	'TA AR2'	'TA zero crossings'
5	'TA Wilson amplitude'	'VL Wilson amplitude'	'TA slope sign change'	'TA slope sign change'
6	'VL waveform length'	'VL AR4'	'TA AR4'	'TA Wilson amplitude'
7	'VL LMAV'	'TA slope sign change'	'TA AR1'	'VL slope sign change'
8	'VL Wilson amplitude'	'VL Zero Order Moment'	'VL complexity'	'VL Wilson amplitude'
9	'VL AR3'	'VL waveform length'	'TA complexity'	'VL Sixth Order Moment'
10	'TA skewness'	'VL slope sign change'	'VL AR2'	'TA skewness'
11	'TA AR1'	'TA mobility'	'VL AR4'	'VL Zero Order Moment'
12	'TA AR2'	'TA zero crossings'	'VL AR1'	'VL AR4'
13	'VL AR4'	'VL zero crossings'	'VL AR3'	'TA NSV'
14	'TA AR4'	'TA complexity'	'VL Zero Order Moment'	'TA AR3'
15	'TA AR3'	'TA skewness'	'VL slope sign change'	'TA AR2'
16	'VL zero crossings'	'TA Wilson amplitude'	'VL waveform length'	'TA mobility'
17	'VL AR1'	'TA AR3'	'TA AR3'	'VL AR1'
18	'TA complexity'	'VL skewness'	'VL zero crossings'	'TA complexity'
19	'VL skewness'	'VL mobility'	'TA skewness'	'TA AR4'
20	'VL slope sign change'	'VL complexity'	'VL Wilson amplitude'	'VL AR3'
21	'VL complexity'	'VL AR2'	'VL skewness'	'VL complexity'
22	'VL AR2'	'VL AR3'		'VL AR2'
23				'VL zero crossings'

Table S9. Feature list from different Selection Techniques from GL-VL-TA muscles EMG.

Ranking	Chi Square	mrmr	relieff	fscnca
1	'TA Zero Order Moment'	'TA LMAV'	'TA mobility'	'VL waveform length'
2	'GL Zero Order Moment'	'VL Sixth Order Moment'	'TA Sixth Order Moment'	'GL waveform length'
3	'VL Sixth Order Moment'	'GL Amplitude change 2'	'TA Wilson amplitude'	'VL skewness'
4	'GL Amplitude change 2'	'GL AR2'	'TA zero crossings'	'TA waveform length'
5	'GL AR2'	'TA AR4'	'TA AR2'	'VL zero crossings'
6	'TA slope sign change'	'GL skewness'	'TA complexity'	'TA Wilson amplitude'
7	'GL waveform length'	'TA AR2'	'GL Amplitude change 2'	'VL Wilson amplitude'
8	'TA zero crossings'	'VL Wilson amplitude'	'GL NSV'	'GL Wilson amplitude'
9	'TA Wilson amplitude'	'GL LMAV'	'GL waveform length'	'VL slope sign change'
10	'VL waveform length'	'GL slope sign change'	'TA slope sign change'	'GL zero crossings'
11	'GL mobility'	'VL Zero Order Moment'	'TA AR4'	'TA zero crossings'
12	'VL LMAV'	'TA slope sign change'	'GL AR1'	'TA slope sign change'
13	'VL Wilson amplitude'	'VL AR4'	'VL complexity'	'VL Zero Order Moment'
14	'VL AR3'	'GL waveform length'	'VL AR1'	'VL Fourth Order Moment'
15	'TA skewness'	'VL waveform length'	'TA AR3'	'GL Fourth Order Moment'
16	'TA AR1'	'VL slope sign change'	'VL AR4'	'TA Second Order Moment'
17	'TA AR2'	'TA mobility'	'VL Zero Order Moment'	'VL mobility'
18	'VL AR4'	'GL AR1'	'VL AR3'	'GL mobility'
19	'GL complexity'	'TA zero crossings'	'GL AR2'	'GL AR2'
20	'GL Wilson amplitude'	'VL zero crossings'	'GL AR4'	'TA skewness'
21	'TA AR4'	'TA complexity'	'GL complexity'	'TA AR1'

22	'TA AR3'	'GL zero crossings'	'TA skewness'	'TA AR2'
23	'GL AR3'	'GL Wilson amplitude'	'VL Wilson amplitude'	'VL AR2'
24	'VL zero crossings'	'GL complexity'	'VL slope sign change'	'TA AR3'
25	'VL AR1'	'GL AR3'	'GL zero crossings'	'VL AR4'
26	'GL skewness'	'GL AR4'	'VL zero crossings'	'GL AR4'
27	'TA complexity'	'TA skewness'	'VL AR2'	'TA complexity'
28	'GL zero crossings'	'TA Wilson amplitude'	'VL skewness'	'GL AR3'
29	'GL slope sign change'	'TA AR3'	'GL AR3'	'TA AR4'
30	'VL skewness'	'VL skewness'	'GL Wilson amplitude'	'GL slope sign change'
31	'VL slope sign change'	'VL mobility'	'GL slope sign change'	'VL complexity'
32	'VL complexity'	'VL complexity'	'GL skewness'	'GL skewness'
33	'GL AR4'	'VL AR2'		'VL AR3'
34	'VL AR2'	'VL AR3'		'GL complexity'

Table S10. Performance evaluation matrix using features from GL muscle EMG for different feature selection techniques.

**A**	**Chi Square feature Ranking Technique**				
**Incremental combination of Ranked features**	**Accuracy (%)**	**Precision (%)**	**Sensitivity (%)**	F1-Score (%)	AUC
Top 1	48.47	50.27	48.47	48.81	0.58
Top 2	66.79	67.22	66.79	66.65	0.66
Top 3	77.10	77.99	77.10	77.25	0.81
Top 4	80.53	81.52	80.53	80.69	0.81
Top 5	78.63	79.34	78.63	78.79	0.81
Top 6	80.92	81.77	80.92	81.03	0.81
Top 7	80.53	81.35	80.53	80.68	0.84
Top 8	81.30	82.22	81.30	81.54	0.83
Top 9	80.15	80.58	80.15	80.22	0.81
Top 10	80.53	81.42	80.53	80.76	0.82
Top 11	79.39	79.67	79.39	79.41	0.81
**B**	**mrmr feature Ranking Technique**				
**Incremental combination of Ranked features**	**Accuracy (%)**	**Precision (%)**	**Sensitivity (%)**	**F1-Score (%)**	**AUC**
Top 1	50.00	54.03	50.00	51.20	0.54
Top 2	64.50	65.60	64.50	64.69	0.66
Top 3	65.65	66.66	65.65	65.99	0.66
Top 4	68.32	69.65	68.32	68.65	0.70
Top 5	68.70	68.99	68.70	68.73	0.69
Top 6	69.08	69.61	69.08	69.28	0.70
Top 7	73.28	74.27	73.28	73.52	0.75
Top 8	75.95	76.05	75.95	75.90	0.77
Top 9	79.01	79.33	79.01	79.11	0.81
Top 10	78.24	78.36	78.24	78.29	0.80
Top 11	80.92	81.59	80.92	81.11	0.82
**C**	**relieff feature Ranking Technique**				

Incremental combination of Ranked features	Accuracy (%)	Precision (%)	Sensitivity (%)	F1-Score (%)	AUC
Top 1	45.42	51.10	45.42	47.32	0.62
Top 2	54.58	56.92	54.58	55.19	0.62
Top 3	73.66	74.82	73.66	74.03	0.77
Top 4	80.53	81.75	80.53	80.73	0.82
Top 5	80.15	80.58	80.15	80.20	0.80
Top 6	80.53	80.96	80.53	80.53	0.82
Top 7	84.73	84.89	84.73	84.76	0.85
Top 8	80.92	81.70	80.92	81.09	0.82
Top 9	81.68	82.09	81.68	81.74	0.83
Top 10	80.53	81.06	80.53	80.65	0.82
Top 11	80.92	81.26	80.92	81.04	0.82
**D**	**fscnca feature Ranking Technique**				
**Incremental combination of Ranked features**	**Accuracy (%)**	**Precision (%)**	**Sensitivity (%)**	**F1-Score (%)**	**AUC**
Top 1	39.31	44.00	39.31	40.68	0.57
Top 2	51.15	54.52	51.15	52.25	0.59
Top 3	56.11	56.79	56.11	56.36	0.64
Top 4	54.96	57.67	54.96	55.89	0.61
Top 5	65.27	67.52	65.27	65.91	0.71
Top 6	65.65	67.70	65.65	66.30	0.70
Top 7	75.57	76.33	75.57	75.81	0.79
Top 8	75.57	76.35	75.57	75.80	0.78
Top 9	75.57	77.36	75.57	76.02	0.78
Top 10	81.30	82.27	81.30	81.54	0.83
Top 11	77.48	78.50	77.48	77.75	0.79

Table S11. Performance evaluation matrix using features from VL muscle EMG for different feature selection techniques.

**A**	**Chi Square feature Ranking Technique**				
**Incremental combination of Ranked features**	**Accuracy (%)**	**Precision (%)**	**Sensitivity (%)**	F1-Score (%)	AUC
Top 1	48.85	53.54	48.85	50.38	0.61
Top 2	64.89	67.56	64.89	65.54	0.78
Top 3	59.92	63.38	59.92	60.61	0.73
Top 4	61.45	62.33	61.45	61.71	0.70
Top 5	68.32	69.99	68.32	68.81	0.73
Top 6	69.85	70.43	69.85	70.01	0.74
Top 7	72.90	74.46	72.90	73.48	0.82
Top 8	73.66	74.09	73.66	73.82	0.80
Top 9	67.56	69.85	67.56	68.32	0.75
Top 10	74.81	75.55	74.81	75.09	0.80
Top 11	72.90	73.93	72.90	73.25	0.80
**B**	**mrmr feature Ranking Technique**				

Incremental combination of Ranked features	Accuracy (%)	Precision (%)	Sensitivity (%)	F1-Score (%)	AUC
Top 1	49.62	54.16	49.62	51.13	0.59
Top 2	61.83	64.56	61.83	62.72	0.73
Top 3	61.45	63.29	61.45	62.05	0.71
Top 4	56.11	58.28	56.11	56.85	0.64
Top 5	61.45	63.94	61.45	62.41	0.72
Top 6	67.56	69.48	67.56	68.17	0.76
Top 7	65.65	66.65	65.65	66.06	0.72
Top 8	68.70	69.58	68.70	69.04	0.77
Top 9	70.23	71.64	70.23	70.78	0.78
Top 10	74.81	75.65	74.81	75.15	0.81
Top 11	75.95	76.77	75.95	76.24	0.82
**C**	**relieff feature Ranking Technique**				
**Incremental combination of Ranked features**	**Accuracy (%)**	**Precision (%)**	**Sensitivity (%)**	**F1-Score (%)**	**AUC**
Top 1	43.13	48.68	43.13	44.90	0.57
Top 2	58.78	63.06	58.78	60.36	0.71
Top 3	64.50	67.44	64.50	65.55	0.78
Top 4	69.08	70.33	69.08	69.61	0.76
Top 5	75.19	76.13	75.19	75.56	0.79
Top 6	74.81	75.74	74.81	75.15	0.83
Top 7	74.81	76.01	74.81	75.24	0.81
Top 8	77.10	77.96	77.10	77.39	0.83
Top 9	75.57	77.20	75.57	76.18	0.83
Top 10	77.10	78.74	77.10	77.65	0.81
Top 11	74.81	75.20	74.81	74.96	0.82
**D**	**fscnca feature Ranking Technique**				
**Incremental combination of Ranked features**	**Accuracy (%)**	**Precision (%)**	**Sensitivity (%)**	**F1-Score (%)**	**AUC**
Top 1	37.79	43.55	37.79	38.71	0.55
Top 2	50.38	53.77	50.38	51.60	0.62
Top 3	62.60	64.50	62.60	63.16	0.69
Top 4	62.60	63.49	62.60	62.84	0.66
Top 5	70.99	73.03	70.99	71.65	0.77
Top 6	67.56	67.61	67.56	67.58	0.74
Top 7	68.70	69.07	68.70	68.84	0.75
Top 8	69.47	70.17	69.47	69.64	0.78
Top 9	71.37	72.19	71.37	71.63	0.80
Top 10	72.90	73.96	72.90	73.27	0.81
Top 11	76.72	77.25	76.72	76.84	0.83

Table S12. Performance evaluation matrix using features from TA muscle EMG for different feature selection techniques.

**A**	**Chi Square feature Ranking Technique**				
**Incremental combination of Ranked features**	**Accuracy (%)**	**Precision (%)**	**Sensitivity (%)**	**F1-Score (%)**	**AUC**
Top 1	52.67	52.54	52.67	52.33	0.57
Top 2	59.92	60.22	59.92	60.05	0.61
Top 3	64.12	63.74	64.12	63.87	0.64
Top 4	69.47	69.70	69.47	69.57	0.71
Top 5	78.63	78.99	78.63	78.71	0.78
Top 6	75.19	75.77	75.19	75.41	0.74
Top 7	77.86	78.46	77.86	78.04	0.79
Top 8	80.53	80.91	80.53	80.59	0.82
Top 9	79.39	79.36	79.39	79.35	0.80
Top 10	77.48	78.45	77.48	77.75	0.79
Top 11	80.15	80.93	80.15	80.36	0.82
**B**	**mrmr feature Ranking Technique**				
**Incremental combination of Ranked features**	**Accuracy (%)**	**Precision (%)**	**Sensitivity (%)**	**F1-Score (%)**	**AUC**
Top 1	50.38	51.43	50.38	50.64	0.55
Top 2	55.73	55.72	55.73	55.72	0.60
Top 3	68.32	68.37	68.32	68.34	0.70
Top 4	79.01	79.12	79.01	79.03	0.79
Top 5	75.57	75.80	75.57	75.65	0.77
Top 6	77.48	77.93	77.48	77.59	0.79
Top 7	76.34	76.65	76.34	76.47	0.78
Top 8	78.63	79.13	78.63	78.82	0.79
Top 9	84.35	85.06	84.35	84.48	0.87
Top 10	79.77	80.16	79.77	79.86	0.82
Top 11	80.15	80.35	80.15	80.23	0.80
**C**	**relieff feature Ranking Technique**				
**Incremental combination of Ranked features**	**Accuracy (%)**	**Precision (%)**	**Sensitivity (%)**	**F1-Score (%)**	**AUC**
Top 1	53.44	54.69	53.44	53.63	0.61
Top 2	58.40	59.24	58.40	58.52	0.59
Top 3	67.18	67.37	67.18	67.21	0.73
Top 4	69.85	71.13	69.85	70.22	0.72
Top 5	76.72	76.90	76.72	76.79	0.79
Top 6	79.39	79.75	79.39	79.52	0.80
Top 7	80.92	81.38	80.92	81.07	0.82
Top 8	79.77	80.62	79.77	79.99	0.82
Top 9	81.30	81.58	81.30	81.40	0.82
Top 10	79.77	81.17	79.77	80.11	0.82
Top 11	79.39	79.70	79.39	79.46	0.83
**D**	**fscnca feature Ranking Technique**				

Incremental combination of Ranked features	Accuracy (%)	Precision (%)	Sensitivity (%)	F1-Score (%)	AUC
Top 1	48.85	51.41	48.85	49.38	0.59
Top 2	62.98	63.18	62.98	63.02	0.65
Top 3	62.60	63.57	62.60	62.83	0.68
Top 4	70.23	70.45	70.23	70.33	0.74
Top 5	70.99	71.39	70.99	71.15	0.72
Top 6	70.23	70.78	70.23	70.44	0.72
Top 7	71.37	72.03	71.37	71.57	0.74
Top 8	72.90	73.65	72.90	73.12	0.75
Top 9	79.01	79.05	79.01	79.01	0.78
Top 10	80.15	80.27	80.15	80.19	0.81
Top 11	83.59	83.93	83.59	83.67	0.86

Table S13. Performance evaluation matrix using features from GL and TA muscles EMG for different feature selection techniques.

A	Chi Square feature Ranking Technique				
**Incremental combination of Ranked features**	**Accuracy (%)**	**Precision (%)**	**Sensitivity (%)**	**F1-Score (%)**	**AUC**
Top 10	89.69	89.81	89.69	89.70	0.92
Top 11	90.84	90.91	90.84	90.84	0.91
Top 12	91.98	92.02	91.98	91.99	0.93
Top 13	93.51	93.70	93.51	93.52	0.95
Top 14	89.31	89.79	89.31	89.39	0.91
Top 15	95.80	96.04	95.80	95.84	0.96
Top 16	92.75	92.90	92.75	92.78	0.93
Top 17	91.98	92.04	91.98	92.00	0.92
Top 18	93.89	94.23	93.89	93.94	0.95
Top 19	91.98	92.04	91.98	92.00	0.93
Top 20	92.37	92.77	92.37	92.45	0.93
**B**	**mrmr feature Ranking Technique**				
**Incremental combination of Ranked features**	**Accuracy (%)**	**Precision (%)**	**Sensitivity (%)**	**F1-Score (%)**	**AUC**
Top 10	88.55	89.15	88.55	88.65	0.91
Top 11	91.22	91.72	91.22	91.29	0.92
Top 12	88.93	89.24	88.93	89.00	0.89
Top 13	90.84	91.56	90.84	90.95	0.92
Top 14	90.84	91.21	90.84	90.90	0.92
Top 15	89.69	90.06	89.69	89.74	0.91
Top 16	92.37	92.87	92.37	92.47	0.93
Top 17	94.27	94.38	94.27	94.30	0.95
Top 18	91.98	92.21	91.98	92.04	0.92
Top 19	91.60	91.68	91.60	91.63	0.92
Top 20	90.84	91.18	90.84	90.91	0.92
**C**	**relieff feature Ranking Technique**				

Incremental combination of Ranked features	Accuracy (%)	Precision (%)	Sensitivity (%)	F1-Score (%)	AUC
Top 10	89.69	90.12	89.69	89.79	0.90
Top 11	90.46	90.54	90.46	90.48	0.91
Top 12	90.08	90.37	90.08	90.13	0.92
Top 13	92.37	92.59	92.37	92.40	0.94
Top 14	92.37	92.91	92.37	92.46	0.95
Top 15	94.27	94.40	94.27	94.30	0.95
Top 16	91.98	92.13	91.98	92.03	0.92
Top 17	95.04	95.16	95.04	95.05	0.96
Top 18	91.60	91.94	91.60	91.67	0.92
Top 19	90.84	91.01	90.84	90.89	0.92
Top 20	93.51	93.56	93.51	93.53	0.93
**D**		**fscnca feature Ranking Technique**			
**Incremental combination of Ranked features**	**Accuracy (%)**	**Precision (%)**	**Sensitivity (%)**	**F1-Score (%)**	**AUC**
Top 12	84.73	85.30	84.73	84.87	0.86
Top 13	88.93	89.82	88.93	89.11	0.90
Top 14	90.84	91.67	90.84	90.96	0.93
Top 15	90.46	90.74	90.46	90.48	0.93
Top 16	92.37	92.73	92.37	92.45	0.93
Top 17	91.22	91.68	91.22	91.33	0.91
Top 18	88.93	89.35	88.93	89.04	0.89
Top 19	89.31	89.77	89.31	89.42	0.90
Top 20	91.22	91.51	91.22	91.29	0.92
Top 21	90.84	91.09	90.84	90.90	0.92
Top 22	93.51	93.67	93.51	93.55	0.94

Table S14. Performance evaluation matrix using features from GL and VL muscles EMG for different feature selection techniques.

**A**		**Chi Square feature Ranking Technique**			
**Incremental combination of Ranked features**	**Accuracy (%)**	**Precision (%)**	**Sensitivity (%)**	**F1-Score (%)**	**AUC**
Top 10	92.37	92.58	92.37	92.42	0.95
Top 11	95.04	95.31	95.04	95.08	0.97
Top 12	96.18	96.25	96.18	96.20	0.97
Top 13	94.66	95.00	94.66	94.73	0.97
Top 14	94.66	94.86	94.66	94.69	0.96
Top 15	95.42	95.52	95.42	95.44	0.97
Top 16	94.66	94.73	94.66	94.68	0.96
Top 17	93.51	93.80	93.51	93.54	0.97
Top 18	93.13	93.28	93.13	93.15	0.96
Top 19	91.98	92.17	91.98	92.00	0.96
Top 20	93.13	93.23	93.13	93.16	0.97
**B**		**mrmr feature Ranking Technique**			

Incremental combination of Ranked features	Accuracy (%)	Precision (%)	Sensitivity (%)	F1-Score (%)	AUC
Top 10	90.08	90.28	90.08	90.10	0.93
Top 11	91.22	91.45	91.22	91.25	0.95
Top 12	92.75	92.93	92.75	92.77	0.95
Top 13	93.51	93.51	93.51	93.51	0.95
Top 14	93.51	93.59	93.51	93.52	0.95
Top 15	93.51	93.54	93.51	93.52	0.94
Top 16	91.60	91.67	91.60	91.63	0.95
Top 17	91.98	92.15	91.98	92.00	0.95
Top 18	91.22	91.35	91.22	91.23	0.95
Top 19	90.84	91.14	90.84	90.90	0.95
Top 20	90.84	91.19	90.84	90.96	0.93
**C**			**relieff feature Ranking Technique**		
**Incremental combination of Ranked features**	**Accuracy (%)**	**Precision (%)**	**Sensitivity (%)**	**F1-Score (%)**	**AUC**
Top 10	92.75	92.99	92.75	92.79	0.94
Top 11	91.60	92.04	91.60	91.68	0.93
Top 12	92.37	92.60	92.37	92.42	0.94
Top 13	93.89	94.08	93.89	93.94	0.96
Top 14	95.04	95.31	95.04	95.08	0.97
Top 15	95.04	95.09	95.04	95.04	0.96
Top 16	95.80	95.84	95.80	95.81	0.97
Top 17	93.89	94.16	93.89	93.94	0.96
Top 18	95.80	95.84	95.80	95.81	0.99
Top 19	93.89	93.97	93.89	93.92	0.96
Top 20	93.51	93.74	93.51	93.56	0.96
**D**			**fscnca feature Ranking Technique**		
**Incremental combination of Ranked features**	**Accuracy (%)**	**Precision (%)**	**Sensitivity (%)**	**F1-Score (%)**	**AUC**
Top 14	88.93	89.03	88.93	88.96	0.92
Top 15	87.02	87.15	87.02	87.06	0.90
Top 16	89.69	89.79	89.69	89.73	0.92
Top 17	87.02	87.40	87.02	87.13	0.90
Top 18	90.08	90.18	90.08	90.12	0.91
Top 19	90.08	90.30	90.08	90.14	0.93
Top 20	92.37	92.57	92.37	92.40	0.96
Top 21	92.75	93.12	92.75	92.82	0.96
Top 22	90.84	90.99	90.84	90.84	0.94
Top 23	91.98	92.39	91.98	92.04	0.95
Top 24	91.60	92.11	91.60	91.66	0.95

Table S15. Performance evaluation matrix using features from TA and VL muscles EMG for different feature selection techniques.

A	Chi Square feature Ranking Technique				
**Incremental combination of Ranked features**	**Accuracy (%)**	**Precision (%)**	**Sensitivity (%)**	**F1-Score (%)**	**AUC**
Top 10	86.26	86.89	86.26	86.41	0.87
Top 11	85.88	86.35	85.88	86.00	0.86
Top 12	87.79	88.14	87.79	87.90	0.88
Top 13	86.26	86.65	86.26	86.39	0.86
Top 14	89.69	89.95	89.69	89.76	0.89
Top 15	91.22	91.67	91.22	91.34	0.92
Top 16	90.08	90.52	90.08	90.18	0.90
Top 17	88.93	89.20	88.93	89.01	0.89
Top 18	92.37	92.71	92.37	92.44	0.94
Top 19	89.69	90.41	89.69	89.85	0.91
Top 20	90.84	91.24	90.84	90.95	0.91
**B**	**mrmr feature Ranking Technique**				
**Incremental combination of Ranked features**	**Accuracy (%)**	**Precision (%)**	**Sensitivity (%)**	**F1-Score (%)**	**AUC**
Top 10	85.88	86.44	85.88	86.02	0.87
Top 11	87.79	88.23	87.79	87.89	0.89
Top 12	91.60	91.87	91.60	91.66	0.92
Top 13	91.98	92.07	91.98	92.01	0.91
Top 14	90.08	90.39	90.08	90.16	0.91
Top 15	90.46	90.73	90.46	90.52	0.91
Top 16	89.31	89.56	89.31	89.36	0.89
Top 17	90.08	90.52	90.08	90.18	0.90
Top 18	88.93	89.09	88.93	88.99	0.88
Top 19	90.46	90.95	90.46	90.59	0.91
Top 20	91.22	91.65	91.22	91.33	0.92
**C**	**relieff feature Ranking Technique**				
**Incremental combination of Ranked features**	**Accuracy (%)**	**Precision (%)**	**Sensitivity (%)**	**F1-Score (%)**	**AUC**
Top 10	85.88	86.59	85.88	86.10	0.87
Top 11	85.50	86.12	85.50	85.68	0.86
Top 12	90.08	90.76	90.08	90.22	0.91
Top 13	89.69	90.08	89.69	89.81	0.90
Top 14	91.98	92.08	91.98	92.01	0.92
Top 15	90.08	90.61	90.08	90.20	0.90
Top 16	90.08	90.32	90.08	90.16	0.90
Top 17	92.75	93.03	92.75	92.82	0.93
Top 18	91.22	91.73	91.22	91.35	0.91
Top 19	91.22	91.60	91.22	91.33	0.91
Top 20	90.84	91.36	90.84	90.96	0.91
**D**	**fscnca feature Ranking Technique**				

Incremental combination of Ranked features	Accuracy (%)	Precision (%)	Sensitivity (%)	F1-Score (%)	AUC
Top 12	79.77	80.36	79.77	79.95	0.82
Top 13	83.21	83.76	83.21	83.32	0.85
Top 14	86.64	86.70	86.64	86.66	0.88
Top 15	85.11	85.15	85.11	85.13	0.87
Top 16	89.31	89.47	89.31	89.36	0.90
Top 17	88.55	88.79	88.55	88.63	0.89
Top 18	87.79	88.41	87.79	87.93	0.87
Top 19	90.84	91.18	90.84	90.92	0.91
Top 20	93.51	93.80	93.51	93.58	0.94
Top 21	90.46	90.90	90.46	90.57	0.90
Top 22	90.46	90.59	90.46	90.50	0.90

Table S16. Performance evaluation matrix using features from GL, VL, and TA muscles EMG for different feature selection techniques.

**A**	**Chi Square feature Ranking Technique**				
**Incremental combination of Ranked features**	**Accuracy (%)**	**Precision (%)**	**Sensitivity (%)**	F1-Score (%)	AUC
Top 25	95.04	95.08	95.04	95.03	0.98
Top 26	94.27	94.35	94.27	94.26	0.95
Top 27	95.80	95.86	95.80	95.78	0.99
Top 28	94.27	94.38	94.27	94.25	0.97
Top 29	95.80	95.86	95.80	95.79	0.99
Top 30	93.51	93.72	93.51	93.50	0.99
Top 31	95.42	95.42	95.42	95.40	0.98
Top 32	94.27	94.30	94.27	94.26	0.98
Top 33	94.27	94.42	94.27	94.26	0.98
Top 34	95.42	95.55	95.42	95.41	0.99
Top 35	95.04	95.09	95.04	95.00	0.98
B	mrmr feature Ranking Technique				
Incremental combination of Ranked features	Accuracy (%)	Precision (%)	Sensitivity (%)	F1-Score (%)	AUC
Top 28	92.75	92.94	92.75	92.68	0.97
Top 29	92.37	92.46	92.37	92.34	0.95
Top 30	90.46	90.67	90.46	90.40	0.96
Top 31	91.60	91.60	91.60	91.59	0.95
Top 32	91.98	92.32	91.98	91.92	0.97
Top 33	94.27	94.43	94.27	94.24	0.99
Top 34	93.89	94.03	93.89	93.88	0.97
Top 35	94.66	94.67	94.66	94.65	0.98
Top 36	93.89	93.92	93.89	93.88	0.97
Top 37	93.13	93.16	93.13	93.12	0.97
Top 38	95.42	95.68	95.42	95.41	0.98
C	relieff feature Ranking Technique				
Incremental combination of Ranked features	Accuracy (%)	Precision (%)	Sensitivity (%)	F1-Score (%)	AUC

Top 20	91.98	92.03	91.98	91.98	0.97
Top 21	95.42	95.48	95.42	95.40	0.97
Top 22	92.75	92.85	92.75	92.71	0.97
Top 23	95.80	95.86	95.80	95.80	0.97
Top 24	93.13	93.23	93.13	93.09	0.97
Top 25	92.75	92.97	92.75	92.70	0.96
Top 26	93.51	93.55	93.51	93.49	0.96
Top 27	93.13	93.14	93.13	93.11	0.97
Top 28	93.89	93.87	93.89	93.85	0.98
Top 29	94.66	94.66	94.66	94.65	0.97
Top 30	93.89	93.90	93.89	93.87	0.97
D	fscnca feature Ranking Technique				
Incremental combination of Ranked features	Accuracy (%)	Precision (%)	Sensitivity (%)	F1-Score (%)	AUC
Top 28	93.89	93.97	93.89	93.87	0.98
Top 29	92.75	92.89	92.75	92.70	0.96
Top 30	94.66	94.71	94.66	94.64	0.97
Top 31	92.75	92.79	92.75	92.72	0.97
Top 32	93.51	93.58	93.51	93.50	0.97
Top 33	93.51	93.53	93.51	93.50	0.99
Top 34	93.89	93.92	93.89	93.86	0.97
Top 35	93.13	93.19	93.13	93.14	0.97
Top 36	95.04	95.04	95.04	95.03	0.98
Top 37	92.37	92.41	92.37	92.34	0.96
Top 38	95.04	95.15	95.04	95.01	0.97

Table S17. Feature list from different Selection Techniques from GRFx.

Ranking	Chi Square	mrmr	relieff	fscnca
1	'GRFx Enhanced Wave form length'	'GRFx Wave form length'	' GRFx Mean'	' GRFx Simple square integral'
2	' GRFx Quantile range'	' GRFx Irregularity Factor'	'GRFx Enhanced Wave form length'	' GRFx Peak to Peak'
3	' GRFx Myopluse percentage rate'	' GRFx Auto regressive 3'	' GRFx Myopluse percentage rate'	' GRFx Final/min'
4	'GRFx Wave form length'	' GRFx Sqrt of Variance'	'GRFx Wave form length'	' GRFx Quantile range'
5	' GRFx Mean'	' GRFx Max/min ratio'	' GRFx Slope sign change'	' GRFx Modified mean absolute value 1'
6	' GRFx Waveform length ratio'	' GRFx Spectral entropy'	' GRFx Quantile range'	'GRFx Enhanced Wave form length'
7	' GRFx Third quantile'	' GRFx Maximum Fractal length'	' GRFx Simple square integral'	' GRFx Modified mean absolute value 2'
8	' GRFx Slope sign change'	' GRFx Waveform length ratio'	'GRFx Enhanced mean Absolute Value'	' GRFx Difference Between 0th & 4th order moment'
9	' GRFx Median frequency '	' GRFx Slope sign change'	' GRFx Waveform length ratio'	' GRFx Myopluse percentage rate'
10	' GRFx Simple square integral'	' GRFx Difference Between 0th & 4th order moment'	' GRFx Sqrt of Variance'	' GRFx Mean frequency '

11	' GRFx Descriptor SE Upper Bound'	' GRFx Mean'	' GRFx Modified mean absolute value 2'	' GRFx Mean'
12	'GRFx Enhanced mean Absolute Value'	'GRFx Enhanced Wave form length'	' GRFx Final/min'	' GRFx Auto regressive 2'
13	' GRFx Final/min'	' GRFx Simple square integral'	' GRFx Third quantile'	'GRFx Enhanced mean Absolute Value'
14	' GRFx Coefficient of variation'	' GRFx Myopluse percentage rate'	' GRFx Modified mean absolute value 1'	' GRFx Sigma(signal entropy)'
15	' GRFx Max/min ratio'	' GRFx Modified mean absolute value 2'	' GRFx Peak to Peak'	' GRFx Total Energy'
16	' GRFx Spectral entropy'	' GRFx Estimate (signal entropy)'	' GRFx Median frequency '	' GRFx Average power'
17	' GRFx First quantile'	' GRFx Quantile range'	' GRFx Coefficient of variation'	' GRFx Max value '
18	' GRFx Estimate (signal entropy)'	'GRFx Enhanced mean Absolute Value'	' GRFx Irregularity Factor'	' GRFx Max ratio'
19	' GRFx Shape factor'	' GRFx Third quantile'	' GRFx Auto regressive 2'	' GRFx Willison amplitude'
20	' GRFx RMS'	'GRFx Mean absolute Value'	' GRFx First quantile'	' GRFx Descriptor SE Upper Bound'
21	' GRFx Irregularity Factor'	' GRFx Final/min'	' GRFx Zero order moment'	' GRFx Max/min ratio'
22	' GRFx Average power'	' GRFx Coefficient of variation'	' GRFx Mean frequency '	' GRFx Average amplitude change'
23	' GRFx Sqrt of Variance'	' GRFx First quantile'	' GRFx Total Energy'	' GRFx Time to peak'
24	' GRFx Sigma(signal entropy)'	' GRFx Auto regressive 4'	' GRFx Difference Between 0th & 4th order moment'	' GRFx Sparseness'
25	' GRFx Mean frequency '	' GRFx Peak'	' GRFx Average amplitude change'	' GRFx Log detector'
26	' GRFx Max value '	' GRFx Signal skewness'	' GRFx Max/min ratio'	' GRFx Shape factor'
27	' GRFx Auto regressive 2'	' GRFx Zero crossing'	' GRFx Max value '	' GRFx Integrated absolute value'
28	' GRFx Max frequency'	' GRFx RMS'	' GRFx RMS'	' GRFx Waveform length ratio'
29	' GRFx Peak'	' GRFx Average power'	' GRFx Log detector'	' GRFx Slope sign change'
30	' GRFx Difference Between 0th & 4th order moment'	' GRFx Integrated absolute value'	' GRFx Spectral entropy'	' GRFx Peak'
31	' GRFx Zero order moment'	' GRFx Max frequency'	' GRFx Time to peak'	' GRFx Zero crossing'
32	' GRFx Sparseness'	' GRFx Max value '	' GRFx Sigma(signal entropy)'	' GRFx Signal skewness'
33	' GRFx Maximum Fractal length'	' GRFx Signal Kurtosis'	' GRFx Sparseness'	'GRFx Wave form length'
34	' GRFx Peak to Peak'	' GRFx Shape factor'	' GRFx Average power'	' GRFx Max frequency'
35	' GRFx Zero crossing'	' GRFx Zero order moment'	' GRFx Difference absolute standard deviation '	' GRFx Descriptor SE Lower Bound'
36	' GRFx Average amplitude change'	' GRFx Max ratio'	' GRFx Max frequency'	' GRFx Standard Deviation'

Table S18. Feature list from different Selection Techniques from GRFy.

Ranking	Chi Square	mrmr	relieff	fscnca

1	'GRFy Enhanced mean Absolute Value'	'GRFy Enhanced mean Absolute Value'	'GRFy Enhanced mean Absolute Value'	'GRFy Enhanced mean Absolute Value'
2	' GRFy Modified mean absolute value 2'	' GRFy First quantile'	' GRFy Auto regressive 3'	' GRFy Time to peak'
3	' GRFy Final/min'	' GRFy Slope sign change'	' GRFy Modified mean absolute value 2'	' GRFy Zero crossing'
4	' GRFy Signal Kurtosis'	' GRFy Difference Between 0th & 2nd order moment'	' GRFy Final/min'	' GRFy Slope sign change'
5	' GRFy Mean'	' GRFy Average amplitude change'	' GRFy Signal Kurtosis'	' GRFy Total Energy'
6	' GRFy Difference Between 0th & 2nd order moment'	' GRFy Quantile range'	' GRFy Myopluse percentage rate'	' GRFy Auto regressive 3'
7	' GRFy Auto regressive 1'	' GRFy Shape factor'	' GRFy Zero crossing'	' GRFy Auto regressive 2'
8	' GRFy First quantile'	' GRFy Signal skewness'	' GRFy Slope sign change'	' GRFy Standard Deviation'
9	' GRFy Time to peak'	' GRFy Final/min'	' GRFy Mean'	' GRFy Average power'
10	' GRFy Descriptor SE NCELL'	' GRFy Myopluse percentage rate'	' GRFy Difference Between 0th & 2nd order moment'	' GRFy Modified mean absolute value 2'
11	' GRFy Max/min ratio'	' GRFy Difference Between 0th & 4th order moment'	' GRFy Third quantile'	' GRFy Maximum Fractal length'
12	' GRFy Sparseness'	' GRFy Mean'	' GRFy Time to peak'	' GRFy Final/min'
13	' GRFy Waveform length ratio'	' GRFy Modified mean absolute value 2'	' GRFy Descriptor SE Upper Bound'	'GRFy Wave form length'
14	' GRFy Myopluse percentage rate'	' GRFy Descriptor SE Upper Bound'	' GRFy Auto regressive 2'	'GRFy Mean absolute Value'
15	' GRFy Estimate (signal entropy)'	' GRFy Max/min ratio'	' GRFy First quantile'	' GRFy Zero order moment'
16	' GRFy Irregularity Factor'	' GRFy Signal Kurtosis'	' GRFy Signal skewness'	' GRFy Signal Kurtosis'
17	' GRFy Signal skewness'	' GRFy Auto regressive 1'	' GRFy Sparseness'	' GRFy Sqrt of Variance'
18	' GRFy Auto regressive 2'	'GRFy Wave form length'	' GRFy Willison amplitude'	' GRFy First quantile'
19	' GRFy Auto regressive 3'	' GRFy Irregularity Factor'	' GRFy Descriptor SE NCELL'	' GRFy Integrated absolute value'
20	' GRFy Coefficient of variation'	' GRFy Waveform length ratio'	' GRFy Auto regressive 1'	' GRFy Max/min ratio'
21	' GRFy Slope sign change'	'GRFy Enhanced Wave form length'	'GRFy Mean absolute Value'	'GRFy Enhanced Wave form length'
22	' GRFy Zero crossing'	' GRFy Descriptor SE NCELL'	' GRFy Standard Deviation'	' GRFy Willison amplitude'
23	'GRFy Wave form length'	' GRFy RMS'	'GRFy Wave form length'	' GRFy Shape factor'
24	' GRFy Auto regressive 4'	'GRFy Mean absolute Value'	'GRFy Enhanced Wave form length'	' GRFy Descriptor SE Lower Bound'
25	' GRFy Third quantile'	' GRFy Total Energy'	' GRFy Simple square integral'	' GRFy Estimate (signal entropy)'
26	' GRFy Descriptor SE Upper Bound'	' GRFy Descriptor SE Lower Bound'	' GRFy Peak to Peak'	' GRFy Descriptor SE Upper Bound'
27	' GRFy Modified mean absolute value 1'	' GRFy Zero order moment'	' GRFy Waveform length ratio'	' GRFy Mean'
28	' GRFy Integrated absolute value'	' GRFy Willison amplitude'	' GRFy Average power'	' GRFy Modified mean absolute value 1'
29	' GRFy Log detector'	' GRFy Peak to Peak'	' GRFy Maximum Fractal length'	' GRFy Difference Between 0th & 2nd order moment'

30	' GRFy Difference Between 0th & 4th order moment'	' GRFy Modified mean absolute value 1'	' GRFy Max/min ratio'	' GRFy Quantile range'
31	' GRFy Maximum Fractal length'	' GRFy Standard Deviation'	' GRFy Quantile range'	' GRFy Descriptor SE NCELL'
32	' GRFy Descriptor SE Lower Bound'	' GRFy Time to peak'	' GRFy Sqrt of Variance'	' GRFy Log detector'
33	' GRFy Total Energy'	' GRFy Integrated absolute value'	' GRFy Zero order moment'	' GRFy Difference absolute standard deviation '
34	' GRFy Difference absolute standard deviation '	' GRFy Auto regressive 4'	' GRFy Estimate (signal entropy)'	' GRFy Variance'
35	'GRFy Mean absolute Value'	' GRFy Sparseness'	' GRFy Sigma(signal entropy)'	' GRFy Auto regressive 4'
36	' GRFy Average power'	' GRFy Maximum Fractal length'	' GRFy Average amplitude change'	' GRFy Third quantile'

Table S19. Feature list from different Selection Techniques from GRFz.

Ranking	Chi Square	mrmr	relieff	fscnca
1	'GRFz Enhanced mean Absolute Value'	'GRFz Enhanced mean Absolute Value'	' GRFz Auto regressive 1'	'GRFz Enhanced mean Absolute Value'
2	' GRFz Auto regressive 2'	' GRFz Sigma(signal entropy)'	'GRFz Enhanced mean Absolute Value'	' GRFz Log detector'
3	' GRFz Difference Between 0th & 4th order moment'	' GRFz Willison amplitude'	' GRFz Difference absolute standard deviation '	' GRFz Descriptor SE Upper Bound'
4	' GRFz Log detector'	'GRFz Mean absolute Value'	' GRFz Auto regressive 2'	' GRFz Difference absolute standard deviation '
5	' GRFz Sigma(signal entropy)'	' GRFz Difference absolute standard deviation '	'GRFz Mean absolute Value'	' GRFz Willison amplitude'
6	' GRFz Auto regressive 4'	' GRFz Average power'	' GRFz Descriptor SE Upper Bound'	' GRFz Standard Deviation'
7	' GRFz Slope sign change'	' GRFz Log detector'	' GRFz Log detector'	' GRFz Average amplitude change'
8	' GRFz Zero crossing'	' GRFz Zero crossing'	' GRFz Standard Deviation'	' GRFz Auto regressive 3'
9	'GRFz Enhanced Wave form length'	' GRFz Sqrt of Variance'	' GRFz Zero order moment'	' GRFz Sqrt of Variance'
10	' GRFz Modified mean absolute value 1'	' GRFz Variance'	' GRFz Zero crossing'	' GRFz RMS'
11	' GRFz Auto regressive 1'	' GRFz Modified mean absolute value 2'	' GRFz Sigma(signal entropy)'	' GRFz Zero order moment'
12	' GRFz Willison amplitude'	'GRFz Enhanced Wave form length'	' GRFz Willison amplitude'	' GRFz Average power'
13	' GRFz Estimate (signal entropy)'	' GRFz Modified mean absolute value 1'	' GRFz Slope sign change'	'GRFz Wave form length'
14	' GRFz Zero order moment'	' GRFz Auto regressive 2'	' GRFz RMS'	' GRFz Integrated absolute value'

15	' GRFz Difference absolute standard deviation '	' GRFz Auto regressive 1'	' GRFz Average power'	' GRFz Modified mean absolute value 1'
16	' GRFz Descriptor SE Upper Bound'	' GRFz RMS'	' GRFz Total Energy'	' GRFz Modified mean absolute value 2'
17	' GRFz Shape factor'	' GRFz Estimate (signal entropy)'	' GRFz Peak to Peak'	'GRFz Mean absolute Value'
18	' GRFz Difference Between 0th & 2nd order moment'	' GRFz Peak to Peak'	' GRFz Integrated absolute value'	' GRFz Difference Between 0th & 2nd order moment'
19	' GRFz RMS'	' GRFz Slope sign change'	' GRFz Estimate (signal entropy)'	' GRFz Auto regressive 1'
20	'GRFz Mean absolute Value'	' GRFz Descriptor SE Upper Bound'	' GRFz Difference Between 0th & 4th order moment'	' GRFz Slope sign change'
21	' GRFz Modified mean absolute value 2'	' GRFz Difference Between 0th & 4th order moment'	' GRFz Average amplitude change'	' GRFz Difference Between 0th & 4th order moment'
22	' GRFz Integrated absolute value'	' GRFz Zero order moment'	' GRFz Descriptor SE NCELL'	'GRFz Enhanced Wave form length'
23	' GRFz Average power'	' GRFz Maximum Fractal length'	' GRFz Auto regressive 4'	' GRFz Descriptor SE Lower Bound'
24	' GRFz Peak to Peak'	' GRFz Total Energy'	'GRFz Enhanced Wave form length'	' GRFz Simple square integral'
25	' GRFz Standard Deviation'	' GRFz Shape factor'	' GRFz Modified mean absolute value 1'	' GRFz Zero crossing'
26	' GRFz Total Energy'	' GRFz Myopluse percentage rate'	' GRFz Auto regressive 3'	' GRFz Variance'
27	' GRFz Maximum Fractal length'	' GRFz Auto regressive 3'	' GRFz Sqrt of Variance'	' GRFz Maximum Fractal length'
28	' GRFz Descriptor SE NCELL'	' GRFz Average amplitude change'	' GRFz Descriptor SE Lower Bound'	' GRFz Descriptor SE NCELL'
29	' GRFz Variance'	' GRFz Simple square integral'	' GRFz Difference Between 0th & 2nd order moment'	' GRFz Sigma(signal entropy)'
30	' GRFz Auto regressive 3'	' GRFz Descriptor SE Lower Bound'	' GRFz Modified mean absolute value 2'	' GRFz Myopluse percentage rate'
31	' GRFz Average amplitude change'	' GRFz Descriptor SE NCELL'	' GRFz Maximum Fractal length'	' GRFz Shape factor'
32	' GRFz Sqrt of Variance'	' GRFz Difference Between 0th & 2nd order moment'	' GRFz Shape factor'	' GRFz Auto regressive 4'
33	' GRFz Descriptor SE Lower Bound'	'GRFz Wave form length'	'GRFz Wave form length'	' GRFz Peak to Peak'
34	'GRFz Wave form length'	' GRFz Standard Deviation'	' GRFz Simple square integral'	' GRFz Auto regressive 2'
35	' GRFz Simple square integral'	' GRFz Auto regressive 4'	' GRFz Variance'	' GRFz Estimate (signal entropy)'
36	' GRFz Myopluse percentage rate'	' GRFz Integrated absolute value'	' GRFz Myopluse percentage rate'	' GRFz Total Energy'

Table S20. Feature list from different Selection Techniques from GRFx-GRFy.

	Chi Square	mrmr	relieff	fscnca
1	' GRFy Integrated absolute value'	' GRFx Time to peak'	' GRFx Max Fractal length cD3'	' GRFx Max Fractal length cD8'
2	'GRFx Enhanced Wave form length'	' GRFy Max Fractal length cD1'	' GRFx Standard Deviation D2'	' GRFx PS kurtosis'
3	' GRFy Spectral slope'	' GRFy Zero order moment'	' GRFx Auto regressive 2'	' GRFx Standard Deviation cA8'
4	' GRFx Mean absolute Value D7'	' GRFy Band power of cD5'	' GRFy Difference Between 0th & 2nd order moment'	' GRFx Mean absolute value cD4'
5	' GRFy First peak '	' GRFx Mean absolute value cD5'	' GRFy Max Fractal length cD3'	'GRFy Wave form length'
6	' GRFx Mean absolute value cD2'	' GRFx Difference Between 0th & 2nd order moment'	' GRFx RMS D1'	' GRFx Waveform length cA8'
7	' GRFx Descriptor SE Lower Bound'	' GRFx Band power of cD1'	' GRFx Descriptor SE NCELL'	' GRFx Wavelet entropy level 6'
8	' GRFy Mean absolute value D3'	' GRFx RMS cD2'	' GRFx Standard Deviation A6'	' GRFy Mean absolute value D1'
9	' GRFx Mean absolute value D8'	' GRFy Waveform length cD7'	' GRFx Band power of cD7'	'GRFy Standard Deviation D5'
10	' GRFx Spectral slope'	' GRFx Standard Deviation cD7'	' GRFx Mean absolute value cD7'	' GRFy Integrated absolute value'
11	' GRFy Mean absolute value D1'	' GRFx Powe spectral (PS) skewness'	' GRFy Mean absolute value cD3'	' GRFx Mean frequency '
12	' GRFy Max Fractal length cD4'	' GRFy Standard Deviation cD8'	'GRFy Max Fractal length cA8'	' GRFy Descriptor SE Lower Bound'
13	' GRFy Spectral kurtosis'	' GRFx Zero order moment'	' GRFx Standard Deviation cD6'	' GRFy Spectral spread'
14	' GRFy Spectral decrease'	' GRFx Standard Deviation A6'	' GRFy Waveform length D1'	' GRFx RMS A6'
15	' GRFy Mean frequency '	' GRFy Band power of D8'	' GRFy Band power of cD6'	' GRFx Max Fractal length D6'
16	' GRFx Willison amplitude'	' GRFy Mean absolute value A8'	' GRFy Spectral deformation '	' GRFx Mean absolute value cD3'
17	' GRFx Wavelet entropy level 8'	' GRFy Descriptor SE Lower Bound'	' GRFy Max frequency'	' GRFx Standard Deviation D1'
18	' GRFx Descriptor SE NCELL'	' GRFy Modified mean absolute value 1'	' GRFx Mean absolute value cD6'	' GRFy Variance'
19	' GRFx Max Fractal length cD2'	' GRFx Standard Deviation'	' GRFy Max Fractal length cD8'	' GRFy Maximum Fractal length'
20	' GRFy Max Fractal length D1'	' GRFx Wavelet entropy level 6'	' GRFx Standard Deviation'	' GRFy PS median frequency'
21	' GRFy Max Fractal length D2'	' GRFx RMS cD1'	' GRFy Waveform length cD1'	' GRFy Waveform length cD7'
22	' GRFy Max Fractal length D5'	' GRFy Standard Deviation cD3'	' GRFx RMS cD1'	' GRFx RMS A8'
23	' GRFx Signal to motion artifact ratio'	' GRFx Mean absolute value A6'	' GRFy Estimate (signal entropy)'	' GRFy Band power of cD6'
24	' GRFy Second peak'	' GRFx RMS cD4'	' GRFx Waveform length cD3'	' GRFy Standard Deviation D1'

25	' GRFx Mean absolute value cA8'	' GRFx Waveform length cA8'	' GRFy Mean absolute value cD5'	' GRFx Sigma(signal entropy)'
26	' GRFy Spectral centroid'	' GRFy Difference Between 0th & 4th order moment'	' GRFx Mean absolute value D4'	' GRFx Mean absolute value A6'
27	' GRFy Powe spectral (PS) skewness'	' GRFy Spectral crest'	' GRFx Spectral centroid'	' GRFx Band power of D8'
28	' GRFx Band power of cD2'	' GRFx Max Fractal length D5'	' GRFy Waveform length cD8'	' GRFy Waveform length D7'
29	' GRFx Difference Between 0th & 4th order moment'	' GRFy Wavelet entropy level 6'	' GRFy Wavelet entropy level 8'	' GRFx Max to min drop of PS'
30	' GRFy Wavelet entropy level 4'	' GRFx Max Fractal length cD7'	' GRFx RMS cD4'	' GRFy Signal Kurtosis'
31	' GRFx Mean absolute value cD4'	' GRFy Standard Deviation D2'	' GRFy Band power of D7'	' GRFx Log detector'
32	' GRFy Band power of D2'	' GRFy Difference Between 0th & 2nd order moment'	' GRFx Difference Between 0th & 2nd order moment'	' GRFy Mean absolute value cD1'
33	' GRFx Wavelet entropy level 6'	' GRFy Max Fractal length cD8'	' GRFy Myopluse percentage rate'	'GRFx Enhanced Wave form length'
34	' GRFy Difference Between 0th & 4th order moment'	' GRFx Max Fractal length cD2'	' GRFy Mean absolute value D3'	' GRFy Shape factor'
35	' GRFy Signal to motion artifact ratio'	' GRFx Auto regressive 2'	' GRFx Mean absolute value A6'	' GRFy Band power of D3'
36	' GRFx Waveform length D2'	' GRFx Spectral spread'	' GRFy Wavelet entropy level 6'	' GRFx Waveform length D5'
37	' GRFy Max Fractal length cD2'	' GRFy Standard Deviation D8'	' GRFy Band power of cD3'	' GRFy Sigma(signal entropy)'
38	' GRFx Peak'	' GRFy Estimate (signal entropy)'	' GRFx Max Fractal length D5'	' GRFy Spectral entropy'
39	' GRFx Max ratio'	' GRFx RMS D6'	' GRFy Mean'	' GRFy RMS D4'
40	' GRFx First quantile'	' GRFx tandard Deviation D7'	' GRFx Mean absolute value cD4'	' GRFx Band power of cD4'
41	' GRFx Shape factor'	' GRFx Difference absolute standard deviation '	' GRFx Signal Kurtosis'	' GRFx Standard Deviation A8'
42	' GRFx Spectral roll off point '	' GRFy PS median frequency'	' GRFx Mean absolute value D2'	' GRFy Max Fractal length cD1'
43	' GRFx Signal Kurtosis'	' GRFy Zero crossing'	' GRFx Max Fractal length D8'	' GRFy Average power'
44	' GRFy Max frequency'	'GRFx Wave form length'	' GRFy Mean absolute value cA8'	' GRFy Waveform length D6'
45	' GRFy Shape factor'	' GRFx Modified mean absolute value 1'	' GRFx Modified mean absolute value 2'	' GRFx Mean absolute value cD1'
46	' GRFx Spectral skewness'	' GRFx RMS A8'	' GRFy Powe spectral (PS) skewness'	' GRFx RMS cD5'
47	' GRFx Max Fractal length D2'	' GRFx Waveform length D8'	' GRFx Max Fractal length D4'	' GRFx Band power of D1'
48	' GRFx Max Fractal length cD3'	' GRFy Mean'	' GRFy PS median frequency'	' GRFx Waveform length D6'
49	' GRFx First peak '	' GRFx Band power of D6'	' GRFx Mean absolute value D1'	' GRFy RMS cD6'

50	' GRFy Myopluse percentage rate'	' GRFy Spectral kurtosis'	' GRFx Spectral crest'	' GRFy Waveform length cD2'
51	' GRFy Max ratio'	' GRFy Spectral decrease'	' GRFx Standard Deviation cA8'	' GRFx Difference absolute standard deviation '
52	' GRFy Wavelet entropy level 8'	' GRFy Waveform length A8'	'GRFx Enhanced Wave form length'	' GRFx Shape factor'
53	' GRFy Spectral deformation '	' GRFx Willison amplitude'	' GRFx Waveform length D6'	' GRFy Standard Deviation cD5'
54	' GRFx Wavelet entropy level 4'	' GRFx Standard Deviation cD2'	' GRFy Standard Deviation cD6'	' GRFx Waveform length cD1'
55	' GRFy Sigma(signal entropy)'	' GRFy RMS D2'	' GRFx Max Fractal length D2'	' GRFx Third quantile'
56	' GRFx Max Fractal length cD4'	'GRFx Standard Deviation D5'	' GRFx First peak '	' GRFy PS kurtosis'
57	' GRFx Spectral flatness'	' GRFy Spectral spread'	' GRFy RMS cD6'	' GRFy Myopluse percentage rate'
58	' GRFy Time to peak'	' GRFy Mean absolute value D8'	' GRFy Shape factor'	' GRFx RMS cD2'
59	' GRFy Auto regressive 4'	' GRFy RMS'	' GRFy Waveform length D4'	' GRFy Mean absolute value cD3'
60	' GRFy Spectral roll off point '	' GRFy Max Fractal length A8'	' GRFy Mean absolute value A6'	' GRFx Max Fractal length D4'
61	' GRFy Signal Kurtosis'	' GRFx Band power of D7'	' GRFx Max Fractal length A8'	' GRFy Mean absolute value D3'
62	' GRFy Signal to noise ratio '	' GRFx PS mean frequency'	' GRFy Difference absolute standard deviation '	' GRFx Band power of D4'
63	' GRFy Final/min'	' GRFy Third quantile'	' GRFy Standard Deviation D4'	' GRFy Waveform length D8'
64	' GRFy Auto regressive 3'	' GRFy RMS cD4'	' GRFx Sqrt of Variance'	' GRFy RMS D7'
65	' GRFx Signal to noise ratio '	'GRFy Waveform length cD5'	' GRFy Mean absolute value D6'	' GRFx Waveform length cD6'
66	' GRFx Waveform length ratio'	' GRFx RMS D7'	' GRFy Modified mean absolute value 1'	' GRFx Difference Between 0th & 4th order moment'
67	' GRFx Signal skewness'	' GRFx Waveform length A6'	' GRFx Standard Deviation cD4'	' GRFx Band power of cD1'
68	' GRFy Slope sign change'	' GRFy Wavelet entropy level 4'	' GRFy Band power of D6'	' GRFx Second peak'
69	' GRFy Coefficient of variation'	' GRFy Sqrt of Variance'	' GRFx Band power of D3'	' GRFx Zero crossing'
70	' GRFy Max to min drop of PS'	'GRFx Waveform length cD5'	' GRFx Estimate (signal entropy)'	' GRFx Descriptor SE NCELL'
71	' GRFx Myopluse percentage rate'	' GRFx Band power of cD8'	' GRFy RMS D1'	' GRFy Max Fractal length A6'
72	' GRFy Peak'	' GRFx Signal to motion artifact ratio'	' GRFx Irregularity Factor'	' GRFx Signal skewness'
73	' GRFx Max Fractal length D1'	' GRFx Zero crossing'	' GRFx Quantile range'	'GRFy Waveform length cD5'
74	' GRFx Auto regressive 1'	' GRFy Mean absolute value D5'	' GRFy Max Fractal length D5'	' GRFx Max Fractal length D8'

75	' GRFx Max/min ratio'	' GRFx Waveform length D5'	' GRFy Band power of cD2'	' GRFx Descriptor SE Upper Bound'
76	' GRFx Max to min drop of PS'	' GRFy Standard Deviation cD1'	' GRFx Max Fractal length cD4'	' GRFx Powe spectral (PS) skewness'
77	' GRFx Final/min'	' GRFx Difference Between 0th & 4th order moment'	' GRFx RMS D4'	' GRFy Waveform length D5'
78	' GRFx Median frequency '	' GRFx Sigma(signal entropy)'	' GRFy Auto regressive 1'	' GRFx PS median frequency'
79	' GRFy Auto regressive 1'	' GRFy RMS cD5'	' GRFy Auto regressive 2'	' GRFx RMS D2'
80	' GRFx Max value '	' GRFy Average power'	' GRFy Spectral slope'	' GRFx First peak '
81	' GRFx LOC of second peak'	' GRFx Standard Deviation A8'	' GRFy Waveform length cD3'	' GRFy Standard Deviation D4'
82	' GRFx Coefficient of variation'	' GRFy Mean absolute value cD2'	' GRFx Mean absolute value cD5'	' GRFy RMS cD8'
83	' GRFx Irregularity Factor'	' GRFx Band power of D5'	' GRFy RMS D3'	' GRFy Difference Between 0th & 4th order moment'
84	' GRFx Band power of cD4'	' GRFy RMS cD7'	'GRFy Standard Deviation D6'	' GRFx Auto regressive 1'
85	' GRFy Wavelet entropy level 6'	' GRFx Mean absolute value cA8'	' GRFy Standard Deviation D2'	' GRFy Wavelet entropy level 6'
86	' GRFy Mean'	' GRFy Max Fractal length D8'	' GRFx Max Fractal length cD5'	' GRFy Descriptor SE NCELL'
87	' GRFy Auto regressive 2'	' GRFx Simple square integral'	' GRFx Waveform length cD8'	'GRFy Max Fractal length cA8'
88	' GRFx Max frequency'	' GRFx Mean absolute Value D7'	' GRFx Waveform length D4'	' GRFy RMS D5'
89	' GRFx PS kurtosis'	' GRFy Band power of D2'	' GRFy Mean absolute value D4'	' GRFx Max Fractal length D2'
90	' GRFy Max/min ratio'	' GRFy Variance'	'GRFx Enhanced mean Absolute Value'	' GRFy RMS D6'
91	' GRFy Sparseness'	' GRFx Band power of cD4'	' GRFy Spectral kurtosis'	' GRFy LOC of second peak'
92	' GRFx Auto regressive 2'	' GRFx Spectral deformation '	' GRFy Waveform length cA8'	' GRFy Zero order moment'
93	' GRFy Zero crossing'	' GRFy Spectral skewness'	' GRFy RMS cD1'	' GRFx Max Fractal length A8'
94	' GRFy Signal skewness'	' GRFy Standard Deviation A8'	' GRFx Band power of cA8'	' GRFy Standard Deviation cA8'
95	' GRFx Sparseness'	' GRFy First quantile'	' GRFy Auto regressive 4'	' GRFy Zero crossing'
96	' GRFy Irregularity Factor'	' GRFy RMS A8'	' GRFy Time to peak'	' GRFx Band power of cD3'
97	' GRFx Auto regressive 4'	' GRFy Spectral centroid'	'GRFx Standard Deviation D6'	' GRFx Spectral decrease'
98	' GRFx Spectral crest'	' GRFy Second peak'	' GRFx Mean absolute value A8'	' GRFx Max value '
99	' GRFx Auto regressive 3'	' GRFx Sqrt of Variance'	' GRFx RMS D8'	' GRFx Band power of D2'
100	' GRFx Sigma(signal entropy)'	' GRFy Band power of A8'		' GRFx Zero order moment'
101	' GRFy Waveform length ratio'	' GRFx Total Energy'		' GRFx Mean absolute value cD7'
102	' GRFy Spectral crest'	' GRFy Max Fractal length D6'		

103	' GRFx Zero crossing'	' GRFx Mean absolute value cD4'

Table S21. Feature list from different Selection Techniques from GRFx-GRFz.

Ranking	Chi Square	mrmr	relieff	fscnca
1	' GRFz Max Fractal length D3'	' GRFx RMS cD1'	' GRFz Spectral flatness'	' GRFz Waveform length cD1'
2	' GRFz Max/min ratio'	' GRFz RMS D6'	' GRFx Band power of cD5'	' GRFz Band power of D5'
3	' GRFz Third quantile'	' GRFz Waveform length cD2'	' GRFz Waveform length D7'	' GRFz Max Fractal length cD8'
4	' GRFz Average power'	' GRFz Waveform length cD7'	' GRFx Spectral slope'	' GRFx Max Fractal length cD4'
5	' GRFx Spectral crest'	' GRFx Band power of A8'	' GRFx Descriptor SE Lower Bound'	' GRFz Standard Deviation cD5'
6	' GRFx Waveform length cD1'	' GRFz Waveform length cD3'	' GRFx Spectral roll off point '	' GRFz RMS cD1'
7	' GRFz RMS cD8'	' GRFz Spectral roll off point '	' GRFx PS median frequency'	' GRFx Peak to Peak'
8	' GRFx Sqrt of Variance'	'GRFz Enhanced mean Absolute Value'	' GRFx Wavelet entropy level 8'	' GRFx Descriptor SE NCELL'
9	' GRFx Max ratio'	' GRFz Standard Deviation cD3'	' GRFx Spectral deformation '	' GRFx Mean absolute value cD7'
10	' GRFz Waveform length A8'	' GRFz Max Fractal length cD4'	' GRFz Difference Between 0th & 2nd order moment'	' GRFx Mean absolute value cD2'
11	' GRFz Spectral roll off point '	' GRFz tandard Deviation D7'	' GRFx Willison amplitude'	' GRFz RMS cD6'
12	' GRFx Waveform length cD7'	' GRFz Band power of A8'	' GRFz Spectral kurtosis'	' GRFz PS median frequency'
13	' GRFx Band power of cD6'	'GRFx Max Fractal length cA8'	' GRFx Mean absolute value cA8'	' GRFz Max Fractal length A8'
14	' GRFz First quantile'	' GRFz Standard Deviation A6'	' GRFz Auto regressive 2'	' GRFx Sqrt of Variance'
15	' GRFx Max value '	' GRFz Waveform length cD8'	' GRFz Mean absolute value cA8'	' GRFx Time to peak'
16	' GRFx Standard Deviation cD7'	' GRFx Willison amplitude'	' GRFz Max Fractal length D8'	' GRFz Waveform length D6'
17	' GRFx Time to peak'	' GRFx RMS cD4'	' GRFz Max Fractal length A8'	' GRFx Sigma(signal entropy)'
18	'GRFz Standard Deviation D6'	' GRFx Max Fractal length D7'	' GRFx Spectral flatness'	' GRFx Modified mean absolute value 2'
19	' GRFx Waveform length ratio'	' GRFz Spectral crest'	' GRFz Estimate (signal entropy)'	' GRFz Mean absolute value D8'
20	' GRFx Max Fractal length D6'	' GRFz Waveform length D8'	' GRFx Spectral kurtosis'	' GRFx Max Fractal length cD1'
21	' GRFz Standard Deviation A8'	' GRFz Difference Between 0th & 2nd order moment'	' GRFz Auto regressive 1'	' GRFz Band power of D3'

22	' GRFx Max Fractal length cD6'	' GRFx Standard Deviation D2'	' GRFx Signal to noise ratio '	' GRFx Difference Between 0th & 4th order moment'
23	' GRFx Standard Deviation cD5'	' GRFx Difference Between 0th & 2nd order moment'	'GRFx Standard Deviation D6'	' GRFz RMS D2'
24	' GRFz Max Fractal length cD2'	' GRFx Max Fractal length cD8'	' GRFx Max to min drop of PS'	' GRFz Band power of cA8'
25	' GRFx PS median frequency'	' GRFx Sqrt of Variance'	' GRFz Max Fractal length D1'	' GRFx Max value '
26	' GRFz RMS cD5'	' GRFz Total Energy'	' GRFx Auto regressive 4'	' GRFz Zero order moment'
27	'GRFz Enhanced mean Absolute Value'	' GRFz Band power of cA8'	' GRFz Sqrt of Variance'	'GRFx Enhanced Wave form length'
28	' GRFz Band power of D3'	' GRFx Standard Deviation A8'	' GRFx Difference Between 0th & 4th order moment'	' GRFx Difference Between 0th & 2nd order moment'
29	' GRFx Standard Deviation D2'	' GRFz Time to peak'	' GRFx First quantile'	' GRFx Max Fractal length cD8'
30	' GRFz Mean frequency '	' GRFz Mean absolute value cD1'	' GRFx Max Fractal length D2'	' GRFz Mean absolute Value D7'
31	' GRFz Spectral spread'	' GRFx Max/min ratio'	' GRFx Signal skewness'	' GRFx Powe spectral (PS) skewness'
32	' GRFz Difference Between 0th & 2nd order moment'	' GRFz Max value '	' GRFz Time to peak'	' GRFz Mean absolute value D6'
33	' GRFx Quantile range'	' GRFz Standard Deviation D4'	' GRFz Max Fractal length cD8'	' GRFx Average amplitude change'
34	' GRFx Max Fractal length D1'	' GRFx RMS cA8'	' GRFx Sigma(signal entropy)'	' GRFz Signal Kurtosis'
35	' GRFz Median frequency '	' GRFx Waveform length D5'	' GRFx Max Fractal length cD1'	' GRFx Waveform length D1'
36	' GRFz Mean absolute value D3'	' GRFz Waveform length cD4'	' GRFx Max value '	' GRFx Max/min ratio'
37	' GRFx RMS cD4'	' GRFx Mean absolute value D6'	' GRFx Mean absolute value cD2'	' GRFz PS kurtosis'
38	' GRFz Band power of D6'	' GRFz Mean absolute Value D7'	' GRFx Max Fractal length D3'	' GRFx RMS D7'
39	' GRFz RMS D2'	' GRFx Signal to noise ratio '	' GRFz Auto regressive 4'	' GRFz RMS cD2'
40	' GRFz Spectral entropy'	' GRFz Irregularity Factor'	' GRFz PS kurtosis'	' GRFx Quantile range'
41	' GRFz Peak'	' GRFz Band power of D7'	' GRFx Shape factor'	' GRFx tandard Deviation D7'
42	' GRFz Mean absolute value D5'	' GRFx RMS D3'	' GRFx Mean absolute value cD4'	' GRFz RMS A8'
43	' GRFx Mean absolute value cD5'	' GRFx Signal skewness'	' GRFz Final/min'	' GRFz RMS cD8'
44	' GRFx Mean absolute value D5'	' GRFx Waveform length A6'	' GRFz Spectral entropy'	' GRFz Max Fractal length D6'
45	' GRFz Mean absolute value D4'	' GRFx Waveform length cD7'	' GRFx RMS D2'	' GRFx Band power of D2'
46	' GRFx Mean absolute value A8'	' GRFz Median frequency '	' GRFz Band power of D8'	' GRFx Max frequency'

47	' GRFz Powe spectral (PS) skewness'	' GRFz Waveform length D3'	' GRFx Peak'	' GRFx First peak '
48	' GRFx Standard Deviation cD4'	' GRFx Waveform length A8'	' GRFx Wavelet entropy level 6'	' GRFz Mean absolute value cD1'
49	' GRFz Max Fractal length D7'	' GRFz Standard Deviation cD4'	' GRFz PS median frequency'	' GRFz Max Fractal length D7'
50	' GRFz Log detector'	' GRFz Standard Deviation cD2'	' GRFz Band power of cD7'	' GRFz Band power of A8'
51	' GRFz Peak to Peak'	' GRFx Estimate (signal entropy)'	' GRFz Max/min ratio'	' GRFz Max Fractal length D8'
52	' GRFx RMS A8'	' GRFz Peak'	' GRFx First peak '	' GRFz Auto regressive 4'
53	' GRFz Mean absolute value D2'	' GRFz Variance'	' GRFx Auto regressive 1'	' GRFz Time to peak'
54	' GRFz Standard Deviation cD5'	' GRFx Median frequency '	' GRFx Max/min ratio'	' GRFx Mean absolute value D2'
55	' GRFx Mean absolute value cD6'	' GRFx RMS D8'	' GRFz PS mean frequency'	' GRFx Standard Deviation D4'
56	' GRFz Band power of cD2'	' GRFx PS kurtosis'	' GRFx Max ratio'	' GRFz Descriptor SE NCELL'
57	' GRFz RMS A8'	' GRFx Band power of D5'	' GRFz Max Fractal length A6'	' GRFx Band power of A8'
58	' GRFz Standard Deviation D2'	' GRFx Standard Deviation cD6'	' GRFx Signal Kurtosis'	' GRFz Mean absolute value cD2'
59	' GRFz Band power of cD1'	' GRFx Waveform length cD6'	' GRFx Auto regressive 3'	' GRFx RMS cD2'
60	' GRFz Auto regressive 3'	' GRFz Mean absolute value cD3'	' GRFz First peak '	' GRFz RMS D7'
61	' GRFz Maximum Fractal length'	' GRFx Third quantile'	' GRFx Sparseness'	' GRFx Max Fractal length D8'
62	' GRFx Mean absolute value D3'	' GRFx Mean absolute value cD1'	' GRFx Coefficient of variation'	' GRFx Standard Deviation cD2'
63	' GRFx Mean absolute value cD7'	' GRFz Spectral skewness'	' GRFz Signal to noise ratio '	' GRFx Mean frequency '
64	' GRFz Descriptor SE Lower Bound'	' GRFz Waveform length cA8'	' GRFx Mean frequency '	' GRFz Standard Deviation cA8'
65	' GRFz Modified mean absolute value 2'	' GRFz Band power of A6'	' GRFz Third quantile'	' GRFx Signal Kurtosis'
66	' GRFx Mean absolute value cD8'	' GRFx Max Fractal length cD2'	' GRFx Standard Deviation cD1'	' GRFx Variance'
67	' GRFx Band power of D3'	'GRFz Standard Deviation D5'	' GRFx Wavelet entropy level 4'	' GRFx Band power of D4'
68	' GRFx Variance'	' GRFx Integrated absolute value'	' GRFx Auto regressive 2'	' GRFz RMS cD3'
69	' GRFz RMS D1'	' GRFx Band power of cD1'	' GRFx Final/min'	' GRFz Max value '
70	'GRFx Enhanced Wave form length'	' GRFx Band power of cD4'	' GRFx Waveform length ratio'	' GRFx Waveform length D3'
71	' GRFx Standard Deviation cA8'	' GRFz Descriptor SE Upper Bound'	' GRFz Sparseness'	' GRFx RMS cD3'
72	' GRFx Descriptor SE NCELL'	' GRFz Spectral spread'	' GRFz Waveform length ratio'	' GRFx Mean absolute value D6'

73	' GRFz Shape factor'	'GRFx Wave form length'	' GRFz Zero crossing'	' GRFx Band power of cD6'
74	' GRFx Descriptor SE Upper Bound'	' GRFx Second peak'	' GRFx Max Fractal length cD3'	' GRFz Waveform length cD8'
75	' GRFx Shape factor'	' GRFz Difference absolute standard deviation '	' GRFz Wavelet entropy level 8'	' GRFz Max to min drop of PS'
76	' GRFz Waveform length D2'	' GRFx Standard Deviation'	' GRFz Irregularity Factor'	' GRFx PS median frequency'
77	' GRFz Modified mean absolute value 1'	' GRFx Maximum Fractal length'	' GRFx Max Fractal length cD4'	' GRFz RMS D5'
78	' GRFx Sigma(signal entropy)'	' GRFz Spectral entropy'	' GRFx PS kurtosis'	' GRFx Mean absolute value D5'
79	' GRFx Spectral decrease'	' GRFx Spectral slope'	' GRFx Signal to motion artifact ratio'	' GRFx RMS D5'
80	' GRFx Waveform length D1'	' GRFx Descriptor SE Lower Bound'	' GRFx Second peak'	' GRFz Descriptor SE Lower Bound'
81	' GRFx RMS'	' GRFz Mean absolute value A6'	' GRFx Myopluse percentage rate'	' GRFx Max Fractal length cD5'
82	' GRFx Spectral deformation '	' GRFz Mean frequency '	' GRFx Zero crossing'	' GRFz Peak to Peak'
83	' GRFx RMS A6'	' GRFz Wavelet entropy level 4'	' GRFx Irregularity Factor'	' GRFz Max Fractal length cD5'
84	' GRFx Waveform length cA8'	' GRFx Max Fractal length cD3'	' GRFx Spectral crest'	' GRFz Log detector'
85	' GRFx Mean absolute value D6'	' GRFx Wavelet entropy level 4'	' GRFz Signal Kurtosis'	' GRFx Band power of cD3'
86	' GRFx Waveform length A8'	' GRFx Slope sign change'	' GRFx Max frequency'	' GRFx Waveform length D4'
87	' GRFz Spectral deformation '	' GRFx Myopluse percentage rate'		' GRFz Waveform length D8'
88	' GRFx RMS cA8'	' GRFz Mean absolute value D3'		' GRFx RMS cA8'
89	' GRFx Standard Deviation'	' GRFx Waveform length D7'		' GRFz Standard Deviation cD7'
90	' GRFz Band power of D5'	' GRFx Log detector'		' GRFz RMS A6'
91	' GRFx Band power of D5'	' GRFx Band power of D8'		' GRFz Sparseness'
92		' GRFx Coefficient of variation'		' GRFz Max Fractal length cD6'
93		' GRFx Band power of cD2'		' GRFz Wavelet entropy level 8'
94		' GRFx Modified mean absolute value 2'		

Table S22. Feature list from different Selection Techniques from GRFy-GRFz.

Rank ing	Chi Square	mrmr	relieff	fscnca
1	' GRFy Spectral skewness'	' GRFy Descriptor SE Upper Bound'	' GRFz Spectral flux'	' GRFy Waveform length D5'
2	' GRFy Irregularity Factor'	' GRFy Max Fractal length D5'	' GRFz RMS cD3'	' GRFz Mean absolute value A8'

3	' GRFy Spectral centroid'	' GRFy Max Fractal length cD5'	' GRFy RMS cD5'	'GRFz Enhanced Wave form length'
4	' GRFz Max Fractal length cD3'	'GRFy Waveform length cD5'	' GRFy Waveform length cD3'	' GRFz Waveform length cD4'
5	' GRFz Waveform length A6'	' GRFz Waveform length D7'	' GRFy Waveform length D3'	' GRFz RMS cD8'
6	' GRFz Max frequency'	' GRFz Max value '	' GRFz Descriptor SE Upper Bound'	' GRFy Band power of D7'
7	' GRFy Coefficient of variation'	' GRFz Band power of cD1'	'GRFy Wave form length'	' GRFz Spectral crest'
8	' GRFy Max ratio'	' GRFz Standard Deviation cD1'	' GRFy PS median frequency'	' GRFz Median frequency '
9	' GRFz Waveform length D4'	' GRFz Coefficient of variation'	' GRFy RMS'	' GRFy Waveform length ratio'
10	' GRFz Waveform length cD3'	'GRFz Wave form length'	' GRFy Standard Deviation cD2'	' GRFz Max Fractal length D6'
11	' GRFz Standard Deviation cD7'	' GRFz Max Fractal length cD3'	' GRFz Mean absolute Value D7'	' GRFy RMS cA8'
12	' GRFy Mean absolute value D6'	' GRFy Zero crossing'	' GRFz tandard Deviation D7'	' GRFz First peak '
13	' GRFy Signal Kurtosis'	' GRFz Mean absolute value A6'	' GRFz Wavelet entropy level 6'	' GRFy Waveform length D4'
14	' GRFz Band power of D4'	' GRFz Standard Deviation D1'	' GRFy RMS D2'	' GRFz Willison amplitude'
15	' GRFz Mean absolute value A6'	' GRFz Descriptor SE Upper Bound'	' GRFz Standard Deviation D4'	' GRFz Band power of cD5'
16	' GRFz RMS cA8'	'GRFz Mean absolute Value'	' GRFz Max Fractal length cD4'	' GRFy PS median frequency'
17	' GRFz Wavelet entropy level 6'	' GRFz Mean absolute value A8'	' GRFy Mean absolute value D8'	' GRFy Signal to noise ratio '
18	' GRFz Final/min'	' GRFy Standard Deviation D2'	' GRFz Zero crossing'	' GRFz Waveform length cD8'
19	' GRFz Band power of D2'	' GRFy Band power of D1'	' GRFy Median frequency '	' GRFz RMS cD1'
20	' GRFz Coefficient of variation'	' GRFz Mean absolute value D4'	' GRFy Standard Deviation D2'	' GRFz Zero crossing'
21	' GRFz Standard Deviation A6'	' GRFz Max Fractal length D1'	' GRFz Mean absolute value D2'	' GRFy Max frequency'
22	' GRFz Descriptor SE Upper Bound'	' GRFz Standard Deviation A6'	' GRFy Band power of cA8'	' GRFz Spectral deformation '
23	' GRFy RMS cD8'	' GRFz Average power'	' GRFz Waveform length cD8'	' GRFz Max value '
24	' GRFy Mean absolute value D4'	'GRFy Standard Deviation D5'	' GRFy PS mean frequency'	' GRFz RMS D1'
25	' GRFz Mean absolute value cA8'	' GRFz RMS cA8'	' GRFy Auto regressive 4'	' GRFy Mean absolute value cD8'
26	' GRFy Log detector'	' GRFz RMS A8'	' GRFy Mean absolute value cD6'	'GRFy Enhanced Wave form length'
27	' GRFy Sigma(signal entropy)'	' GRFz Band power of D6'	' GRFz Max Fractal length D6'	' GRFz RMS cD6'

28	' GRFy RMS D8'	' GRFy Spectral crest'	' GRFy Mean absolute value cA8'	'GRFy Mean absolute Value'
29	' GRFy Waveform length A6'	' GRFy Mean absolute value cD8'	' GRFy Difference Between 0th & 2nd order moment'	' GRFz Max frequency'
30	' GRFz Mean absolute Value D7'	' GRFz RMS cD6'	' GRFz Band power of A8'	' GRFy Standard Deviation cD8'
31	' GRFz Mean absolute value cD3'	' GRFz Standard Deviation'	' GRFz Band power of cD2'	' GRFz Max Fractal length D1'
32	' GRFy Band power of A6'	' GRFy RMS cD7'	' GRFz Mean'	' GRFz RMS D2'
33	' GRFz RMS cD2'	' GRFy Standard Deviation D8'	' GRFy Band power of A6'	' GRFy Band power of cD7'
34	' GRFz Spectral flatness'	' GRFz RMS D4'	' GRFz Peak'	' GRFy Zero order moment'
35	' GRFy Waveform length D5'	' GRFy Sqrt of Variance'	' GRFz Waveform length cD4'	' GRFz Mean absolute value D8'
36	' GRFy Band power of D8'	' GRFy Waveform length cD3'	' GRFy First quantile'	' GRFy RMS cD8'
37	' GRFy Spectral decrease'	' GRFz Max Fractal length A6'	' GRFy RMS cD2'	' GRFy Auto regressive 4'
38	' GRFz Waveform length A8'	' GRFy Mean absolute value cD1'	' GRFy Waveform length ratio'	' GRFy Sigma(signal entropy)'
39	' GRFy Max value '	' GRFz Standard Deviation D4'	' GRFy Max Fractal length cD4'	' GRFz Mean absolute value cD4'
40	' GRFy Mean absolute value D1'	' GRFz Sigma(signal entropy)'	' GRFz Waveform length cD3'	' GRFy Max Fractal length D3'
41	'GRFz Enhanced Wave form length'	' GRFy Modified mean absolute value 1'	' GRFz RMS cD2'	' GRFz Spectral decrease'
42	' GRFz Waveform length D2'	' GRFy Mean absolute value D5'	' GRFz Max Fractal length cD5'	' GRFy Spectral slope'
43	'GRFz Standard Deviation D5'	' GRFy Max Fractal length D7'	' GRFy Slope sign change'	' GRFy Band power of cD3'
44	' GRFz Quantile range'	' GRFy Modified mean absolute value 2'	' GRFz Mean absolute value D3'	' GRFz Auto regressive 3'
45	' GRFy Signal to noise ratio '	' GRFz Waveform length D6'	' GRFy Band power of D4'	' GRFy LOC of first peak'
46	' GRFz Band power of cD8'	' GRFy Irregularity Factor'	' GRFy Final/min'	' GRFz RMS A8'
47	' GRFz Band power of D1'	' GRFz Difference Between 0th & 2nd order moment'	' GRFz Mean frequency '	' GRFz Band power of D8'
48	' GRFy Standard Deviation A8'	' GRFz Mean'	' GRFy Sigma(signal entropy)'	' GRFy Standard Deviation cD1'
49	'GRFz Enhanced mean Absolute Value'	' GRFz Max Fractal length cD1'	' GRFz Band power of cD7'	' GRFy Max Fractal length D7'
50	' GRFy Standard Deviation cD7'	' GRFz RMS cD8'	' GRFz Wavelet entropy level 8'	' GRFy Integrated absolute value'
51	' GRFy Waveform length D6'	' GRFy Signal Kurtosis'	' GRFy LOC of first peak'	' GRFy Band power of cD1'
52	' GRFy Willison amplitude'	' GRFz Max Fractal length D4'	' GRFy Powe spectral (PS) skewness'	' GRFz Modified mean absolute value 2'

53	' GRFy Max Fractal length D8'	' GRFy Variance'	' GRFz Spectral roll off point '	' GRFz Signal Kurtosis'
54	' GRFy Mean frequency '	' GRFy Band power of D4'	' GRFz Descriptor SE Lower Bound'	' GRFy Mean'
55	' GRFy Final/min'	' GRFy Spectral centroid'	' GRFy Modified mean absolute value 1'	' GRFy Waveform length cD6'
56	' GRFy Max to min drop of PS'	' GRFy Wavelet entropy level 8'	' GRFy Average power'	' GRFz Wavelet entropy level 4'
57	' GRFy Difference absolute standard deviation '	' GRFy Band power of cD8'	' GRFz Total Energy'	' GRFz Max Fractal length D8'
58	' GRFz Max Fractal length cD2'	' GRFz Spectral spread'	' GRFy Sparseness'	' GRFy Estimate (signal entropy)'
59	' GRFy Standard Deviation cD2'	' GRFz Mean absolute Value D7'	' GRFy Max Fractal length cD8'	' GRFy RMS cD7'
60	' GRFz Signal Kurtosis'	' GRFy Estimate (signal entropy)'	' GRFy Mean absolute value A6'	' GRFz Standard Deviation D2'
61	' GRFz Max Fractal length cD5'	' GRFy Average power'	' GRFy Max Fractal length D5'	' GRFz Mean absolute value cA8'
62	' GRFz Waveform length cD7'	' GRFy Max Fractal length D4'	' GRFz Time to peak'	' GRFy Waveform length A6'
63	' GRFz Band power of cD7'	' GRFy LOC of second peak'	' GRFz Slope sign change'	' GRFz Difference Between 0th & 4th order moment'
64	' GRFz Difference Between 0th & 4th order moment'	' GRFy RMS A8'	' GRFz PS mean frequency'	' GRFz Max Fractal length cD6'
65	' GRFy Total Energy'	' GRFz Spectral flux'	' GRFz Auto regressive 1'	' GRFy Maximum Fractal length'
66	' GRFy Waveform length cA8'	' GRFz Peak to Peak'	' GRFz Max Fractal length cD8'	' GRFz RMS cA8'
67	' GRFy Band power of D5'	' GRFy Auto regressive 3'	' GRFy Descriptor SE NCELL'	' GRFz Waveform length D4'
68	' GRFy Standard Deviation cA8'	'GRFz Waveform length cD5'	' GRFz Signal to motion artifact ratio'	' GRFz Standard Deviation cA8'
69	' GRFz Standard Deviation cA8'	' GRFy Mean absolute value D4'	' GRFz First quantile'	' GRFy Band power of D1'
70	' GRFz Mean'	' GRFz Total Energy'	' GRFz Signal to noise ratio '	' GRFy Max Fractal length A6'
71	' GRFy RMS cD3'	' GRFz Waveform length D1'	' GRFz Median frequency '	' GRFy Mean absolute Value D7'
72	' GRFy Auto regressive 1'	' GRFz Spectral centroid'	' GRFy Quantile range'	' GRFz Descriptor SE Lower Bound'
73	' GRFy PS median frequency'	' GRFz Waveform length cA8'	' GRFz Average power'	' GRFy Max Fractal length cD2'
74	' GRFz Standard Deviation A8'	' GRFy Waveform length D2'	' GRFz Difference absolute standard deviation '	'GRFy Standard Deviation D5'
75	' GRFz Sigma(signal entropy)'	' GRFy RMS cD8'	' GRFy Wavelet entropy level 8'	' GRFz Waveform length cD2'

76	' GRFz Waveform length cA8'	' GRFz Wavelet entropy level 8'	' GRFy Log detector'	' GRFy Standard Deviation A8'
77	' GRFz Standard Deviation D1'	' GRFy Sparseness'	' GRFy Willison amplitude'	' GRFy Mean absolute value D8'
78	' GRFy Spectral flux'	' GRFz Mean absolute value cD3'	' GRFy Waveform length A6'	' GRFz Auto regressive 4'
79	' GRFy Waveform length cD2'	' GRFz Standard Deviation cD3'	' GRFz Max Fractal length cD3'	' GRFz RMS D3'
80	' GRFz Max Fractal length D4'	' GRFy Band power of D3'	' GRFy Max Fractal length D3'	' GRFz Band power of D6'
81		' GRFy RMS D6'		' GRFz Spectral kurtosis'
82		' GRFz Max Fractal length D7'		' GRFz Estimate (signal entropy)'
83		' GRFz Zero order moment'		' GRFz Waveform length D7'
84		' GRFz Sparseness'		' GRFz Max Fractal length A8'
85		' GRFy Third quantile'		' GRFz Auto regressive 2'
		'GRFy Mean absolute Value'		' GRFy Waveform length D1'

Table S23. Feature list from different Selection Techniques from GRFx-GRFy-GRFz.

Rank ing	Chi Square	mrmr	relieff	fscnca
1	' GRFx Signal Kurtosis'	' GRFy PS median frequency'	' GRFz Spectral flatness'	' GRFz Max Fractal length D6'
2	' GRFz Waveform length D4'	' GRFz Band power of A6'	' GRFy Max frequency'	' GRFy Median frequency '
3	' GRFz Max Fractal length cD8'	' GRFx Waveform length cD7'	' GRFx Simple square integral'	'GRFx Max Fractal length cA8'
4	' GRFx Willison amplitude'	' GRFx Mean absolute value D8'	' GRFx Spectral slope'	' GRFy Standard Deviation cD4'
5	' GRFy Irregularity Factor'	' GRFy Mean absolute value cD3'	' GRFz Waveform length D7'	' GRFy Zero order moment'
6	' GRFy Waveform length cD7'	' GRFx Mean absolute value D4'	' GRFx Spectral roll off point '	' GRFz Standard Deviation cD2'
7	' GRFz Mean'	' GRFx Waveform length D6'	' GRFx Descriptor SE Lower Bound'	' GRFx Spectral spread'
8	' GRFz Max Fractal length A6'	' GRFz Coefficient of variation'	' GRFx PS median frequency'	' GRFy Standard Deviation cD6'
9	' GRFx Sqrt of Variance'	' GRFx Standard Deviation cD3'	' GRFx Wavelet entropy level 8'	' GRFy Wavelet entropy level 6'
10	' GRFz Waveform length ratio'	' GRFz Peak'	' GRFz Spectral kurtosis'	' GRFx Spectral entropy'
11	' GRFx Band power of cD3'	' GRFy Max Fractal length cD8'	' GRFx Spectral deformation '	' GRFx Max/min ratio'
12	' GRFz RMS cD4'	' GRFx Sqrt of Variance'	' GRFx Mean absolute value cA8'	' GRFx Time to peak'
13	' GRFz Mean absolute value cD4'	' GRFy Standard Deviation'	' GRFz Difference Between 0th & 2nd order moment'	' GRFy Peak'

14	' GRFz Max Fractal length D8'	' GRFx First quantile'	' GRFy Spectral kurtosis'	' GRFz RMS D7'
15	' GRFy Spectral flux'	' GRFx Final/min'	' GRFx Willison amplitude'	' GRFx Band power of cA8'
16	' GRFx Sparseness'	' GRFy Waveform length D2'	' GRFz Max Fractal length D8'	' GRFx Simple square integral'
17	' GRFx Band power of D7'	' GRFx Mean absolute value cD8'	' GRFz Mean absolute value cA8'	' GRFz Standard Deviation D8'
18	' GRFz Log detector'	' GRFz Spectral crest'	' GRFx Spectral kurtosis'	' GRFz Descriptor SE NCELL'
19	' GRFz Standard Deviation cA8'	' GRFx Mean absolute value cD5'	' GRFz Max Fractal length A8'	' GRFy Waveform length ratio'
20	' GRFx Waveform length ratio'	' GRFz Spectral entropy'	' GRFx Spectral flatness'	' GRFz Difference absolute standard deviation '
21	' GRFz Waveform length D8'	' GRFx Waveform length A6'	' GRFz Auto regressive 2'	' GRFx Band power of cD7'
22	' GRFy Sqrt of Variance'	' GRFz Max Fractal length cD7'	' GRFz Estimate (signal entropy)'	' GRFy Signal to motion artifact ratio'
23	' GRFz PS kurtosis'	' GRFx Difference absolute standard deviation '	' GRFy Spectral centroid'	' GRFy Irregularity Factor'
24	' GRFy Final/min'	' GRFy Band power of A8'	' GRFy Peak'	' GRFz Mean absolute value cD4'
25	' GRFz Band power of cD4'	' GRFy Mean absolute value D4'	' GRFy Spectral deformation '	' GRFy Max Fractal length A8'
26	' GRFz Band power of cD2'	' GRFz First quantile'	' GRFx Max to min drop of PS'	' GRFx Waveform length D1'
27	' GRFx RMS cA8'	' GRFz Standard Deviation A8'	' GRFx Signal to noise ratio '	' GRFy Band power of cA8'
28	' GRFx Mean absolute Value D7'	' GRFx Max Fractal length D2'	' GRFy Mean absolute value cD7'	' GRFz Standard Deviation D1'
29	'GRFx Enhanced Wave form length'	' GRFx Mean absolute value cA8'	' GRFy Wavelet entropy level 8'	' GRFx Max Fractal length cD2'
30	' GRFz Integrated absolute value'	' GRFz Wavelet entropy level 6'	' GRFx First quantile'	' GRFy Descriptor SE NCELL'
31	' GRFx Time to peak'	' GRFx Max Fractal length D4'	' GRFz Auto regressive 1'	' GRFy Auto regressive 1'
32	' GRFx Max ratio'	' GRFx Spectral flatness'	'GRFx Standard Deviation D6'	' GRFz Waveform length D7'
33	' GRFx Mean absolute value cD3'	' GRFx Peak'	' GRFy Second peak'	' GRFx Sparseness'
34	' GRFz Band power of cD6'	' GRFx Max Fractal length cD7'	' GRFy Max/min ratio'	'GRFx Standard Deviation D6'
35	' GRFy Auto regressive 3'	' GRFx Maximum Fractal length'	' GRFy Signal skewness'	' GRFy Auto regressive 2'
36	' GRFx Standard Deviation D1'	' GRFx Mean absolute value cD6'	' GRFz Sqrt of Variance'	' GRFz Mean absolute value D4'
37	' GRFx First peak '	' GRFx Band power of D8'	' GRFy Mean frequency '	' GRFx Difference Between 0th & 2nd order moment'
38	' GRFy Spectral decrease'	' GRFz Waveform length cD4'	' GRFx Difference Between 0th & 4th order moment'	' GRFx Max Fractal length D6'

39	' GRFy Sparseness'	' GRFy Band power of cD1'	' GRFy LOC of first peak'	' GRFy Max/min ratio'
40	' GRFy RMS'	' GRFx Waveform length cD3'	' GRFx Signal skewness'	' GRFy Average amplitude change'
41	' GRFx Mean absolute value cD2'	' GRFy Spectral slope'	' GRFz Max Fractal length D1'	' GRFy Waveform length cD1'
42	' GRFx Spectral entropy'	' GRFx RMS D8'	' GRFz Time to peak'	' GRFx Zero crossing'
43	' GRFz Modified mean absolute value 2'	' GRFy Spectral flatness'	' GRFx Max Fractal length D2'	' GRFx Standard Deviation D2'
44	' GRFy Waveform length cD3'	' GRFy Estimate (signal entropy)'	' GRFx Sigma(signal entropy)'	' GRFz Peak to Peak'
45	' GRFy Auto regressive 1'	' GRFy Band power of cA8'	' GRFy Descriptor SE Lower Bound'	' GRFz RMS D6'
46	' GRFz Coefficient of variation'	' GRFz Max Fractal length A8'	' GRFx Mean absolute value cD2'	' GRFx RMS cD5'
47	' GRFz Wavelet entropy level 6'	' GRFz Signal skewness'	' GRFz PS kurtosis'	' GRFy Mean absolute value D6'
48	' GRFz Signal to noise ratio '	' GRFy Max Fractal length D1'	' GRFy Shape factor'	' GRFy RMS cD6'
49	' GRFx RMS D3'	' GRFx Descriptor SE NCELL'	' GRFz Max Fractal length cD8'	'GRFy Enhanced Wave form length'
50	' GRFy Second peak'	' GRFz Band power of D5'	' GRFx Auto regressive 4'	' GRFx Shape factor'
51	' GRFx Max Fractal length D2'	' GRFz Standard Deviation D3'	' GRFx Mean absolute value D3'	' GRFz Mean absolute value D2'
52	' GRFy Mean absolute value cD2'	' GRFz RMS cD7'	' GRFx Shape factor'	' GRFz Max Fractal length cD4'
53	' GRFy Max Fractal length D7'	' GRFy Band power of cD8'	' GRFx Max value '	' GRFx Mean absolute value cD5'
54	' GRFx Maximum Fractal length'	' GRFx RMS D3'	' GRFy Max to min drop of PS'	' GRFx Max Fractal length D4'
55	' GRFx Auto regressive 2'	' GRFx RMS cD7'	' GRFx Max Fractal length cD1'	' GRFx Willison amplitude'
56	' GRFz Spectral entropy'	' GRFy Max Fractal length D7'	' GRFx RMS D2'	' GRFy Band power of A6'
57	' GRFx Difference absolute standard deviation '	' GRFz RMS cD6'	' GRFx Mean absolute value cD4'	' GRFz Band power of D1'
58	' GRFy Max Fractal length cD4'	' GRFz Mean absolute Value D7'	' GRFx Wavelet entropy level 6'	' GRFx Max Fractal length cD5'
59	' GRFx Mean absolute value cD5'	' GRFx Spectral deformation '	' GRFz Final/min'	' GRFx Average power'
60	' GRFx Mean absolute value A8'	' GRFy Spectral crest'	' GRFz PS median frequency'	' GRFy Mean absolute value cD8'
61	' GRFx Waveform length cD2'	' GRFz Sqrt of Variance'	' GRFz Auto regressive 4'	' GRFy RMS D5'
62	' GRFx RMS D5'	' GRFz Band power of cD8'	' GRFx Peak'	' GRFx Modified mean absolute value 1'
63	' GRFx Waveform length A6'	' GRFz Average power'	' GRFz Standard Deviation cD7'	' GRFx Max Fractal length A8'

64	' GRFx Quantile range'	' GRFx RMS D5'	' GRFy Signal to motion artifact ratio'	' GRFz Max Fractal length cD7'
65	' GRFy Spectral flatness'	' GRFx Spectral roll off point '	' GRFz Band power of D8'	'GRFy Enhanced mean Absolute Value'
66	' GRFx Difference Between 0th & 4th order moment'	' GRFz Wavelet entropy level 8'	' GRFz Spectral entropy'	' GRFx Mean absolute value cA8'
67	' GRFz Descriptor SE NCELL'	' GRFz Band power of D1'	' GRFz First peak '	' GRFx Modified mean absolute value 2'
68	' GRFy Max Fractal length cD7'	' GRFy Max Fractal length cD1'	' GRFx Max ratio'	' GRFy RMS D6'
69	' GRFy Mean absolute value cD7'	' GRFy Waveform length cD8'	' GRFy Coefficient of variation'	' GRFx Maximum Fractal length'
70	' GRFy Mean absolute value cD8'	' GRFy Signal Kurtosis'	' GRFy Slope sign change'	' GRFx Max value '
71	' GRFy Auto regressive 4'	' GRFx Waveform length cD1'	' GRFx First peak '	' GRFz Spectral kurtosis'
72	' GRFy Waveform length cD1'	' GRFy Descriptor SE Lower Bound'	' GRFy Time to peak'	' GRFy Band power of cD3'
73	' GRFz Mean absolute value cD5'	' GRFy Peak to Peak'	' GRFx Max/min ratio'	' GRFy RMS A8'
74	' GRFy Slope sign change'	' GRFx Max Fractal length D1'	' GRFy Max Fractal length D3'	' GRFz Max Fractal length cD1'
75	' GRFy Wavelet entropy level 6'	' GRFx Mean absolute value cD2'	' GRFy Auto regressive 2'	' GRFy Waveform length D6'
76	' GRFy Standard Deviation A6'	' GRFz Waveform length ratio'	' GRFx Signal Kurtosis'	' GRFz RMS A6'
77	' GRFx Band power of D6'	' GRFy Max Fractal length D5'	' GRFx Coefficient of variation'	' GRFz PS kurtosis'
78	' GRFx Spectral crest'	' GRFx Band power of cD5'	' GRFx Standard Deviation cD1'	' GRFx Difference Between 0th & 4th order moment'
79	' GRFz Waveform length cD6'	' GRFy Mean absolute value D8'	' GRFy Auto regressive 1'	' GRFx Sigma(signal entropy)'
80	' GRFy Wavelet entropy level 8'	' GRFy Waveform length D4'	' GRFz Wavelet entropy level 8'	' GRFx Difference absolute standard deviation '
81	' GRFz Willison amplitude'	' GRFz Waveform length cA8'	' GRFz Max/min ratio'	'GRFy Max Fractal length cA8'
82	' GRFz Mean absolute value D2'	' GRFz Mean frequency '	' GRFy Waveform length ratio'	' GRFx Band power of cD8'
83	' GRFx Mean absolute value cD1'	' GRFy Band power of D2'	' GRFy Wavelet entropy level 4'	' GRFy Band power of D4'
84	' GRFy LOC of first peak'	' GRFx Standard Deviation D4'	' GRFz Max Fractal length A6'	' GRFx Max Fractal length cD7'
85	' GRFy Mean absolute value A8'	' GRFz Waveform length D1'	' GRFx Sparseness'	' GRFz Zero crossing'
86	' GRFz Average amplitude change'	' GRFx Max/min ratio'	' GRFx Auto regressive 1'	' GRFx Median frequency '
87	' GRFy tandard Deviation D7'	' GRFx Band power of D5'	' GRFx Mean frequency '	'GRFz Wave form length'
88	' GRFz Max Fractal length cD2'	' GRFz Quantile range'	' GRFx Waveform length ratio'	' GRFz Spectral flux'

89	' GRFx RMS D7'	' GRFy Band power of cD7'	' GRFy Auto regressive 4'	' GRFx Peak to Peak'
90	' GRFz Standard Deviation cD5'	' GRFx Max Fractal length cD3'	' GRFy Max Fractal length D2'	' GRFx Standard Deviation cD4'
91	'GRFx Mean absolute Value'	'GRFx Enhanced mean Absolute Value'	' GRFx Final/min'	' GRFy Mean absolute value cD3'
92	' GRFx Waveform length cD6'	' GRFx Wavelet entropy level 6'	' GRFy Max Fractal length cD4'	' GRFx Descriptor SE Upper Bound'
93	' GRFx Standard Deviation A6'	' GRFy Waveform length D8'	' GRFy Mean'	' GRFy Standard Deviation cD3'
94	' GRFy RMS cD2'	' GRFx Auto regressive 1'	' GRFz Signal to noise ratio '	' GRFx RMS D8'
95	' GRFz Mean absolute value D6'	' GRFz tandard Deviation D7'	' GRFz Third quantile'	' GRFz Mean'
96	' GRFx Sigma(signal entropy)'	' GRFz Log detector'	' GRFx Auto regressive 3'	' GRFy RMS cD5'
97	' GRFz Waveform length D7'	' GRFx Waveform length D2'	' GRFz Zero crossing'	' GRFz Max Fractal length cD6'
98	' GRFy Average amplitude change'	' GRFx RMS cD6'	' GRFy Max Fractal length cD2'	' GRFx Max Fractal length A6'
99	' GRFz Auto regressive 1'	' GRFy Max Fractal length cD5'	' GRFy Spectral crest'	' GRFy Waveform length cD7'
100	' GRFx Peak to Peak'	' GRFz Slope sign change'	' GRFy Signal to noise ratio '	' GRFx Band power of cD5'
101	' GRFx Mean absolute value cD8'	' GRFz Spectral decrease'	' GRFx Second peak'	' GRFy Standard Deviation A6'
102	' GRFx Waveform length cD1'	' GRFz Standard Deviation cD7'	' GRFz Waveform length ratio'	' GRFy Mean absolute value cD6'
103	' GRFx Wavelet entropy level 8'	' GRFx Standard Deviation cD1'	' GRFx Signal to motion artifact ratio'	' GRFz Wavelet entropy level 6'
104	' GRFz Band power of D7'	' GRFz RMS cA8'	' GRFx Wavelet entropy level 4'	' GRFy Standard Deviation'
105	' GRFz RMS cD3'	' GRFx Waveform length ratio'	' GRFy Waveform length cD2'	' GRFy Shape factor'
106	' GRFy LOC of second peak'	' GRFx PS kurtosis'	' GRFx PS kurtosis'	' GRFz Waveform length cD6'
107	' GRFy RMS cD6'	' GRFx RMS A6'	' GRFx Auto regressive 2'	' GRFx Mean absolute value cD7'
108	' GRFz Shape factor'	' GRFz Max Fractal length cD3'	' GRFz Sparseness'	' GRFy Max Fractal length D4'
109	' GRFy Signal skewness'	' GRFy Zero crossing'	' GRFy Sigma(signal entropy)'	' GRFy Waveform length cD8'
110	' GRFx Mean absolute value D6'	' GRFx Signal skewness'	' GRFy Zero crossing'	' GRFy First quantile'
111	' GRFx Standard Deviation cA8'	' GRFx Max Fractal length cD5'	' GRFx Max Fractal length cD4'	' GRFz Band power of cD5'
112	'GRFz Standard Deviation D6'	' GRFx Spectral centroid'	' GRFz Irregularity Factor'	' GRFy Mean absolute value cD1'
113	' GRFx Band power of cD4'	' GRFy Band power of cD2'	' GRFx Zero crossing'	' GRFx RMS cD2'

114	' GRFy Descriptor SE Lower Bound'	' GRFy Descriptor SE Upper Bound'	' GRFy Difference Between 0th & 4th order moment'	' GRFx First peak '
115	' GRFz RMS cD5'	' GRFz Waveform length cD7'	' GRFy Myopluse percentage rate'	' GRFz Mean absolute value cD6'
116	' GRFx RMS'	' GRFy PS kurtosis'	' GRFx Myopluse percentage rate'	'GRFz Mean absolute Value'
117	' GRFx Spectral decrease'	' GRFx Coefficient of variation'	' GRFy Max ratio'	' GRFx Standard Deviation cD2'
118	' GRFx Max Fractal length D8'	' GRFy Max ratio'	' GRFy Signal Kurtosis'	' GRFz Max Fractal length D8'
119	' GRFy Max Fractal length A6'	' GRFy Standard Deviation D8'	' GRFy Wavelet entropy level 6'	' GRFy Spectral flux'
120	' GRFx Integrated absolute value'	' GRFx Signal to motion artifact ratio'	' GRFy Max Fractal length cD1'	' GRFz Max Fractal length cD2'
121	' GRFy RMS cD8'	' GRFz Willison amplitude'	' GRFy Spectral roll off point '	' GRFx Standard Deviation cD1'
122	' GRFz Standard Deviation D1'	' GRFy Waveform length A8'	' GRFx Irregularity Factor'	' GRFx Waveform length cD4'
123	' GRFz Peak to Peak'	' GRFy Standard Deviation D4'	' GRFy Auto regressive 3'	' GRFx Powe spectral (PS) skewness'
124	' GRFy Max Fractal length D8'	' GRFz Waveform length D4'	' GRFx Spectral crest'	' GRFz Standard Deviation D4'
125	' GRFx Descriptor SE Lower Bound'	' GRFz RMS cD1'	' GRFy Final/min'	' GRFy Max Fractal length cD4'
126	' GRFz RMS D6'	' GRFx Standard Deviation D2'	' GRFy Sparseness'	' GRFx Max Fractal length D2'
127	' GRFx Waveform length cA8'	' GRFz Mean absolute value A6'	' GRFy Irregularity Factor'	' GRFz Spectral spread'
128	' GRFx Standard Deviation'	' GRFy Willison amplitude'	' GRFz Signal Kurtosis'	' GRFz Standard Deviation D2'
129	' GRFz Waveform length cD8'	'GRFz Standard Deviation D5'	' GRFx Max frequency'	' GRFx Band power of cD6'
130	'GRFy Enhanced Wave form length'	' GRFz RMS cD4'		' GRFy Standard Deviation cD8'
131	' GRFz Mean absolute value D3'	' GRFz Auto regressive 4'		' GRFy Average power'
132	' GRFx Band power of cD5'	' GRFy Time to peak'		' GRFy Maximum Fractal length'
133		' GRFx Band power of D7'		' GRFx Max ratio'
134		' GRFz Max Fractal length D4'		' GRFz Wavelet entropy level 8'
135		' GRFy Standard Deviation D1'		' GRFx Spectral flatness'
136		' GRFx Max Fractal length D7'		' GRFy Band power of D7'
137		' GRFy RMS D4'		' GRFz PS median frequency'
138		' GRFz Waveform length cD8'		' GRFx Spectral kurtosis'

139	' GRFx Max Fractal length D6'	

Table S24. Performance evaluation matrix using features from GRFx for different feature selection techniques.

A	Chi Square feature Ranking Technique				
**Incremental combination of Ranked features**	**Accuracy (%)**	**Precision (%)**	**Sensitivity (%)**	**F1-Score (%)**	**AUC**
Top 30	89.47	90.58	89.47	89.34	0.85
Top 31	92.11	93.20	92.11	92.01	0.87
Top 32	90.79	91.53	90.79	90.72	0.86
Top 33	86.84	87.95	86.84	86.89	0.83
Top 34	90.79	92.35	90.79	90.63	0.86
Top 35	90.79	91.08	90.79	90.59	0.87
Top 36	89.47	90.03	89.47	89.20	0.85
Top 37	89.47	89.57	89.47	89.33	0.86
Top 38	97.37	97.41	97.37	97.36	0.98
Top 39	94.74	95.16	94.74	94.78	0.94
B	mrmr feature Ranking Technique				
Incremental combination of Ranked features	Accuracy (%)	Precision (%)	Sensitivity (%)	F1-Score (%)	AUC
Top 45	93.42	93.36	93.42	93.37	0.91
Top 46	89.47	89.43	89.47	89.27	0.86
Top 47	93.42	93.54	93.42	93.32	0.93
Top 48	90.79	91.04	90.79	90.57	0.88
Top 49	90.79	91.03	90.79	90.52	0.87
Top 50	96.05	96.28	96.05	96.05	0.98
Top 51	94.74	95.08	94.74	94.79	0.94
Top 52	86.84	86.76	86.84	86.72	0.85
Top 53	90.79	91.35	90.79	90.78	0.91
Top 54	92.11	92.05	92.11	92.05	0.90
C	relieff feature Ranking Technique				
Incremental combination of Ranked features	Accuracy (%)	Precision (%)	Sensitivity (%)	F1-Score (%)	AUC
Top 10	88.16	88.24	88.16	88.18	0.86
Top 11	86.84	87.08	86.84	86.93	0.85
Top 12	89.47	89.67	89.47	89.51	0.89
Top 13	89.47	90.04	89.47	89.57	0.91
Top 14	97.37	97.41	97.37	97.36	0.98
Top 15	97.37	97.51	97.37	97.37	0.98
Top 16	93.42	93.71	93.42	93.41	0.92
Top 17	90.79	90.80	90.79	90.70	0.88
Top 18	94.74	94.89	94.74	94.75	0.95
Top 19	94.74	94.89	94.74	94.75	0.95
D	fscnca feature Ranking Technique				

Incremental combination of Ranked features	Accuracy (%)	Precision (%)	Sensitivity (%)	F1-Score (%)	AUC
Top 15	93.42	93.50	93.42	93.36	0.92
Top 16	94.74	94.89	94.74	94.75	0.93
Top 17	94.74	94.83	94.74	94.67	0.92
Top 18	93.42	93.91	93.42	93.30	0.90
Top 19	98.68	98.72	98.68	98.68	1.00
Top 20	94.74	94.71	94.74	94.70	0.94
Top 21	93.42	93.42	93.42	93.42	0.94
Top 22	92.11	92.09	92.11	92.07	0.90
Top 23	96.05	96.05	96.05	96.05	0.97
Top 24	97.37	97.41	97.37	97.36	0.98

Table S25. Performance evaluation matrix using features from GRFy muscle EMG for different feature selection techniques.

**A**	**Chi Square feature Ranking Technique**				
**Incremental combination of Ranked features**	**Accuracy (%)**	**Precision (%)**	**Sensitivity (%)**	F1-Score (%)	AUC
Top 10	90.79	91.19	90.79	90.80	0.94
Top 11	88.16	88.57	88.16	88.29	0.88
Top 12	85.53	86.00	85.53	85.59	0.87
Top 13	84.21	84.98	84.21	84.29	0.85
Top 14	88.16	88.60	88.16	88.19	0.90
Top 15	92.11	92.28	92.11	92.11	0.95
Top 16	84.21	84.98	84.21	84.29	0.88
Top 17	82.89	83.01	82.89	82.82	0.89
Top 18	86.84	86.87	86.84	86.80	0.88
Top 19	84.21	84.51	84.21	84.22	0.84
B	mrmr feature Ranking Technique				
Incremental combination of Ranked features	Accuracy (%)	Precision (%)	Sensitivity (%)	F1-Score (%)	AUC
Top 30	82.89	83.19	82.89	82.94	0.89
Top 31	80.26	80.46	80.26	80.28	0.85
Top 32	81.58	81.94	81.58	81.61	0.86
Top 33	84.21	84.85	84.21	84.27	0.86
Top 34	86.84	87.26	86.84	86.78	0.89
Top 35	78.95	80.31	78.95	79.07	0.84
Top 36	82.89	82.95	82.89	82.90	0.86
Top 37	84.21	84.51	84.21	84.22	0.85
Top 38	81.58	82.14	81.58	81.54	0.90
Top 39	81.58	83.91	81.58	81.64	0.85
C	relieff feature Ranking Technique				
Incremental combination of Ranked features	Accuracy (%)	Precision (%)	Sensitivity (%)	F1-Score (%)	AUC
Top 15	88.16	89.51	88.16	88.18	0.91

Top 16	89.47	90.64	89.47	89.62	0.91
Top 17	89.47	91.24	89.47	89.62	0.91
Top 18	90.79	92.08	90.79	90.91	0.92
Top 19	92.11	93.86	92.11	92.22	0.95
Top 20	89.47	91.24	89.47	89.62	0.91
Top 21	93.42	94.32	93.42	93.40	0.96
Top 22	92.11	92.98	92.11	92.20	0.92
Top 23	90.79	91.36	90.79	90.88	0.91
Top 24	89.47	90.39	89.47	89.60	0.89
D	fscnca feature Ranking Technique				
Incremental combination of Ranked features	Accuracy (%)	Precision (%)	Sensitivity (%)	F1-Score (%)	AUC
Top 20	89.47	89.50	89.47	89.43	0.89
Top 21	92.11	92.30	92.11	92.00	0.89
Top 22	90.79	91.03	90.79	90.56	0.87
Top 23	89.47	89.83	89.47	89.11	0.84
Top 24	94.74	94.75	94.74	94.72	0.95
Top 25	94.74	94.89	94.74	94.75	0.94
Top 26	93.42	93.53	93.42	93.45	0.93
Top 27	88.16	88.11	88.16	88.05	0.85
Top 28	92.11	92.27	92.11	92.12	0.91
Top 29	92.11	92.58	92.11	92.06	0.89

Table S26. Performance evaluation matrix using features from GRFz muscle EMG for different feature selection techniques.

**A**	**Chi Square feature Ranking Technique**				
**Incremental combination of Ranked features**	**Accuracy (%)**	**Precision (%)**	**Sensitivity (%)**	**F1-Score (%)**	**AUC**
Top 20	89.47	89.50	89.47	89.43	0.89
Top 21	92.11	92.30	92.11	92.00	0.89
Top 22	90.79	91.03	90.79	90.56	0.87
Top 23	89.47	89.83	89.47	89.11	0.84
Top 24	94.74	94.75	94.74	94.72	0.95
Top 25	94.74	94.89	94.74	94.75	0.94
Top 26	93.42	93.53	93.42	93.45	0.93
Top 27	88.16	88.11	88.16	88.05	0.85
Top 28	92.11	92.27	92.11	92.12	0.91
Top 29	92.11	92.58	92.11	92.06	0.89

B	mrmr feature Ranking Technique				
Incremental combination of Ranked features	Accuracy (%)	Precision (%)	Sensitivity (%)	F1-Score (%)	AUC
Top 10	88.16	88.26	88.16	88.15	0.88
Top 11	86.84	86.94	86.84	86.68	0.87
Top 12	89.47	89.66	89.47	89.42	0.88
Top 13	90.79	90.77	90.79	90.76	0.91

Top 14	90.79	90.80	90.79	90.70	0.89
Top 15	90.79	90.94	90.79	90.84	0.90
Top 16	88.16	88.28	88.16	88.20	0.91
Top 17	89.47	89.47	89.47	89.39	0.89
Top 18	93.42	93.47	93.42	93.42	0.94
Top 19	90.79	90.81	90.79	90.78	0.91

C	relieff feature Ranking Technique				
Incremental combination of Ranked features	Accuracy (%)	Precision (%)	Sensitivity (%)	F1-Score (%)	AUC
Top 26	90.79	91.05	90.79	90.77	0.88
Top 27	90.79	91.05	90.79	90.77	0.89
Top 28	90.79	90.80	90.79	90.70	0.88
Top 29	85.53	85.53	85.53	85.53	0.84
Top 30	92.11	92.09	92.11	92.07	0.91
Top 31	92.11	92.27	92.11	92.12	0.91
Top 32	93.42	93.71	93.42	93.41	0.93
Top 33	92.11	92.27	92.11	92.12	0.90
Top 34	89.47	89.64	89.47	89.50	0.87
Top 35	90.79	91.05	90.79	90.77	0.89
D	fscnca feature Ranking Technique				
Incremental combination of Ranked features	Accuracy (%)	Precision (%)	Sensitivity (%)	F1-Score (%)	AUC
Top 10	89.47	90.03	89.47	89.50	0.89
Top 11	90.79	91.19	90.79	90.84	0.91
Top 12	88.16	88.48	88.16	88.05	0.87
Top 13	93.42	93.65	93.42	93.45	0.95
Top 14	93.42	93.65	93.42	93.45	0.96
Top 15	90.79	91.19	90.79	90.84	0.91
Top 16	93.42	93.51	93.42	93.40	0.95
Top 17	93.42	93.51	93.42	93.40	0.94
Top 18	96.05	96.13	96.05	96.07	0.97
Top 19	96.05	96.13	96.05	96.07	0.97

Table S27. Performance evaluation matrix using features from GRFx and GRFy muscles EMG for different feature selection techniques.

**A**	**Chi Square feature Ranking Technique**				
**Incremental combination of Ranked features**	**Accuracy (%)**	**Precision (%)**	**Sensitivity (%)**	**F1-Score (%)**	**AUC**
Top 10	90.79	90.95	90.79	90.85	0.89
Top 11	89.47	90.30	89.47	89.48	0.90
Top 12	90.79	90.94	90.79	90.71	0.92
Top 13	92.11	92.18	92.11	92.08	0.92
Top 14	93.42	94.32	93.42	93.40	0.96
Top 15	92.11	92.18	92.11	92.08	0.96

Top 16	94.74	95.00	94.74	94.78	0.95
Top 17	96.05	96.24	96.05	96.07	0.98
Top 18	92.11	92.28	92.11	92.11	0.95
Top 19	93.42	93.68	93.42	93.48	0.95
Top 20	93.42	93.47	93.42	93.42	0.95
B	mrmr feature Ranking Technique				
Incremental combination of Ranked features	Accuracy (%)	Precision (%)	Sensitivity (%)	F1-Score (%)	AUC
Top 85	96.05	96.22	96.05	96.05	0.93
Top 86	94.74	95.09	94.74	94.73	0.91
Top 87	97.37	97.43	97.37	97.37	0.97
Top 88	96.05	96.22	96.05	96.05	0.94
Top 89	96.05	96.05	96.05	96.02	0.94
Top 90	94.74	94.86	94.74	94.72	0.94
Top 91	94.74	95.09	94.74	94.73	0.91
Top 92	94.74	94.83	94.74	94.67	0.91
Top 93	94.74	95.09	94.74	94.73	0.92
Top 94	94.74	95.09	94.74	94.73	0.91
C	relieff feature Ranking Technique				
Incremental combination of Ranked features	Accuracy (%)	Precision (%)	Sensitivity (%)	F1-Score (%)	AUC
Top 10	84.21	85.78	84.21	84.51	0.86
Top 11	85.53	86.21	85.53	85.61	0.83
Top 12	92.11	92.03	92.11	92.04	0.92
Top 13	94.74	94.73	94.74	94.70	0.94
Top 14	92.11	93.43	92.11	92.09	0.95
Top 15	90.79	91.19	90.79	90.80	0.93
Top 16	92.11	92.28	92.11	92.11	0.95
Top 17	96.05	96.07	96.05	96.04	0.99
Top 18	90.79	91.19	90.79	90.80	0.94
Top 19	90.79	91.08	90.79	90.85	0.92
D	fscnca feature Ranking Technique				
Incremental combination of Ranked features	Accuracy (%)	Precision (%)	Sensitivity (%)	F1-Score (%)	AUC
Top 20	96.05	96.21	96.05	96.04	0.95
Top 21	98.68	98.74	98.68	98.69	1.00
Top 22	97.37	97.43	97.37	97.37	0.97
Top 23	98.68	98.74	98.68	98.68	0.98
Top 24	100.00	100.00	100.00	100.00	1.00
Top 25	98.68	98.74	98.68	98.68	0.98
Top 26	97.37	97.60	97.37	97.36	0.95
Top 27	97.37	97.47	97.37	97.33	0.96
Top 28	97.37	97.51	97.37	97.37	0.98
Top 29	97.37	97.47	97.37	97.33	0.95

Table S28. Performance evaluation matrix using features from GRFx and GRFz muscles EMG for different feature selection techniques.

**A**	**Chi Square feature Ranking Technique**				
**Incremental combination of Ranked features**	**Accuracy (%)**	**Precision (%)**	**Sensitivity (%)**	**F1-Score (%)**	**AUC**
Top 20	93.42	94.03	93.42	93.14	0.90
Top 21	92.11	92.05	92.11	91.98	0.87
Top 22	89.47	89.87	89.47	88.87	0.82
Top 23	90.79	91.03	90.79	90.56	0.87
Top 24	94.74	94.93	94.74	94.75	0.96
Top 25	93.42	93.53	93.42	93.45	0.93
Top 26	94.74	94.89	94.74	94.75	0.95
Top 27	94.74	94.89	94.74	94.75	0.94
Top 28	90.79	90.84	90.79	90.79	0.90
Top 29	97.37	97.43	97.37	97.37	0.99
B	mrmr feature Ranking Technique				
Incremental combination of Ranked features	Accuracy (%)	Precision (%)	Sensitivity (%)	F1-Score (%)	AUC
Top 15	89.47	89.40	89.47	89.41	0.85
Top 16	92.11	92.09	92.11	92.07	0.91
Top 17	94.74	94.82	94.74	94.75	0.96
Top 18	94.74	94.75	94.74	94.72	0.93
Top 19	97.37	97.43	97.37	97.37	0.99
Top 20	94.74	94.86	94.74	94.77	0.95
Top 21	94.74	94.75	94.74	94.72	0.94
Top 22	93.42	93.42	93.42	93.42	0.92
Top 23	94.74	94.86	94.74	94.77	0.96
Top 24	93.42	93.50	93.42	93.36	0.92
C	relieff feature Ranking Technique				
Incremental combination of Ranked features	Accuracy (%)	Precision (%)	Sensitivity (%)	F1-Score (%)	AUC
Top 40	93.42	93.48	93.42	93.36	0.92
Top 41	98.68	98.72	98.68	98.68	1.00
Top 42	97.37	97.41	97.37	97.36	0.98
Top 43	96.05	96.21	96.05	96.04	0.96
Top 44	96.05	96.05	96.05	96.05	0.97
Top 45	97.37	97.41	97.37	97.36	0.98
Top 46	94.74	95.00	94.74	94.67	0.93
Top 47	97.37	97.41	97.37	97.36	0.98
Top 48	94.74	94.79	94.74	94.73	0.95
Top 49	96.05	96.05	96.05	96.05	0.97
D	fscnca feature Ranking Technique				
Incremental combination of Ranked features	Accuracy (%)	Precision (%)	Sensitivity (%)	F1-Score (%)	AUC
Top 5	93.42	93.56	93.42	93.47	0.92

Top 6	97.37	97.60	97.37	97.36	0.98
Top 7	98.68	98.72	98.68	98.68	0.98
Top 8	98.68	98.74	98.68	98.68	0.98
Top 9	98.68	98.74	98.68	98.68	0.98
Top 10	98.68	98.74	98.68	98.68	0.98
Top 11	97.37	97.41	97.37	97.36	0.98
Top 12	96.05	96.21	96.05	96.04	0.96
Top 13	96.05	96.37	96.05	96.05	0.95
Top 14	96.05	96.13	96.05	96.07	0.98

Table S29. Performance evaluation matrix using features from GRFy and GRFz muscles EMG for different feature selection techniques.

**A**	**Chi Square feature Ranking Technique**				
**Incremental combination of Ranked features**	**Accuracy (%)**	**Precision (%)**	**Sensitivity (%)**	F1-Score (%)	AUC
Top 1	88.16	89.21	88.16	88.34	0.87
Top 2	89.47	89.29	89.47	89.27	0.85
Top 3	94.74	94.83	94.74	94.67	0.92
Top 4	85.53	85.76	85.53	85.51	0.85
Top 5	88.16	88.30	88.16	88.21	0.86
Top 6	92.11	92.48	92.11	92.22	0.92
Top 7	92.11	92.20	92.11	92.13	0.91
Top 8	89.47	89.59	89.47	89.51	0.89
Top 9	88.16	88.16	88.16	88.16	0.87
Top 10	86.84	87.37	86.84	87.04	0.86
B	mrmr feature Ranking Technique				
Incremental combination of Ranked features	Accuracy (%)	Precision (%)	Sensitivity (%)	F1-Score (%)	AUC
Top 40	92.11	92.20	92.11	92.13	0.93
Top 41	90.79	91.24	90.79	90.86	0.91
Top 42	96.05	96.05	96.05	96.05	0.97
Top 43	90.79	91.59	90.79	90.90	0.93
Top 44	90.79	90.83	90.79	90.79	0.90
Top 45	86.84	87.01	86.84	86.87	0.86
Top 46	89.47	89.40	89.47	89.41	0.88
Top 47	88.16	88.59	88.16	88.23	0.88
Top 48	89.47	89.59	89.47	89.51	0.90
Top 49	90.79	91.08	90.79	90.85	0.92
Top 50	93.42	93.42	93.42	93.42	0.93
C	relieff feature Ranking Technique				
Incremental combination of Ranked features	Accuracy (%)	Precision (%)	Sensitivity (%)	F1-Score (%)	AUC
Top 40	92.11	92.28	92.11	92.17	0.93
Top 41	89.47	90.05	89.47	89.61	0.90
Top 42	89.47	90.04	89.47	89.57	0.91
Top 43	92.11	92.60	92.11	92.19	0.94

Top 44	90.79	91.13	90.79	90.88	0.92
Top 45	94.74	94.82	94.74	94.75	0.96
Top 46	93.42	93.68	93.42	93.48	0.95
Top 47	92.11	92.20	92.11	92.13	0.93
Top 48	90.79	91.13	90.79	90.88	0.91
Top 49	90.79	91.08	90.79	90.85	0.91
Top 50	89.47	89.59	89.47	89.51	0.89
D	fscnca feature Ranking Technique				
Incremental combination of Ranked features	Accuracy (%)	Precision (%)	Sensitivity (%)	F1-Score (%)	AUC
Top 15	93.42	93.47	93.42	93.42	0.96
Top 16	94.74	94.82	94.74	94.75	0.96
Top 17	93.42	93.68	93.42	93.46	0.95
Top 18	93.42	93.68	93.42	93.46	0.95
Top 19	93.42	93.42	93.42	93.42	0.93
Top 20	98.68	98.72	98.68	98.68	1.00
Top 21	96.05	96.13	96.05	96.07	0.97
Top 22	94.74	94.75	94.74	94.72	0.95
Top 23	92.11	92.20	92.11	92.13	0.93
Top 24	92.11	92.20	92.11	92.13	0.93
Top 25	96.05	96.13	96.05	96.03	0.98

Table S30. Performance evaluation matrix using features from GRFx, GRFy and GRFz muscles EMG for different feature selection techniques.

**A**	**Chi Square feature Ranking Technique**				
**Incremental combination of Ranked features**	**Accuracy (%)**	**Precision (%)**	**Sensitivity (%)**	**F1-Score (%)**	**AUC**
Top 110	93.42	93.50	93.42	93.36	0.93
Top 111	97.37	97.41	97.37	97.36	1.00
Top 112	93.42	93.42	93.42	93.42	0.99
Top 113	96.05	96.20	96.05	96.02	1.00
Top 114	97.37	97.51	97.37	97.37	1.00
Top 115	97.37	97.51	97.37	97.37	1.00
Top 116	98.68	98.72	98.68	98.68	1.00
Top 117	94.74	94.75	94.74	94.72	1.00
Top 118	93.42	93.51	93.42	93.40	0.96
Top 119	92.11	92.15	92.11	92.10	0.97
B	mrmr feature Ranking Technique				
Incremental combination of Ranked features	Accuracy (%)	Precision (%)	Sensitivity (%)	F1-Score (%)	AUC
Top 60	96.05	96.05	96.05	96.05	1.00
Top 61	96.05	96.05	96.05	96.05	0.99
Top 62	94.74	94.82	94.74	94.75	0.99
Top 63	93.42	93.42	93.42	93.42	1.00
Top 64	98.68	98.72	98.68	98.68	1.00
Top 65	94.74	94.82	94.74	94.75	0.99

Top 66	96.05	96.13	96.05	96.07	1.00
Top 67	93.42	93.42	93.42	93.42	0.99
Top 68	97.37	97.43	97.37	97.37	1.00
Top 69	97.37	97.41	97.37	97.36	1.00
C	relieff feature Ranking Technique				
Incremental combination of Ranked features	Accuracy (%)	Precision (%)	Sensitivity (%)	F1-Score (%)	AUC
Top 100	97.37	97.43	97.37	97.37	0.99
Top 101	96.05	96.13	96.05	96.07	1.00
Top 102	96.05	96.24	96.05	96.07	0.99
Top 103	96.05	96.13	96.05	96.07	1.00
Top 104	97.37	97.41	97.37	97.36	1.00
Top 105	97.37	97.43	97.37	97.37	1.00
Top 106	98.68	98.72	98.68	98.68	1.00
Top 107	94.74	94.75	94.74	94.72	0.99
Top 108	96.05	96.13	96.05	96.07	0.97
Top 109	96.05	96.05	96.05	96.05	0.99
Top 110	98.68	98.72	98.68	98.68	1.00
D	fscnca feature Ranking Technique				
Incremental combination of Ranked features	Accuracy (%)	Precision (%)	Sensitivity (%)	F1-Score (%)	AUC
Top 15	97.37	97.51	97.37	97.34	1.00
Top 16	97.37	97.51	97.37	97.34	1.00
Top 17	94.74	95.29	94.74	94.75	1.00
Top 18	93.42	93.64	93.42	93.43	1.00
Top 19	97.37	97.51	97.37	97.34	1.00
Top 20	97.37	97.51	97.37	97.34	1.00
Top 21	98.68	98.72	98.68	98.68	1.00
Top 22	98.68	98.72	98.68	98.68	1.00
Top 23	98.68	98.72	98.68	98.68	1.00
Top 24	98.68	98.72	98.68	98.68	1.00
Top 25	98.68	98.72	98.68	98.68	1.00
